# Spatial and Temporal Modulation of Cell Instructive Cues in a Filamentous Supramolecular Biomaterial

**DOI:** 10.1021/acsami.1c24114

**Published:** 2022-04-10

**Authors:** Ciqing Tong, Joeri A. J. Wondergem, Marijn van den Brink, Markus C. Kwakernaak, Ying Chen, Marco M. R. M. Hendrix, Ilja K. Voets, Erik H. J. Danen, Sylvia Le Dévédec, Doris Heinrich, Roxanne E. Kieltyka

**Affiliations:** † Department of Supramolecular and Biomaterials Chemistry, Leiden Institute of Chemistry, Leiden University, P.O. Box 9502, 2300 RA Leiden, The Netherlands; ‡ Biological and Soft Matter Physics, Huygens-Kamerlingh Onnes Laboratory, Leiden University, P.O. Box 9504, 2300 RA Leiden, The Netherlands; § Institute for Complex Molecular Systems, Eindhoven University of Technology, P.O. Box 513, 5600 MD Eindhoven, The Netherlands; ∥ Division of Drug Discovery and Safety, Leiden Academic Centre for Drug Research, Leiden University, P.O. Box 9502, 2333 CC Leiden, The Netherlands; ⊥ Institute for Bioprocessing and Analytical Measurement Techniques, Rosenhof 1, 37308 Heilbad Heiligenstadt, Germany; # Faculty for Mathematics and Natural Sciences, Technische Universität Ilmenau, 98693 Ilmenau, Germany

**Keywords:** supramolecular, hydrogels, photopatterning, dithiolane, 3D cell culture

## Abstract

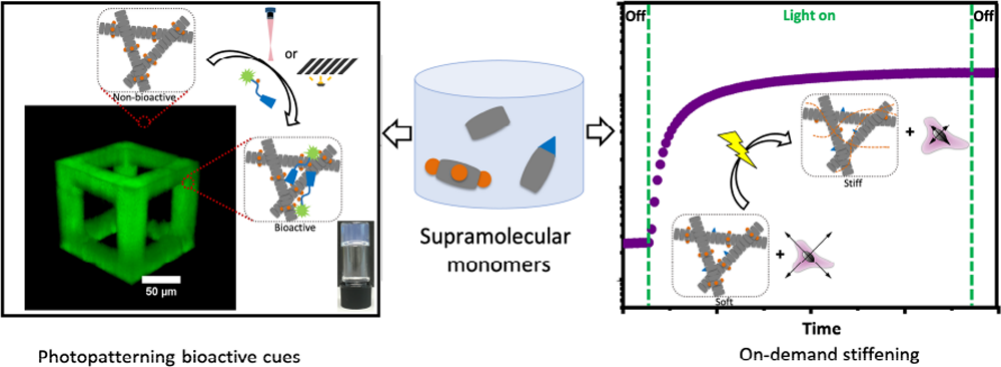

Supramolecular materials provide unique opportunities to mimic both the structure and mechanics of the biopolymer networks that compose the extracellular matrix. However, strategies to modify their filamentous structures in space and time in 3D cell culture to study cell behavior as encountered in development and disease are lacking. We herein disclose a multicomponent squaramide-based supramolecular material whose mechanics and bioactivity can be controlled by light through co-assembly of a 1,2-dithiolane (DT) monomer that forms disulfide cross-links. Remarkably, increases in storage modulus from ∼200 Pa to >10 kPa after stepwise photo-cross-linking can be realized without an initiator while retaining colorlessness and clarity. Moreover, viscoelasticity and plasticity of the supramolecular networks decrease upon photo-irradiation, reducing cellular protrusion formation and motility when performed at the onset of cell culture. When applied during 3D cell culture, force-mediated manipulation is impeded and cells move primarily along earlier formed channels in the materials. Additionally, we show photopatterning of peptide cues in 3D using either a photomask or direct laser writing. We demonstrate that these squaramide-based filamentous materials can be applied to the development of synthetic and biomimetic 3D *in vitro* cell and disease models, where their secondary cross-linking enables mechanical heterogeneity and shaping at multiple length scales.

## Introduction

Synthetic biomaterials that accurately mimic the cell micro-environment including its spatiotemporally evolving mechanical character are essential tools in the healthcare area to better understand disease and guide its treatment.^1−4^ While biological materials based on natural extracellular matrices (ECMs) or proteins have provided a wealth of information on cell behavior in their use as *in vitro* culture substrates, they lack the possibilities to fully decouple various matrix parameters (e.g., mechanics and bioactivity) and modulation of their properties *in situ* during culture.^5,6^ Alternatively, synthetic polymer hydrogels that mimic the water-rich character of native tissues offer boundless means to interrogate and guide cells undergoing complex biological processes through the ability to control their architectural and chemical features.^7^ In contrast to earlier strategies that relied on the use of covalent bonds to engineer gel mechanical properties such as stiffness,^8−11^ the field is shifting toward chemistries that are dynamic to obtain materials that can simulate the complex mechanical characteristics (e.g., viscoelasticity and nonlinear elasticity) of ECM proteins and tissues.^12−17^

Inspiration for the design of synthetic biomaterials can be derived from the fibrous proteins of the ECM, such as collagens, elastins, and fibronectins, that engage in a mechanical dialogue with cells.^18−22^ Collagen, a major component of human protein mass, forms filamentous protein networks with nonlinear elastic, viscoelastic, and viscoplastic mechanical features.^23−25^ Further cross-linking of these filaments defines the structure, stability, and mechanics of the ECM in various tissues and tissue states.^24^ For example, lysyl oxidases^26,27^ covalently cross-link collagen filaments in processes such as tissue maturation, but their aberrant overexpression is associated with diseases of the cardiovascular system and cancer; mammary tumor tissue can reach up to 10 kPa in stiffness and is mechanically heterogeneous, affecting integrin activation and tumor initiation.^28−30^ In addition to enzymes, cells can also modify the architecture of the ECM locally by exerting physical force on it resulting in its plastic deformation, further influencing cell migration and invasion.^30,59^ While recent works have gleaned insights into such remodeling processes in natural materials,^20,22,31^ fully synthetic approaches involving filamentous polymer materials that can provide these complex mechanical characteristics,^14,17^ their simultaneous decoupling from bioactive cues, and the potential to evolve them spatiotemporally in 3D culture as encountered in disease progression are rare. Access to such materials can provide unparalleled opportunities to better understand cell behavior in the context of a dynamic and heterogeneous mechanical environment that is inherent to the ECM in numerous developmental and disease processes.

Supramolecular polymers constructed from monomers that associate through non-covalent interactions, such as hydrogen-bonding, π-stacking, and hydrophobicity, provide access to filamentous structures that bear analogy to the biopolymers that compose the ECM.^32,33^ For some monomers above a critical concentration in water, hydrogel materials can be formed that are mechanically weak with self-recovering character having advantages for numerous applications in the biomedical area.^34,35^ To overcome their low stiffness, cross-linking of the supramolecular polymer filaments has been examined by introducing reactive groups on the monomers that can either ligate with themselves or through small molecules and polymers.^36−39^ Among these strategies, light-mediated ones are attractive as they provide opportunities for spatiotemporal control of the material properties.^35^ For example, internal polymerization of supramolecular polymer filaments during UV irradiation has been demonstrated to modulate gel stiffness, and further interfilament cross-linking can yield strain stiffening behavior.^40−44^ However, these approaches involve the use of reagents incompatible with cells, or give rise to materials that are highly colored, hindering possibilities to study cell behavior in an evolving mechanical environment during 3D culture. These roadblocks thus call for new alternatives to enable spatiotemporal control over their mechanics while permitting visualization by microscopic methods.

We earlier reported the formation of cytocompatible hydrogel materials from tripodal squaramide-based monomers that self-assemble into supramolecular filaments.^45^ Squaramides are minimalistic hydrogen bonding synthons that contain two C=O acceptors opposite two N–H donors on cyclobutenedione.^46^ When embedded within the hydrophobic domain of a flexible amphiphile, they hydrogen bond with one another in a head-to-tail hydrogen bonding array to form supramolecular polymers.^47−50^ Above a critical monomer concentration, soft and self-recovering hydrogels can be prepared, and their co-assembly with RGD-outfitted monomers influences cellular adhesion and proliferation.^51^ In order to modulate the hydrogel mechanics spatiotemporally as encountered in the ECM *in vivo* during disease, light-mediated cross-linking is a valuable strategy. We herein report the synthesis of a UV light-reactive squaramide-based monomer containing 1,2-dithiolanes, examine its co-assembly to prepare fully synthetic, non-proteolytic supramolecular hydrogels that exhibit complex mechanical characteristics, and explore their modulation with light in space and time for applications in 3D cell culture.

## Results and Discussion

### Synthesis of Tripodal Squaramide-Based Monomers SQ, SQ-DT, and SQ-RGD

**Figure 1:**
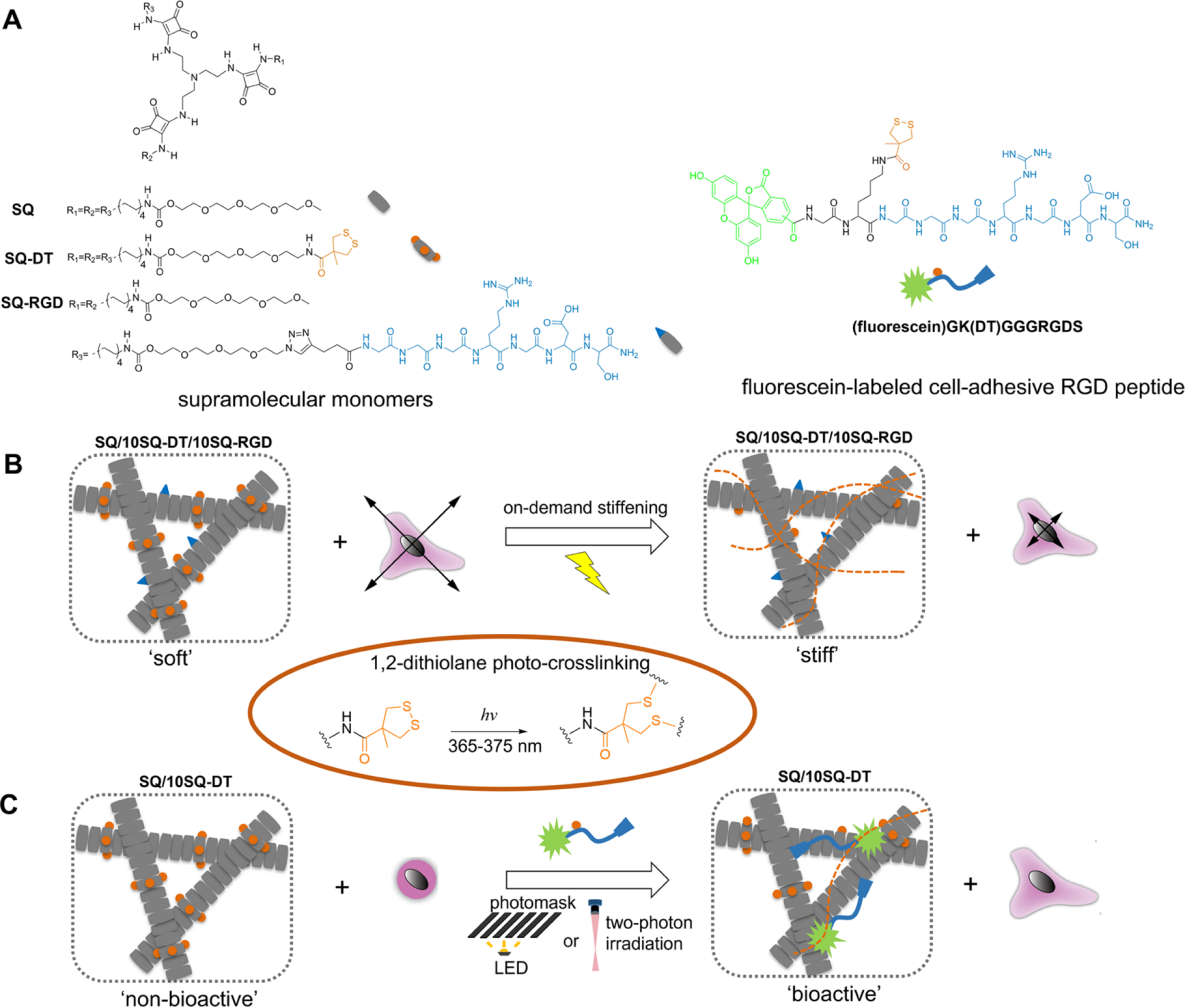
(A) Chemical structures of tripodal squaramide-based monomers (**SQ**, **SQ-DT**, and **SQ-RGD**) and a fluorescein-labeled cell-adhesive RGD peptide (**(fluorescein)GK(DT)GGGRGDS**) used to prepare multicomponent supramolecular hydrogels for 3D cell culture. (B) Schematic representation of the on-demand mechanical modulation of the multicomponent supramolecular materials (**SQ/10SQ-DT/10SQ-RGD**) (the numerical value denotes mole percentage) through light-activated 1,2-dithiolane cross-linking and its effect on cell migration in 3D. (C) Schematic representation of the spatial and temporal photopatterning of the bioactive RGD peptide **(fluorescein)GK(DT)GGGRGDS)** in **SQ-DT** hydrogels through a photomask or direct laser writing, and the associated cell morphologies (round or spread). In the case of photomask illumination, UV irradiation was applied using a benchtop LED source (∼10 mW/cm^2^, 375 nm). The orange dashed line indicates cross-linking of the 1,2-dithiolane units in the supramolecular materials.

To prepare supramolecular hydrogels whose properties can be modulated in space and time in 3D cell culture, a multicomponent approach is necessary to be able to cross-link the materials and provide bioactive cues. The native tripodal squaramide gelator, **SQ**, consists of a hydrophobic domain with three squaramide moieties attached to a tris(2-aminoethylamine) (TREN) core and aliphatic chains, and a hydrophilic domain composed of tetraethyleneglycol chains to guide its self-assembly into fibrillar supramolecular polymers and gel phase materials in water (Figure 1A).^45^ A second monomer, **SQ-DT**, containing a cross-linkable 1,2-dithiolane (DT) moiety on the periphery of the amphiphile was also prepared starting from methyl asparagusic acid to engender cross-linking between supramolecular polymers. Photoactivation of the DT moiety is achieved at the maximum of its absorbance peak at 330 nm.^52−55^ Finally, a third squaramide monomer, **SQ-RGD**, was synthesized where one arm of **SQ** was modified with an RGD peptide, to engage integrin receptors on the cell surface to the supramolecular material.^51^ Synthetic procedures for **SQ-DT** and **SQ-RGD** can be found in the Supporting Information.

### Multicomponent Supramolecular Hydrogel Preparation

We previously demonstrated that the monomer **SQ** can form transparent hydrogels through sonication and has a critical gelation concentration (CGC) in the range of 4.0–4.6 mM in phosphate-buffered saline (PBS).^45^ In contrast, the **SQ-DT** monomer containing the DT group is insoluble in deionized water or PBS (pH 7.4), even at low concentrations (0.1 mM). Thus, a co-assembly strategy was devised to prepare the supramolecular hydrogels containing monomers **SQ** and **SQ-DT** (see the Supporting Information). To obtain the required concentration and volume of the final multicomponent hydrogels **SQ/*x*SQ-DT** (*x*: molar percent of **SQ-DT**), DMSO stock solutions of **SQ** (10.0 mM) and **SQ-DT** (10.0 mM) were first mixed in the appropriate ratio followed by removal of DMSO using a stream of nitrogen or air overnight to yield a dried film. Co-sonication of pre-made **SQ/10SQ-DT** mixed monomers in an ice bath (∼0 °C) resulted in the formation of a clear solution that was then incubated at 37 °C for 15 min and at room temperature overnight, providing transparent hydrogels with a reduced CGC value (0.4–1.0 mM) (Figure S1). Further increasing the molar percentage of **SQ-DT** to 20 mol % resulted in a slightly turbid solution after sonication and later an opaque gel. Hereafter, we use the mixture **SQ/10SQ-DT**, consisting of 10 mol % of **SQ-DT** with the remainder being the native monomer **SQ**, to understand the range by which the hydrogel mechanics can be tuned by light.

### Modulation of the Mechanical Properties of Supramolecular Materials Using UV Light

**Figure 2:**
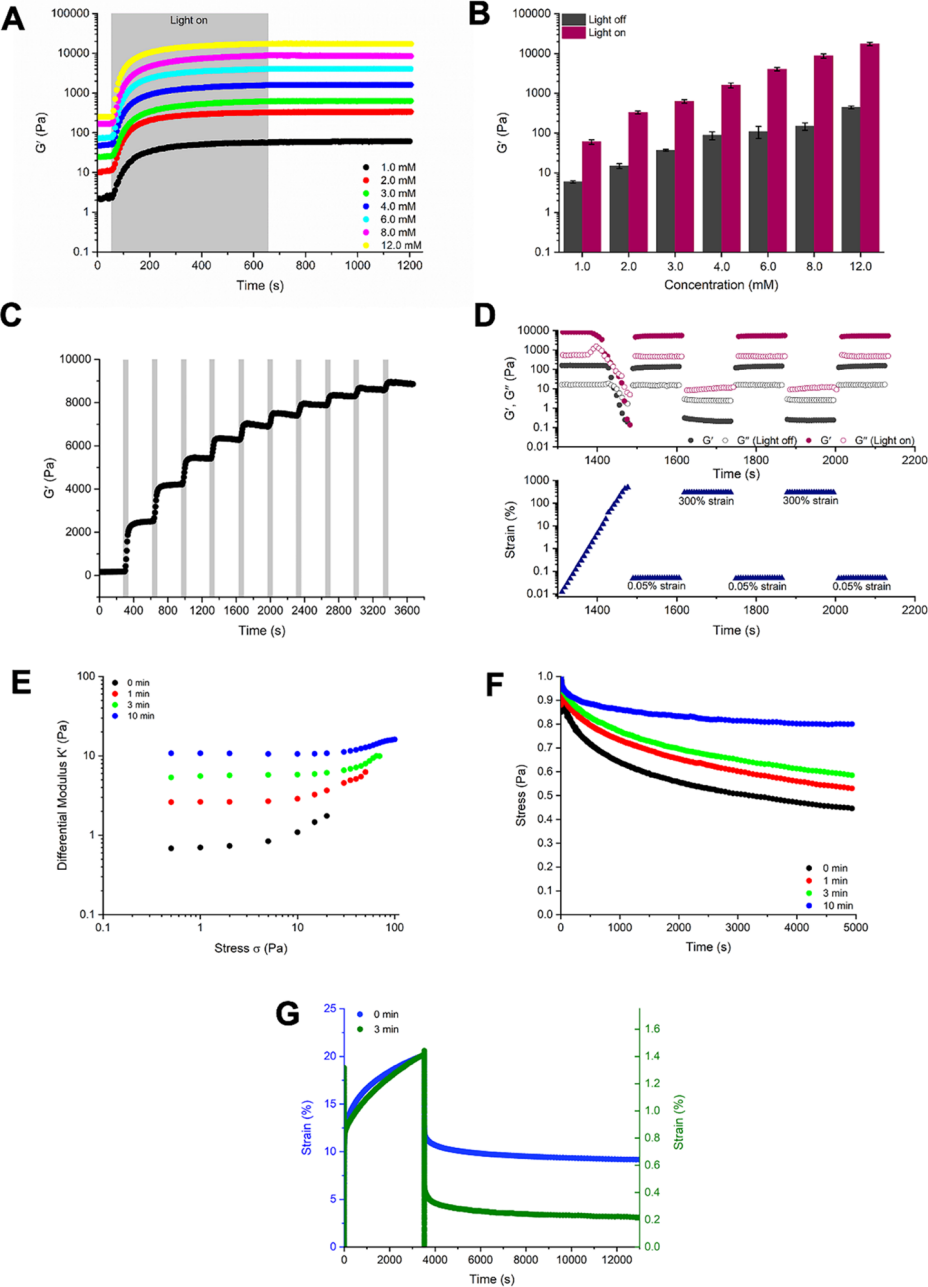
Oscillatory rheological measurements: (A) Averaged (*N* ≥ 3) time sweep measurements of the hydrogel system (**SQ/10SQ-DT**) with varied monomer concentrations using 10 min UV irradiation with fixed strain amplitude (γ = 0.05%) and frequency (*f* = 1.0 Hz) at room temperature. (B) Storage modulus (*G*′) at plateau of **SQ/10SQ-DT** (1.0–12.0 mM) hydrogels with and without 10 min UV irradiation. (C) Time sweep measurements of **SQ/10SQ-DT** (8.0 mM) hydrogels with UV light applied in a stepwise manner at room temperature. (D) Averaged (*N* ≥ 3) strain sweep and step-strain experiments of **SQ/10SQ-DT** (8.0 mM) with and without 10 min UV irradiation. (E) Differential modulus, *K*′, as a function of stress in **SQ/10SQ-DT** (4.0 mM) hydrogels with increasing UV irradiation time (0, 1, 3, and 10 min). (F) Stress relaxation measurements (1% strain) of **SQ/10SQ-DT** (4.0 mM) hydrogels with increasing UV irradiation time (0, 1, 3, and 10 min). (G) Creep and recovery tests of the **SQ/10SQ-DT** (4.0 mM) hydrogels with and without UV irradiation (0 and 3 min). A constant stress of 5 Pa was used during the creep test. Conditions for UV irradiation: ∼10 mW/cm^2^, 320–500 nm filter with maximum absorbance at 365 nm. The shaded areas indicate time periods during which the sample was irradiated by the light source. The error bars were calculated as the standard deviation of repeat measurements (*N* ≥ 3).

The mechanical properties of two-component supramolecular hydrogels in PBS (pH 7.4) with or without light exposure were measured by oscillatory rheology at room temperature. Soft supramolecular hydrogels (**SQ/10SQ-DT**) were prepared through sonication and left to stand overnight resulting in a range of storage moduli (*G*′), from 5.9 ± 0.4 to 440 ± 34 Pa when the total monomer concentration was increased from 1.0 to 12.0 mM (Figure S2). The hydrogels were then further stiffened with the application of UV light (∼10 mW/cm^2^, 320 to 500 nm with a primary peak at 365 nm) remaining optically clear and colorless. The stiffening of the soft hydrogel could be achieved efficiently in less than 10 min and without the use of a photoinitiator. For example, a *G*′ of 17490 ± 1748 Pa was achieved when a total monomer concentration of 12.0 mM with 1.2 mM **SQ-DT** was used (Figure 2A,B). UV irradiation-induced cross-linking of DT is likely responsible for the abrupt increase in hydrogel stiffness; the ring opening of the DT moiety with light in the multicomponent squaramide-based supramolecular materials was confirmed by solid-state NMR experiments against a covalent polymer **PEGdiDT** control (Figures S13 and S14).^54^

The potential to further modulate the mechanical properties of hydrogels (**SQ/10SQ-DT**) by varying UV light intensity and irradiation time was then examined. Increasing the light intensity from ∼5.0 to ∼20.0 mW/cm^2^ decreased the time to reach a plateau in the *G*′ from ∼8 to ∼6 min. All light intensities applied yielded a comparable *G*′ when given enough time (e.g., 10 min) for photo-cross-linking to occur (Figure S4). However, the duration of light exposure was found to affect the final *G*′ value at the same light intensity (10 mW/cm^2^). A small *G*′ value (1255 ± 110 Pa) was observed when only a short irradiation time (e.g., 20 s) was used, whereas a higher final *G*′ value (8704 ± 1045 Pa) was achieved with longer UV irradiation times (e.g., 10 and 30 min) (Figure S5). Most importantly for cell culture applications, this dependence on irradiation time implies that the *G*′ of the supramolecular hydrogel can be controlled in a stepwise manner, opening the door for applications that require spatial or temporal control over hydrogel properties (Figure 2C).

Step-strain experiments were performed to examine the self-recovery properties of the supramolecular polymer networks before and after UV irradiation. At an oscillation frequency of 1 Hz, a high strain amplitude (500%), outside of its linear viscoelastic range, was first applied for 120 s followed by the application of a low strain amplitude (0.05%) for another 120 s, and then the cycle was repeated. The recovery of *G*′ in all the hydrogels tested before UV irradiation was between 80 and 100% of their initial state (Figure S6). After 10 min of UV irradiation, the hydrogels still showed some self-recovery, although to a lesser extent than before UV irradiation (Figure S6). Taking the 8.0 mM **SQ/10SQ-DT** sample as an example, the *G*′ before UV irradiation recovered ∼93% of its initial *G*′ value, while the same hydrogel after 10 min of UV irradiation returned to ∼60% of the total (Figure 2D). Ellman’s assay was performed to estimate the concentration of free thiols that could facilitate a thiol-disulfide exchange at room temperature to gain insight into the reduced self-recovery of the supramolecular hydrogels.^55^ After UV irradiation (5 and 10 min) (Figures S15–S18 and Table S1), an increase in absorbance was recorded at 412 nm for both solutions and hydrogels prepared from the supramolecular polymers, indicating an increase in the concentration of free thiols (from ∼0.0043 to ∼0.44 μM with 10 min UV exposure). These results are in contrast to earlier observations for covalent polymer hydrogel networks^56^ where gels with increased free thiol content showed an increase in self-recovery properties. Because the **SQ-DT** monomer accounts for only 10 mol % of the entire network, the material largely retains its capacity to recover in response to high strain after UV exposure, which is likely due to the physical interactions between filaments.

To compare the mechanical properties of the squaramide-based supramolecular materials to the biopolymer networks that compose the ECM used in cell culture, we characterized their nonlinear elasticity, viscoelasticity, and viscoplasticity. Their capacity to exhibit nonlinear elastic behavior was determined from a pre-stress measurement^57^ in the rheometer to obtain the differential modulus *K*′ = dσ/dγ as a function of stress, σ. At low stresses in **SQ/10SQ-DT** (4.0 mM) prior to UV light exposure, a linear trend in the data was observed with a constant *K*′ equal to the plateau modulus, *G*_0_. Once the critical stress, σ_c_, was reached at 5 Pa, an increase in *K*′ occurred (Figure 2E), as observed in many biopolymer networks.^58^ Irradiation with UV light for 1, 3, and 10 min resulted in an increase in the *G*_0_ and a higher σ_c_, namely, 9.5, 29, and 35 Pa, respectively (Figure 2E and Figures S7 and S8). The decreased sensitivity toward stress for the cross-linked samples is expected, as increased material stiffness is often strongly correlated with a higher onset of nonlinearity. Subsequently, the viscoelasticity of **SQ/10SQ-DT** (4.0 mM) was evaluated by measuring stress relaxation at a strain of 1%. Prior to UV irradiation, the hydrogel relaxed 55% of its initial stress in the measured time range of 5000 s, while after irradiation with UV light for 10 min, the hydrogel relaxed only 20% of the initial stress in the same time frame (Figure 2F). Moreover, the stress relaxation rate of the hydrogel prior to cross-linking falls within the timescales of soft tissues that show more viscoelastic solid character.^22^ As plastic behavior can be observed in viscoelastic materials, the viscoplasticity of **SQ/10SQ-DT** (4.0 mM) was examined using creep and recovery tests (Figure 2G and Figure S9).^20,59^ The degree of plasticity of the gel was found to be 48 ± 7% prior to cross-linking, and decreased to 13 ± 5% after irradiation with UV light for 3 min, indicating that the plastic component of the supramolecular hydrogel decreased substantially with increased cross-link density. These results coincide with previous rheological findings on collagen hydrogels that show slower relaxation rates and decreasing degrees of plasticity with increased cross-linking.^20,60^

We further evaluated the suitability of the supramolecular materials for cell culture experiments by examining the stability and diffusion of macromolecules in the network. First, the stability of the UV-irradiated **SQ/10SQ-DT** (4.0 mM) hydrogel was assessed after incubation at 37 °C with PBS (pH 7.4) or cell culture medium (DMEM containing 20% v/v serum) at different time points. The *G*′ of the PBS or DMEM swollen hydrogels after the same UV exposure (∼10 mW/cm^2^, 10 min) were comparable before and after incubation (e.g., days 1, 3, and 7) (Figure S10). This result indicates that the **SQ-DT** hydrogels remain stable prior to UV irradiation and can be stiffened on-demand during culture. The potential for diffusion of molecules of various molecular weights (e.g., fluoresceinamine Mw = 0.347 kDa, and FITC-dextran Mw ≈ 10 and 70 kDa) through the supramolecular hydrogels was evaluated using fluorescence recovery after photobleaching (FRAP) before and after UV irradiation (5 min). The various molecules were found to diffuse freely within hydrogels of two different stiffnesses at cell culture relevant timescales (Figure S19 and Table S2). Thus, the gels were considered both sufficiently porous and stable for subsequent 3D cell culture experiments.

### Structural Characterization of Supramolecular Materials before and after the Application of UV Light

**Figure 3:**
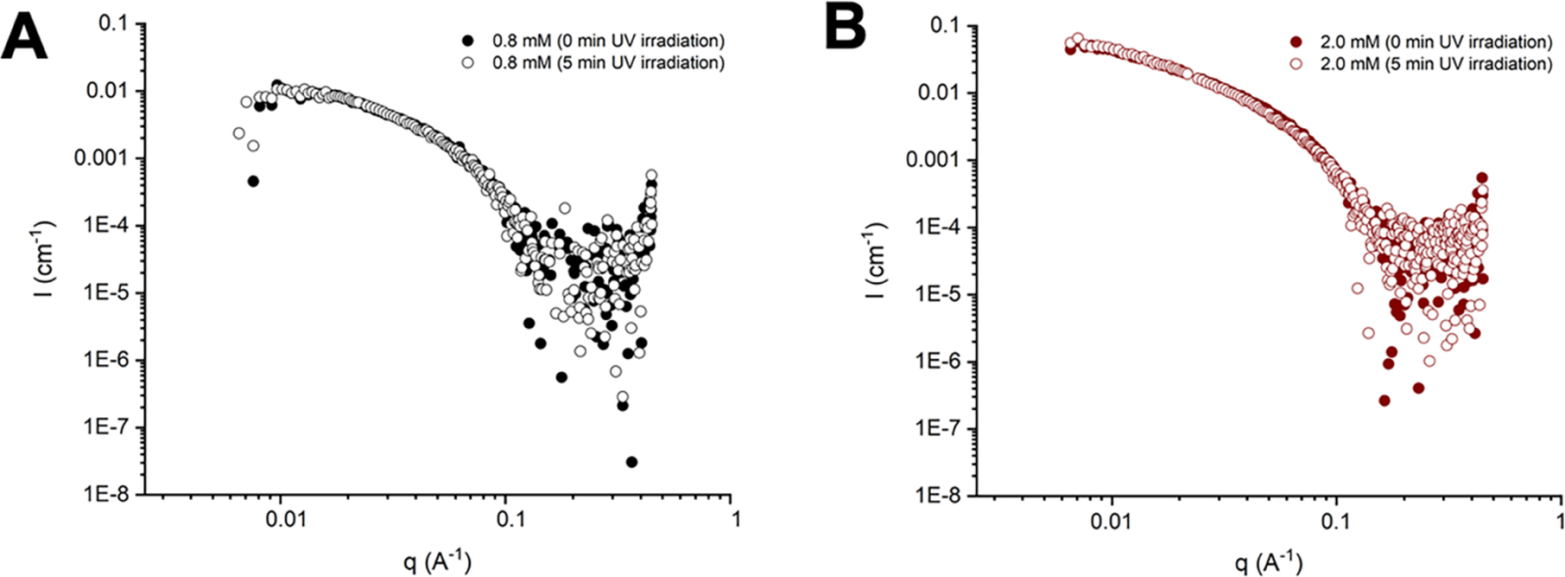
SAXS profiles of different concentrations of supramolecular hydrogels **SQ/10SQ-DT** before (filled circles) and after (empty circles) UV irradiation for 5 min: (A) 0.8 and (B) 2.0 mM.

Cryogenic transmission electron microscopy (cryo-TEM) was performed to image the features of the formed supramolecular networks at the nanoscale. As shown in Figure S20, the imaged supramolecular hydrogels (2.0 mM **SQ/10SQ-DT**) before and after 10 min UV irradiation both presented flexible, entangled filaments at the micrometer scale. However, there was no remarkable change in the filament width under the various conditions: 4.5 ± 0.9 and 3.8 ± 0.8 nm before and after 10 min UV irradiation, respectively (inset figures in Figure S20). To further probe changes within the filaments at smaller length scales in solution and in the gel phase, small-angle X-ray scattering experiments (SAXS) were conducted on the supramolecular materials. The *q*^–1^ slope of the scattering profiles is consistent with the formation of high-aspect-ratio 1D aggregates, and a form factor of flexible cylinders was best fit to the scattering data, yielding a filament radius of ∼2.4 nm (Figure S21). Moreover, on examination of the scattered light intensity across the entire *q*-range (Figure 3) for two different concentrations (e.g., 0.8 and 2.0 mM), insignificant changes to the filamentous aggregates before and after UV irradiation were observed, in line with cryo-TEM imaging. These results show that UV-light-mediated 1,2-dithiolane cross-linking does not perturb the nanoscale structure of the filaments and strongly suggest that the origin of the large changes in rheological properties is largely due to interfilament cross-linking.

### Cell Viability after Light-Mediated Secondary Cross-linking of Squaramide-Based Supramolecular Hydrogels

We previously demonstrated that our soft squaramide-based supramolecular hydrogels consisting of the native **SQ** monomer are cytocompatible, encapsulating several cell types in 3D.^45^ Expanding on this previous work, we herein examine the effect of incorporating DT and applying UV irradiation on cell viability. Three different cell lines were selected to evaluate the cytocompatibility of the supramolecular hydrogel (NIH 3T3 fibroblasts, Hs578T breast cancer cells, and C2C12 myoblasts). Cell suspensions were readily mixed with **SQ-DT** hydrogel (Figure 1B) and then left to incubate followed by staining with calcein AM and PI. Fluorescence microscopy showed that a homogeneous dispersion of the cell suspension is achieved in 3D after seeding (Figure S22A). After 24 h of culture, LIVE/DEAD assays were performed on the cell-laden hydrogels with and without UV irradiation (5 min, 375 nm at ∼10 mW/cm^2^). Without UV irradiation, 92 ± 4% of NIH 3 T3 cells, 93 ± 1% of Hs578T cells, and 89 ± 3% of C2C12 cells were viable (Figure S22B). With 5 min UV irradiation (immediately after seeding) and staining after 24 h culture, the percentage of viable cells decreased slightly to 85 ± 4%–90 ± 2% for all lines tested. Moreover, increasing the concentration of **SQ-DT** resulted in decreased cell viability if UV light was applied immediately after seeding, most notably for the C2C12 cell line (see Figure S23, for 8.0 mM **SQ**, 8.0 mM **SQ/5SQ-DT**, and 8.0 mM **SQ/10SQ-DT**). Longer incubation times (>4 h) of the cells in the materials prior to UV irradiation were found to mitigate the negative effect of high **SQ-DT** concentration. This negative effect is likely due to the higher thiyl radical concentration, which is in line with earlier reports for other radical chemistries.^61−63^ In the experiments below, a sufficiently low concentration (4.0 mM **SQ/10SQ-DT**) was chosen for photo-induced stiffening experiments during cell culture, balancing cell viability and the desired mechanical stiffening of the hydrogel.

### Hs578T Breast Cancer Cells Show Phenotypic Changes Consistent with Secondary Cross-linking of the Supramolecular Materials

Once the cell viability and the various mechanical features of the squaramide-based networks before and after UV irradiation were assayed, the next step was to examine the capacity to remotely modulate material stiffness during 3D cell culture and readout changes in cell behavior. We selected a highly invasive and metastatic breast cancer cell line (Hs578T), because of our interest to use our materials to model the spatiotemporal changes in ECM mechanics in the progression of cancer *in vitro*. Invasive cancer cell types generate sufficient mechanical force through their invadopodia to puncture through the basement membrane independent of proteolysis and thus remodel this matrix in a force-mediated manner.^59^ To enable cells to exert cellular traction on the supramolecular material, we further introduced an additional squaramide monomer bearing an RGD peptide, **SQ-RGD**, into the **SQ/10SQ-DT** hydrogel. Our earlier work showed that the optimal balance between cell adhesion and material stability was found to be at 10 mol % of the RGD peptide,^51^ and based on this result, we opted for this percentage to prepare the multicomponent squaramide hydrogel **SQ/10SQ-DT/10SQ-RGD** for subsequent cell culture assays. The effect of cross-linking the hydrogels on cell behavior was studied in 2D and 3D, comparing cell morphologies before and after UV exposure. Additionally, cross-linking of the materials during culture was performed to evaluate cell behavior in the context of an already established but changing mechanical environment.

**Figure 4:**
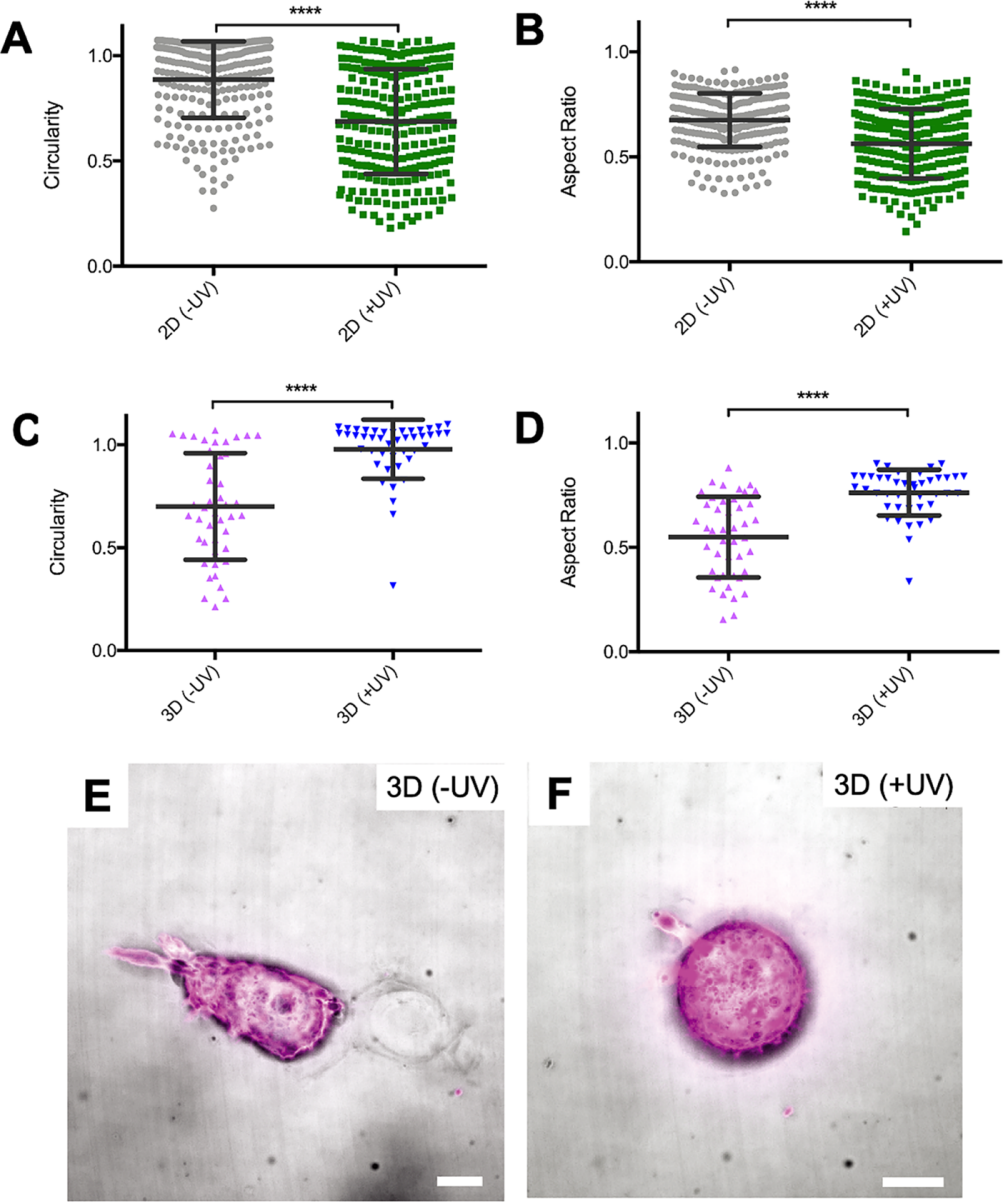
(A)–(D) Quantitative analyses of 2D and 3D culture of mCherry-LifeAct Hs578T cells in **SQ/10SQ-DT/10SQ-RGD** (4.0 mM) hydrogels with different UV irradiation durations (0 and 5 min): (A) cell circularity and (B) aspect ratio after 6 h in 2D culture; (C) cell circularity and (D) aspect ratio after 3 days in 3D culture. The mean and standard deviation are marked within the figures (**** *p* < 0.0001 one-way ANOVA). (E) and (F) Representative high-resolution images of mCherry-LifeAct Hs578T cells after 3 days culture in 3D in **SQ/10SQ-DT/10SQ-RGD** (4.0 mM) hydrogels with different UV irradiation durations: (E) 0 min or (F) 5 min. Scale bar: 10 μm. UV light was applied through a benchtop LED (∼10 mW/cm^2^, 375 nm).

As a first step, mCherry-LifeAct Hs578T breast cancer cells were seeded on top of the **SQ/10SQ-DT/10SQ-RGD** (4.0 mM) hydrogel to study cell morphology in response to material stiffening. The 2D assay facilitates readout of cell behavior in the context of changing material stiffness because interferences from other physical parameters that do play an important role in 3D, such as the micropore architecture and plasticity, are absent.^64,65^ Cells on top of the UV-stiffened network spread significantly more in contrast to those on non-UV-exposed substrates after 6 h (Figure 4A,B, Figures S24 and S25, and Table S3), consistent with previous studies that found the cell area to expand with increased stiffness in 2D.^66^ Furthermore, the cells showed increased front-rear polarity on the UV-irradiated supramolecular hydrogel, a feature critical for tissue formation and directed cell migration,^67^ whereas they maintained a mostly round morphology on the non-UV-irradiated substrate. The increased polarity of cells on UV-exposed hydrogel is reflected by the measured decrease in the averages of both the aspect ratio (from 0.68 ± 0.02 to 0.56 ± 0.02) and circularity (from 0.89 ± 0.02 to 0.68 ± 0.03). The change in cell polarity is a result of the increase in stiffness of **SQ/10SQ-DT** (4.0 mM) hydrogel from 88 ± 11 to 1100 ± 112 Pa (Figure 2B); a Young’s modulus exceeding 1000 Pa was earlier reported to be the effective lower limit to achieve front-rear polarity in 2D.^68,69^

After establishing cell behavior on the supramolecular materials with light-mediated changes in 2D, we moved to their culture in 3D where a strikingly different cell response was observed (Figure 4C–F, Figures S26–S28, Table S4, and Videos 1 and 2). Prior to UV irradiation, the material is soft (∼40 Pa) and highly plastic (48 ± 7%) and the encapsulated cells in 3D culture are elongated, often showing branched protrusions (Figure 4E) with, on average, a low aspect ratio of 0.55 ± 0.059 and a circularity of 0.70 ± 0.079 (Figure 4C,D and Table S4). The morphological features of the cells under this 3D condition are congruent with earlier reports involving materials formed through weak interactions and possessing complex mechanical characteristics.^17,70^ From confocal microscopy images, the cells were observed to remodel the non-proteolytic supramolecular materials by first forming protrusions and then channels (Figure 4E and Figure S28). In sharp contrast, when the materials were exposed to UV light (375 nm) for 5 min, increasing their stiffness (*G*′ ≈ 600 Pa) and decreasing their plasticity (13 ± 5%), cell circularity increased to 0.98 ± 0.044 and the capability of the cells to form protrusions or elongate was observed to diminish (Figure 4F, Figure S28, and Table S4). The reduction of protrusions and thus a more rounded cell shape in 3D has been earlier observed by Chaudhuri and co-workers when the materials’ plasticity was decreased and the stiffness was increased.^20^ The changes in cell morphology due to changing the mechanical environment with UV exposure are inversed for the 2D and 3D assays. In 3D, cell spreading is significantly reduced in a stiffened and less plastic hydrogel (see Figure 4C,D), whereas in 2D, cell spreading is increased with the stiffness of the surface (see Figure 4A,B). The opposite behavior in these two culture formats highlights the need to examine mechanical features of filamentous supramolecular matrices beyond stiffness on moving from 2D to 3D as they are also modified on cross-linking and can impact cell spreading and motility.

**Figure 5:**
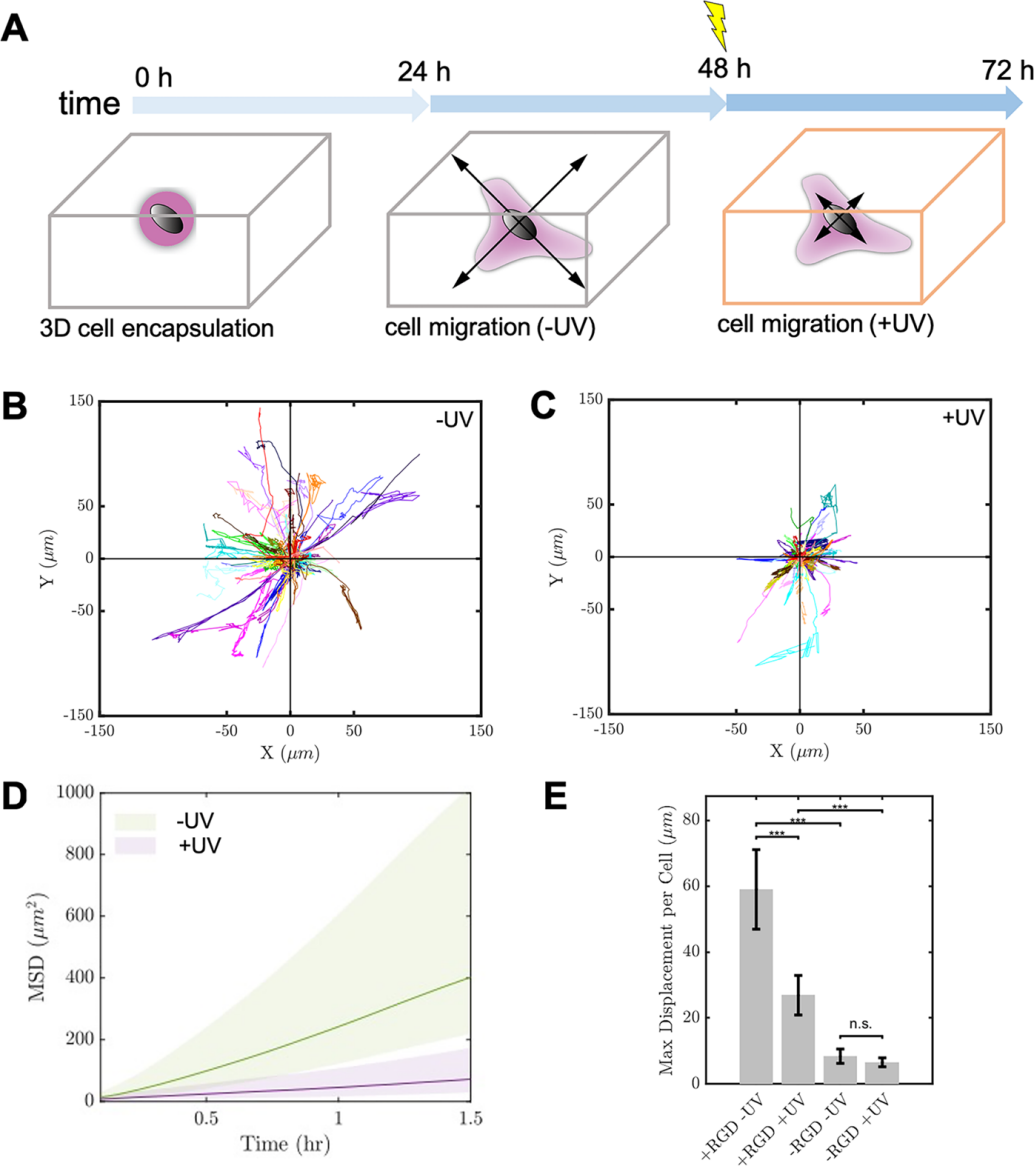
(A) Schematic representation of the UV light application timeline for secondary cross-linking of the supramolecular materials in cell culture experiments (0–72 h). Representations of mCherry-LifeAct Hs578T cell trajectories within multicomponent hydrogels **SQ/10SQ-DT/10SQ-RGD** (4.0 mM) before and after the application of UV light: (B) cells (*N* = 64) traced 24–48 h within hydrogel (0 min UV light) and (C) cells (*N* = 68) traced for 48–72 h after 5 min UV light. (D) Mean-squared displacement (MSD) of the tracked trajectories within the hydrogels (+/– UV light). (E) Effect of the RGD peptide (+/– RGD) and changes in hydrogel stiffness caused by UV light (+/– UV) on the max displacement per cell. UV light was applied using a benchtop LED (∼10 mW/cm^2^, 375 nm).

We examined the potential for the supramolecular material to mimic the temporal changes in mechanical properties that occur in the ECM *in vivo* by cross-linking the supramolecular network with UV light in the middle of a 3-day culture period (Figure 5). Initially the cells were observed to spread, elongate, and migrate through the soft and highly plastic supramolecular gel **SQ/10SQ-DT/10SQ-RGD** (4.0 mM) as evidenced by recorded cell trajectories during confocal time-lapse imaging for 24–48 h (Figure 5B, see Video 3 in the Supporting Information). Prior to photo-cross-linking, the cells were maximally displaced to 59 ± 12 μm with a speed of 21 ± 3.5 μm/h on average. Subsequently, the cell–gel mixture was exposed to UV light mid-culture, and the same positions were imaged for another 24 h to follow changes in phenotype (Figure 5C, time-lapse microscopy for 48–72 h, see Video 4 in the Supporting Information). The cells maintained their spread appearance and polarization, but the capacity of mCherry-LifeAct Hs578T to migrate through the stiffer and less plastic supramolecular hydrogel was impeded, as maximal cell displacement dropped to 27 ± 6.0 μm with an average speed of 15 ± 2.7 μm/h. The mean-squared displacement (MSD), a measure of space explored over time, of the cell center sharply decreases after UV-stiffening (Figure 5D), further confirming that the cells lose their ability to easily migrate through the gel. Although the cells can backtrack through hydrogel channels created in the first 48 h of the experiment (e.g., Figure 4E), the less plastic and stiffened hydrogel after photo-cross-linking constrains their motion primarily along the earlier made tracks. The cells still attempt to form protrusions into the material, but its change in mechanical properties prevents the migration into the unexplored areas of the hydrogel.

To assess the importance of RGD-mediated cell–gel adhesion during cell migration in the material, the photo-cross-linking of the mCherry-LifeAct Hs578T cell-laden hydrogel **SQ10SQ-DT** (4.0 mM) was performed without incorporating the RGD peptide (Figure S29). The cells still tried to form protrusions but lost the ability to manipulate the gel reliably leading to a largely rounded morphology. This finding highlights the importance of incorporating the RGD peptide to target integrin receptors on the cell surface and thereby enable force-mediated remodeling of the supramolecular material. During the 24 h culture in hydrogel lacking RGD and pre-UV irradiation, the Hs578T cells do not displace more than one cell-length (Figure 5E) in the gel on average. However, the cell center-of-mass is in continuous motion as the cells probe their physical environment. When irradiated with UV light at 48 h, the cells in **SQ10SQ-DT** are hindered further (Figure 5E) with protrusions no longer being formed. These results point out that cells encapsulated in a non-proteolytic matrix need a material with specific mechanical characteristics to migrate successfully and efficiently. The physical environment surrounding a cell needs to be below a critical stiffness value and accessible to deformation, for example through integrin binding to RGD peptides. Then, with an adequate degree of plasticity, the cells can successfully invade the material. Overall, we demonstrate that light-mediated cross-linking of the filamentous supramolecular materials can be used to control cell morphology in 2D and 3D and migration in 3D through a decrease in material plasticity.

### Spatial and Temporal Patterning of Bioactive Cues in Supramolecular Materials Containing 1,2-Dithiolanes Using UV Light

**Figure 6:**
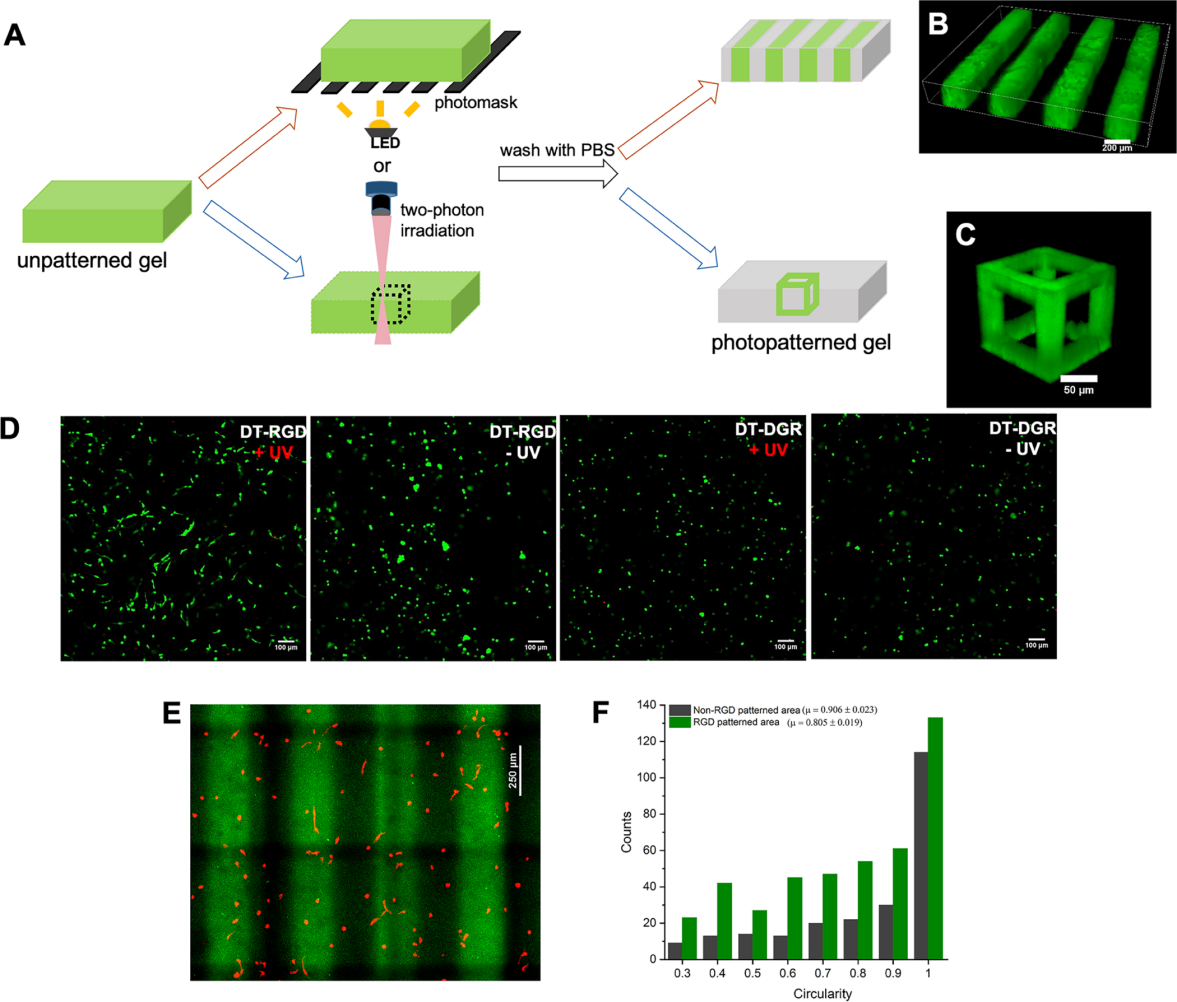
(A) Spatial and temporal patterning of a fluorescent RGD peptide (**(fluorescein)GK(DT)GGGRGDS**) within the **SQ/10SQ-DT** (6.0 mM) hydrogel using a photomask or two-photon laser lithography. (B) 3D confocal fluorescence images of the fluorescent RGD peptide patterned hydrogel using a photomask with 5 min UV light from a benchtop LED (∼10 mW/cm^2^, 375 nm). Scale bar: 200 μm. (C) Confocal microscopy images of a fluorescent hollow cube prepared by two-photon direct laser writing (DLW) using a fluorescent RGD peptide. Scale bar: 50 μm. (D) Confocal images of C2C12 cells after a 3 day culture period in the **SQ/10SQ-DT** (3.0 mM) hydrogel where RGD or DRG peptides were coupled by UV light: **DT-RGD** (0.4 mM) with (i) 5 min and (ii) 0 min UV irradiation; **DT-DGR** (0.4 mM) with (iii) 5 min and (iv) 0 min UV irradiation. Scale bar: 100 μm. C2C12 cells were stained with calcein AM (viable cells, green) and PI (dead cells, red). (E) Representative confocal image (z-stack projection) of encapsulated C2C12 cells after 3 days of culture in the **SQ/10SQ-DT** (3.0 mM) hydrogel patterned with a photomask using 5 min UV irradiation from a benchtop LED. The hydrogel contains **DT-RGD** (0.399 mM) and a fluorescent RGD peptide (**(fluorescein)GK(DT)GGGRGDS**) (0.001 mM) with a total RGD peptide concentration of 0.4 mM. Scale bar: 250 μm. Cells were stained with phalloidin to visualize F-actin, and the green areas indicate the RGD-patterned area. (F) Distribution of circularity of C2C12 cells in the RGD-patterned (green) and unpatterned (black) areas from panel E.

We further explored the possibility for spatiotemporal patterning of bioactive cues in supramolecular materials to mimic the heterogeneity of tissues *in vivo.* Fluorescent (**(fluorescein)GK(DT)GGGRGDS**^54^ and non-fluorescent (**(DT)GGGRGDS** (**DT-RGD**)) RGD peptides with a DT moiety were synthesized to enable photopatterning using either a photomask or direct laser writing (DLW) (Figure 6A). The efficiency of binding of the RGD peptide to **SQ/10SQ-DT** hydrogel by UV irradiation was found to be around 20% for samples of different RGD concentrations (Figure S31), after homogenous UV exposure using a benchtop LED (5 min, ∼10 mW/cm^2^, 375 nm). Spatial patterning of the RGD peptide was then achieved by placing a photomask between the hydrogel and the LED. Using confocal microscopy, the UV-exposed volume of the hydrogel was visualized (Figure 6B) and the concentration of the bound RGD peptide was estimated to be 0.8 μM from a 10 μM concentration of RGD peptide that was initially applied (Figure S32). In addition to benchtop photopatterning, fully 3D-patterned volumes at cell-size relevant length scales were achieved by two-photon direct laser writing into the hydrogel (Figure 6C). The concentration of bound RGD was observed to sharply increase in patterned areas compared with unpatterned areas (Figure S33). This experiment further highlights the potential for spatial and/or temporal control of the network properties by introducing RGD peptides at specific locations and changes to the physical properties of the network in a user-defined manner through the 1,2-dithiolane moiety.

### Photopatterned Cell-Adhesive Peptides in Supramolecular Materials Modulate Cell Morphology

To assess how RGD peptide-patterned supramolecular materials can spatially guide cell behavior, the morphology of C2C12 cells was quantified after 3D encapsulation in hydrogels that were uniformly exposed to UV light and patterned (Figure 6D). Most of the calcein AM-stained C2C12 cells showed a spread morphology with branched protrusions in the **SQ/10SQ-DT** (3.0 mM) hydrogel with the **DT-RGD** peptide (0.4 mM) that was uniformly exposed to UV light. The stiffness of the supramolecular hydrogel in these regions is estimated to be *G*′ = 190 Pa from rheological measurements and is still low enough for changes in cell morphology to be observed in 3D in the materials with RGD. In contrast, cells remained round when encapsulated in the same hydrogel without UV light and thus without access to the RGD peptide, or when the same concentration of a scrambled control peptide **DT-DGR** was used. To investigate cell morphology in a spatially patterned supramolecular hydrogel, **SQ/10SQ-DT** (3.0 mM) was mixed with a 0.4 mM peptide solution of **DT-RGD** and a small amount of **(fluorescein)GK(*DT*)GGGRGDS** (for visualization of the photopatterned regions). C2C12 myoblasts were then seeded in 3D in the **SQ/10SQ-DT/DT-RGD** hydrogel and subsequently photopatterned using a photomask and benchtop LED. Finally, the hydrogel was washed with PBS and DMEM, left to incubate for 3 days, and imaged (Figure 6E). Cell circularity was quantified in both the RGD-patterned (green) and unpatterned (black) areas using a phalloidin stain for actin visualization (Figure 6F and Table S5); C2C12 cells showed an average decrease in circularity from 0.906 ± 0.023 to 0.805 ± 0.019 for RGD-patterned and unpatterned areas in the same hydrogel, respectively. Similar results were obtained for mCherry-LifeAct Hs578T cells (Figure S34 and Table S6). Although the RGD peptide can be introduced into the supramolecular hydrogel network by chemical cross-linking of the **DT-RGD** peptide using UV light, the mechanical properties are also likely simultaneously altered in these regions due to the photoreaction of the DT unit in the gel (see rheology data from Figure S11). Hence, we demonstrate that the light-mediated ligation of **DT-RGD** in the supramolecular hydrogels (**SQ/10SQ-DT**) can be a generic strategy for their post-functionalization with bioactive cues, even during culture.

## Conclusions

We show that a supramolecular monomer end-functionalized with light-activatable 1,2-dithiolane (DT) moieties can provide a handle to engineer the mechanics of supramolecular materials spatiotemporally in 3D cell culture. Exceptionally, the stiffness of the supramolecular hydrogels could be increased to >10 kPa with UV irradiation (365–375 nm) in the absence of a photoinitiator, pointing to the cross-linking of their filaments in the materials. After UV cross-linking, the hydrogels retain clarity and colorlessness that is advantageous for light microscopy and show potential for self-recovery due to the combination of the non-covalent and disulfide-based covalent cross-links present from the co-assembly of both monomers. The soft materials further exhibit nonlinear, viscoelastic, and viscoplastic properties prior to UV irradiation, which are conducive to being manipulated by cells enabling protrusion formation and cell migration when RGD-outfitted monomers are included. These complex mechanical characteristics can be modulated or shut down during culture with UV exposure in a user-defined manner. Importantly, photo-cross-linking reinforces the networks such that the cells can no longer plastically deform the non-proteolytic supramolecular materials by force-mediated remodeling and primarily move along earlier made tracks. Moreover, their mechanical properties can be temporally controlled in a stepwise manner, and bioactive cues, such as the RGD peptide, can be introduced spatially in 3D by photopatterning with a photomask or direct laser writing. Excitingly, this approach opens the door for programmed heterogeneity and shaping at multiple time- and length scales in filamentous supramolecular biomaterials to mimic mechanical phenomena that occur in the ECM during development and disease for *in vitro* studies of cell behavior.

## Supporting Information Available

Full synthetic details for the supramolecular monomers and peptides, hydrogel characterization, 2D/3D cell culture, hydrogel photopatterning (PDF)
Time-lapse microscopy video (0–24 h) from 3D cell culture within a hydrogel prepared with 0 min UV irradiation (MP4)
Time-lapse microscopy video (0–24 h) from 3D cell culture within a hydrogel prepared with 5 min UV irradiation (MP4)
Time-lapse microscopy video (24–48 h) from 3D cell culture within a hydrogel prepared with 0 min UV irradiation (MP4)
Time-lapse microscopy video (48–72 h) from 3D cell culture within a hydrogel prepared with 5 min UV irradiation (MP4)
