# Supplementary material for: Spatial and Temporal Modulation of Cell Instructive Cues in a Filamentous Supramolecular Biomaterial
Source: ACS Appl Mater Interfaces. 2022 Apr 10;14(15):17042–54. doi: 10.1021/acsami.1c24114 (PMC9026256; doi:10.1021/acsami.1c24114)
Supplement: Supplementary file 1 — am1c24114_si_001.pdf [file am1c24114_si_001.pdf]

Supporting Information

Spatial and Temporal Modulation of Cell Instructive Cues in a Filamentous Supramolecular Biomaterial

Ciqing Tong,† Joeri A. J. Wondergem,§ Marijn van den Brink,† Markus C. Kwakernaak,† Ying Chen,† Marco M. R. M. Hendrix,‡ Ilja K. Voets,‡ Erik H. J. Danen,# Sylvia Le Dévédec, # Doris Heinrich, §$ and Roxanne E. Kieltyka†*

Corresponding Author

E-mail: r.e.kieltyka@chem.leidenuniv.nl

S1. Materials and instruments

The chemicals and reagents for the synthesis of squaramide-based tripodal monomers were purchased from commercial suppliers and used without further purification. Deuterated chloroform, methanol and dimethyl sulfoxide were obtained from Euriso-top. Water was deionized prior to use. Dulbecco's phosphate buffered saline (DPBS) was purchased from Sigma Aldrich. Dulbecco's modified Eagle medium (DMEM)-high glucose (D6546) was obtained from Gibco. RPMI-1640 medium was procured from Thermo Fisher Scientific. Calcein AM and propidium iodide (PI) were purchased from Sigma Aldrich. AlexaFluor488-phalloidin antibody was obtained from Invitrogen. μ-slide 4, μ-slide 8 and μ-slide 15 well plates were purchased from Ibidi. Microscopy slides (#1.5, 30 mm) were obtained from Thermo Fisher Scientific. Polydimethylsiloxane (PDMS) (Sylgard 184 Silicon Elastomer Kit, Dow Corning), silicon wafer and fluorosilane (1H, 1H, 2H, 2H-perfluorooctyltrichlorosilane) were purchased from VWR chemicals, Siegert Wafers and Sigma Aldrich, respectively. The monomer **SQ** fluorescent dye- and 1,2-dithiolane-labeled peptide (**(Fluorescein)GK(DT)GGGRGDS**) were synthesized as previously reported.[1, 2] Purification of the monomers **SQ**, **SQ-DT**, **SQ-RGD**, **DT-RGD**, **DT-DGR** and **(Fluorescein)GK(DT)GGGRGDS** were carried out by RP-HPLC on a Vydac C18 reverse- phase column with UV detection. ^{1}H-NMR and ^{13}C-NMR spectra were acquired on a Bruker DMX-400 operating at room temperature. LC-MS data were collected on a Finnigan Surveyor HPLC system with a Gemini reverse-phase C18 column (50 × 4.60 mm) and a UV detection range from 200-600 nm coupled to a Finnigan LCQ Advantage Max mass spectrometer with ESI. The solvent gradient of the mobile phase for LC-MS was CH_3CN-H_2O (10-90%) with 0.1% TFA over 13.5 min. Oscillatory rheology experiments were performed at room temperature on a Discovery Hybrid Rheometer (DHR-2, TA Instruments) using a parallel plate geometry (20 mm diameter). The UV light source on the rheometer was connected to the Excelitas Omnicure S2000 system (λ = 320-500 nm, primary peak: 365 nm) with a light guide (5 mm diameter). Copper grids (300 mesh) with a lacey-carbon support film were obtained from Electron Microscopy Sciences. Plunge-freezing was carried out on a Leica EM GP (Leica Microsystems). Cryo-TEM images of the supramolecular hydrogel samples were acquired on a Tecnai F12 microscope (FEI) equipped with a field emission gun operating at 120 keV using a Gatan UltraScan charge-coupled device (CCD) camera. SAXS experiments were performed on a SAXSLAB GANESHA 300 XL SAXS system equipped with a GeniX 3D Cu ultralow divergence microfocus sealed tube source producing X-rays (λ = 1.54 Å) at a flux of 1×10^{8} ph/s and a Pilatus 300 K silicon pixel detector with 487 × 619 pixels of 172 × 172 μm in size placed at two sample-to-detector distances of 713 and 1513 mm, respectively, in a q-range of $0.009 \leq q \leq 0.456$ Å^{-1}, where $q = 4\pi/\lambda(\sin \theta/2)$. Silver behenate was used to calibrate the beam center and the q-range. The SAXS patterns were brought to an absolute intensity scale and azimuthally averaged to 1D SAXS profiles using the parameters of calibrated detector response function, known sample-to-detector distance, and measured incident and transmitted beam intensities. The SAXS utilities (http://www.sztucki.de/SAXSutilities/) software was used to subtract the scattering contribution of the solvent and quartz cell and to obtain the scattering curves. The SASview software (http://www.sasview.org/) was used to further analyze the obtained SAXS profiles. UV-Vis spectra were collected on a Cary 300 spectrophotometer (Agilent) using a quartz cuvette (path length: 1.0 cm). The photomask for the photopatterning was designed in AutoCad (Autodesk, CA, USA) and printed on premium emulsion film at a resolution of 200k dpi (JD Photo Data, UK). Two-photon crosslinking through direct laser writing (DLW) was performed on the Photonics Professional GT (Nanoscribe GmbH). The fluorescent images of photopatterned hydrogels were acquired on a Nikon Eclipse Ti microscope equipped with a Yokogawa confocal spinning disk unit operated at 10,000 rpm (Nikon, Tokyo, Japan) using a 10x and 20x (CFI Plan Fluor, NA 0.5) objective. The images for the cell viability determination

in 3D cell-laden hydrogels were collected on a Zeiss LSM 710 confocal laser scanning microscope equipped with a Zeiss 5x objective.

S2 Synthetic Procedures

S2.1. Synthesis of monomer SQ-DT

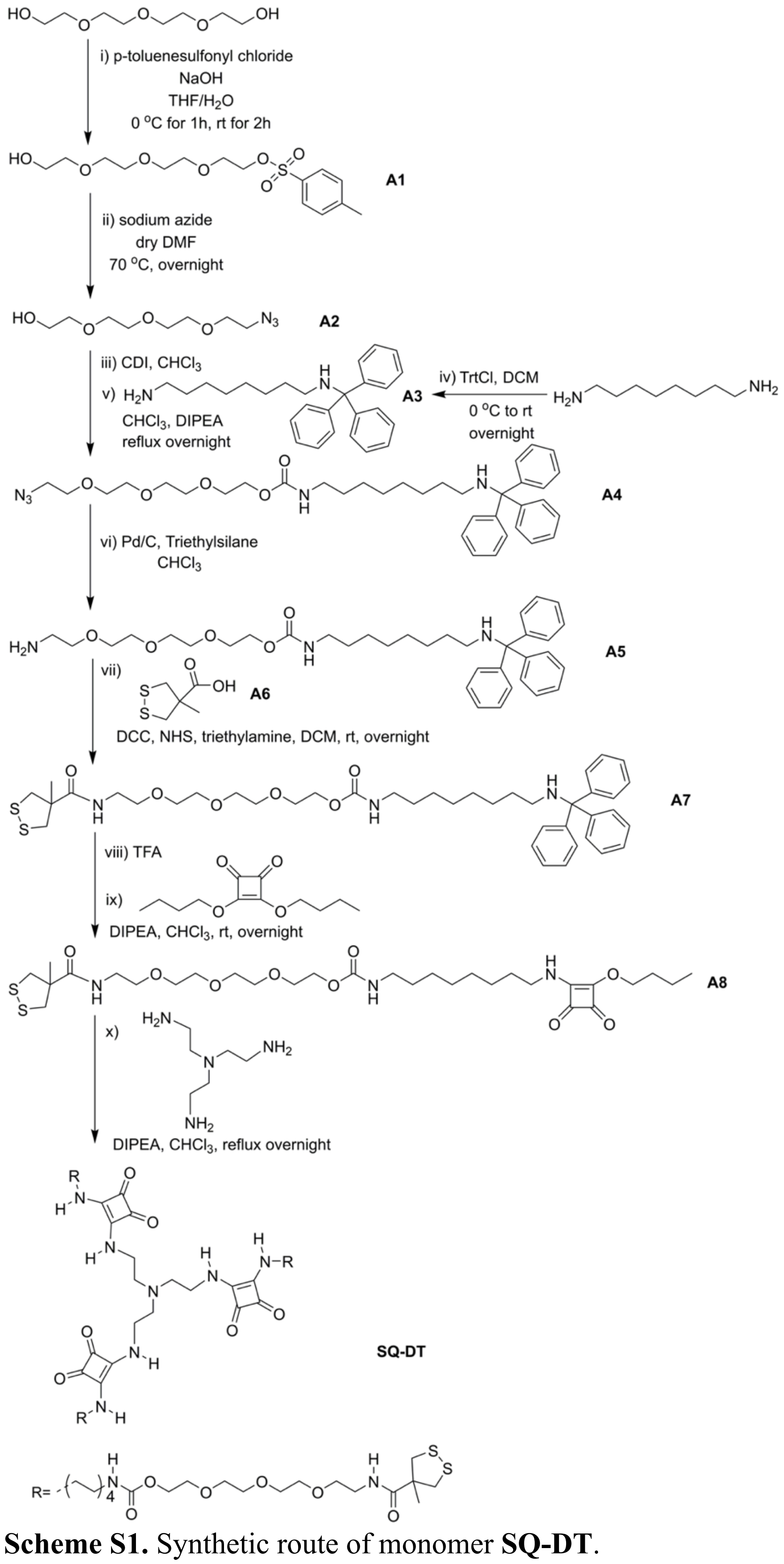

Scheme S1. Synthetic route of monomer **SQ-DT**.

Synthesis of compound A1

Sodium hydroxide (0.99 g, 24.75 mmol) was dissolved in water (6 mL) and added to tetraethylene glycol (29.98 g, 154.36 mmol) in tetrahydrofuran (THF, 6 mL) in an ice bath. Then, a solution of *p*-toluene sulfonyl chloride (2.94 g, 15.44 mmol) in THF (18 mL) was added dropwise over 2 h to the reaction mixture in an ice bath. The reaction was stirred for 4 h at room temperature until complete (as detected by TLC). Dichloromethane (DCM, 100 mL) was added and washed with water (100 mL). The aqueous fraction was extracted with DCM (3 x 100 mL) and the organic fraction was further washed with water (3 x 100 mL). The combined organic fractions were dried with Na_2SO_4, filtered, and the solvent was removed *in vacuo*. The crude product was further purified by silica column chromatography using first petroleum ether (PE)/ethyl acetate (EtOAc) (2:1, v:v) as the eluent and then EtOAc. The product was concentrated by rotary evaporation to obtain an oil-like product **A1**.

Yield: 4.52 g, 84%. ^{1}H-NMR ($CDCl_3$, 400 MHz): 7.74-7.70 (2H, m), 7.29-7.27 (2H, m), 4.10-4.06 (2H, m), 3.64-3.48 (14H, m), 2.85 (1H, s), 2.37 (3H, s). ^{13}C-NMR ($CDCl_3$, 100 MHz): 144.46, 132.43, 129.44, 127.50, 72.06, 70.22, 70.16, 70.05, 69.97, 69.83, 68.91, 68.19, 61.17, 21.20.

Synthesis of compound A2

Sodium azide (3.85 g, 59.22 mmol) was added to a stirred solution of **A1** (4.13 g, 11.85 mmol) in ethanol (34 mL). The reaction mixture was then heated to 70 $^{\circ}$C and stirred overnight. After the reaction was complete (as detected by TLC), the white solid was filtered out and the ethanol was removed *in vacuo*. Water (100 mL) was added to the crude reaction mixture and extracted with DCM (3 x 100 mL). The combined organic fractions were dried with Na_2SO_4, filtered, and the solvent was removed *in vacuo*. The collected oil-like product **A2** was confirmed by NMR and used without further purification.

Yield: 2.44 g, 94%. ^{1}H-NMR ($CDCl_3$, 400 MHz): 3.49-3.43 (12H, m), 3.38-3.35 (2H, m), 3.27 (1H, s), 3.18-3.15 (2H, m). ^{13}C-NMR ($CDCl_3$, 100 MHz): 72.07, 70.10, 70.08, 70.05, 70.01, 69.98, 69.73, 69.47, 60.92, 50.06.

Synthesis of compound A3

1,8-Diaminooctane (16.0 g, 110.91 mmol) was first dissolved in DCM (200 mL). A solution of trityl chloride (7.73 g, 27.73 mmol) in DCM (100 mL) was then added dropwise to the 1,8-diaminooctane solution in an ice bath over 2 h, followed by stirring at room temperature overnight. Once the reaction was complete (as determined by TLC), the DCM was removed by rotary evaporation. EtOAc (100 mL) was then added and the fraction was washed with water (100 mL). The aqueous fraction was back-extracted with EtOAc (2 x 100 mL). The organic fractions were combined, dried with Na_2SO_4, and the solvent was removed *in vacuo*. The crude product was further purified by silica column chromatography using a solvent gradient of DCM to DCM/MeOH/NH_4OH (20:1:0.1, v:v:v). The product was evaporated by rotary evaporation to obtain a yellow oil-like product **A3**.

Yield: 5.62 g, 52 %. ^{1}H-NMR ($CDCl_3$, 400 MHz): 7.51-7.49 (6H, m), 7.30-7.28 (6H, m), 7.26-7.16 (3H, m), 2.69-2.66 (2H, m), 2.14-2.11 (2H, m), 1.47-1.25 (12H, m). ^{13}C-NMR ($CDCl_3$, 100 MHz): 146.42, 128.72, 127.79, 126.19, 70.90, 43.61, 42.26, 33.77, 30.94, 29.68, 29.51, 27.38, 26.92.

Synthesis of compound A4

1,1-Carbonyldiimidazole (1.33 g, 8.20 mmol) was added to oil-like compound **A2** (1.63 g, 7.45 mmol) and stirred at room temperature without additional solvent. After 30 minutes, chloroform (2 mL) was added to the reaction mixture and left to stir for another 1 h at room temperature. After the reaction was complete (as determined by TLC), more chloroform (15 mL) was added to the crude, followed by the addition of compound **A3** (3.46 g, 8.95 mmol) and N,N-diisopropylethylamine (DIPEA, 1.94 mL, 11.17 mmol). The crude was refluxed overnight and completion of the reaction was determined by TLC analysis. The crude was then transferred to a separatory funnel and diluted with DCM (50 mL). The organic fraction was washed with water (50 mL) and the water fraction was collected. The aqueous fraction was then back extracted with DCM (2 x 50 mL). The combined organic fractions were dried with Na_2SO_4, filtered, and the solvent was evaporated *in vacuo*. The crude product was purified by silica column chromatography using PE/EtOAc solvent gradient (4:1 to 1:1, v:v). The product was evaporated by rotary evaporation to obtain the yellow oil-like product **A4**.

Yield: 3.57 g, 76%. ^{1}H-NMR ($CDCl_3$, 400 MHz): 7.61-7.58 (m, 6H), 7.38-7.34 (m, 6H), 7.28-7.24 (m, 3H), 5.10-5.07 (m, 1H), 4.33-4.30 (m, 2H), 3.78-3.72 (m, 12H), 3.44-3.41 (m, 2H), 3.26-3.21 (m, 2H), 2.24-2.21 (m, 2H), 1.60-1.15 (m, 12H). ^{13}C-NMR ($CDCl_3$, 100 MHz): 156.23, 146.16, 128.44, 127.54, 125.94, 70.65, 70.47, 70.44, 70.30, 69.85, 69.46, 63.57, 50.41, 43.34, 40.79, 30.64, 29.74, 29.34, 29.04, 27.07, 26.52.

Synthesis of compound A5

A4 (2.2 g, 3.48 mmol) was dissolved in dry methanol (5 mL) and dry DCM (5 mL). Palladium on carbon (39.38 mg, 0.38 mmol) was added to the clear stirring solution above. The reaction was sealed with a rubber septum and flushed with a stream of nitrogen gas over 30 minutes. Then, triethylsilane (6 mL, 37.56 mmol) was added slowly to the stirring solution over the course of 30 min. The reaction mixture was left to stir for another 3 hours at room temperature. After the reaction was complete (as determined by TLC), the mixture was filtered over celite to remove the palladium on carbon. The solvent was concentrated by rotary evaporation and further dried by a gentle stream of air. The product **A5** was directly used without further purification.

Synthesis of compound A6

Compound **A6** was synthesized according to as previously reported.[3] Briefly, 3,3′-dichloropivalic acid (4.00 g, 23.38 mmol) was added to a 2-neck round bottom flask and water (40 mL) was added. To the stirred suspension, sodium carbonate (2.30 g, 21.71 mmol) was slowly added, followed by the dropwise addition of potassium thioacetate (5.34 g, 46.78 mmol) in water (5 mL) over 10 minutes at room temperature. The resulting clear solution was heated to 100 °C and refluxed for 8 hours. Then, more sodium carbonate (7.44 g, 70.16 mmol) was added to the reaction and further refluxed at 100 °C. After the disappearance of the starting material, dimethyl sulfoxide (DMSO, 3.6 mL) was added, followed by refluxing for another 4 hours. The basic solution (pH~9) was allowed to cool to room temperature and then acidified (pH~1) with hydrochloric acid (36.5-38.0%) yielding a yellow precipitate. The precipitate was filtered off *in vacuo* and washed with ice cold water 3 times. The yellow solid was left to dry *in vacuo* at room temperature overnight.

Yield: 2.93 g, 75%. ^{1}H-NMR ($CDCl_3$, 400 MHz): 3.71-3.68 (2H, d), 2.97-2.94 (2H, d), 1.54 (3H, s). ^{13}C-NMR ($CDCl_3$, 100 MHz): 181.37, 57.52, 47.72, 24.12.

Synthesis of compound A7

A6 (0.68 g, 4.15 mmol) was dissolved in dry DCM (10 mL) and stirred in an ice bath. Then, N-hydroxysuccinimide (NHS, 0.72 g, 6.22 mmol) and N,N′-dicyclohexylcarbodiimide (DCC, 1.20 g, 5.81 mmol) were slowly added to the stirring solution. The crude was allowed to warm to room temperature and left to stir for another hour. The oil-like product **A5** (2.10 g, 3.48 mmol) was re-dissolved in dry DCM (10 mL) and added to the stirring solution together with triethylamine (1.15 mL, 8.30 mmol). The crude was left to stir overnight at room temperature. After the reaction was complete (as determined by TLC), the reaction mixture was transferred to a separatory funnel and diluted with water (50 mL) and DCM (50 mL). The aqueous fraction was extracted with DCM (2 x 50 mL). The combined organic layer was dried with Na_2SO_4. The Na_2SO_4 was filtered off and the solvent was removed *in vacuo*. The crude product was further purified by silica column chromatography using PE/EtOAc solvent gradient (4:1 to 1:1, v:v). The yellow oil-like product **A7** was obtained by evaporating the solvent *in vacuo*.

Yield: 1.80 g, 69%. ^{1}H-NMR ($CDCl_3$, 400 MHz): 7.52-7.49 (m, 6H), 7.30-7.26 (m, 6H), 7.20-7.16 (m, 3H), 6.74 (br s, 1H), 5.20 (br s, 1H), 4.24-4.22 (m, 2H), 3.70-3.47 (m, 16H), 3.18-3.13 (m, 2H), 3.01-2.98 (m, 2H), 2.30 (br s, 1H), 2.16-2.12 (m, 2H), 1.49 (s, 3H), 1.35-1.25 (m, 12H). ^{13}C-NMR ($CDCl_3$, 100 MHz): 174.71, 156.58, 146.49, 129.17, 127.86, 126.27, 70.98, 70.75, 70.68, 70.62, 70.36, 69.86, 69.70, 63.85, 57.13, 49.45, 43.68, 41.19, 39.92, 30.98, 30.08, 29.68, 29.39, 27.44, 26.88, 24.12.

Synthesis of compound A8

Compound **A7** (1.22 g, 1.62 mmol) was dissolved in trifluoroacetic acid (TFA, 15 mL) and was left to stir for 3 h at room temperature. Completion of the reaction was confirmed by TLC analysis and the remaining TFA was evaporated by a gentle stream of air. The crude was re-dissolved in chloroform (20 mL), followed by the addition of 3,4-dibutoxy-3-cyclobutene-1,2-dione (420 μL, 1.94 mmol) and DIPEA (564 μL, 3.24 mmol). The mixture solution was left to stir overnight at room temperature. The reaction mixture was transferred to a separatory funnel after confirming its completion using LC-MS and diluted with water (50 mL) and DCM (50 mL). The aqueous fraction was extracted with DCM (2 x 50 mL) and the combined organic layers were dried with Na_2SO_4. The Na_2SO_4 was filtered off and the solvent was removed *in vacuo*. The crude product was further purified by silica column chromatography using a solvent gradient from PE/EtOAc (2:1, v:v) to pure EtOAc. The oil-like product **A8** was obtained by evaporating the solvent *in vacuo*.

Yield: 0.72 g, 67%. ^{1}H-NMR ($CDCl_3$, 400 MHz): 7.52-7.48 (m, 1H), 6.78-6.75 (m, 1H), 5.25-5.17 (m, 1H), 4.56-4.46 (m, 2H), 4.03-3.93 (m, 2H), 3.49-3.37 (m, 14H), 3.29-3.21 (m, 4H), 2.97-2.92 (m, 2H), 2.82-2.79 (m, 2H), 1.64-1.05 (m, 19H), 0.81-0.74 (m, 3H). ^{13}C-NMR ($CDCl_3$, 100 MHz): 189.03, 182.44, 176.74, 174.24, 156.17, 72.84, 70.12, 70.05, 69.76, 69.21, 69.04, 63.33, 60.00, 56.73, 48.62, 44.43, 44.24, 40.57, 39.37, 31.60, 30.19, 29.45, 28.70, 28.64, 26.23, 23.69, 18.26, 13.19. LC-MS: t = 7.41min, m/z: 662.27 $[M+H]^+$, calcd: 661.31.

Synthesis of compound SQ-DT

Component **A8** (0.41 g, 0.63 mmol) was dissolved in chloroform (20 mL) to which tris(2-aminoethyl)amine (28 μL, 0.19 mmol) and DIPEA (110 μL, 0.63 mmol) were added and left to reflux overnight. After the reaction was complete (as determined by LC-MS), the crude was purified by silica column chromatography using a gradient from pure EtOAc to EtOAc/MeOH (4:1, v:v), and then by HPLC with UV detection and lyophilized to obtain a white solid **SQ-DT**.

Yield: 0.15 g, 42%. ^{1}H-NMR (DMSO-d_6, 400 MHz): 7.93-7.91 (m, 3H), 7.48 -7.27 (m, 6H), 7.19-7.15 (m, 3H), 4.04-4.01 (m, 6H), 3.56-3.40 (m, 52H), 3.26-3.20 (m, 8H), 3.00-2.90 (m, 12H), 2.72-2.69 (m, 6H), 1.49-1.21 (m, 45H). ^{13}C-NMR (DMSO-d_6, 100 MHz): 182.85, 173.63, 167.94, 156.57, 70.20, 70.08, 69.37, 69.18, 63.42, 57.57, 47.66, 43.76, 31.18, 29.85, 29.15, 26.69, 26.32, 24.05, 23.93. LC-MS: t = 6.06 min, m/z: 1909.42 $[M+H]^+$. HR-MS (ESI) m/z calcd. $C_{84}H_{141}N_{13}O_{24}S_6$ 1907.85, found: 954.9341 $[M+2H]^{2+}/2$.

S2.2. Synthesis of monomer SQ-RGD

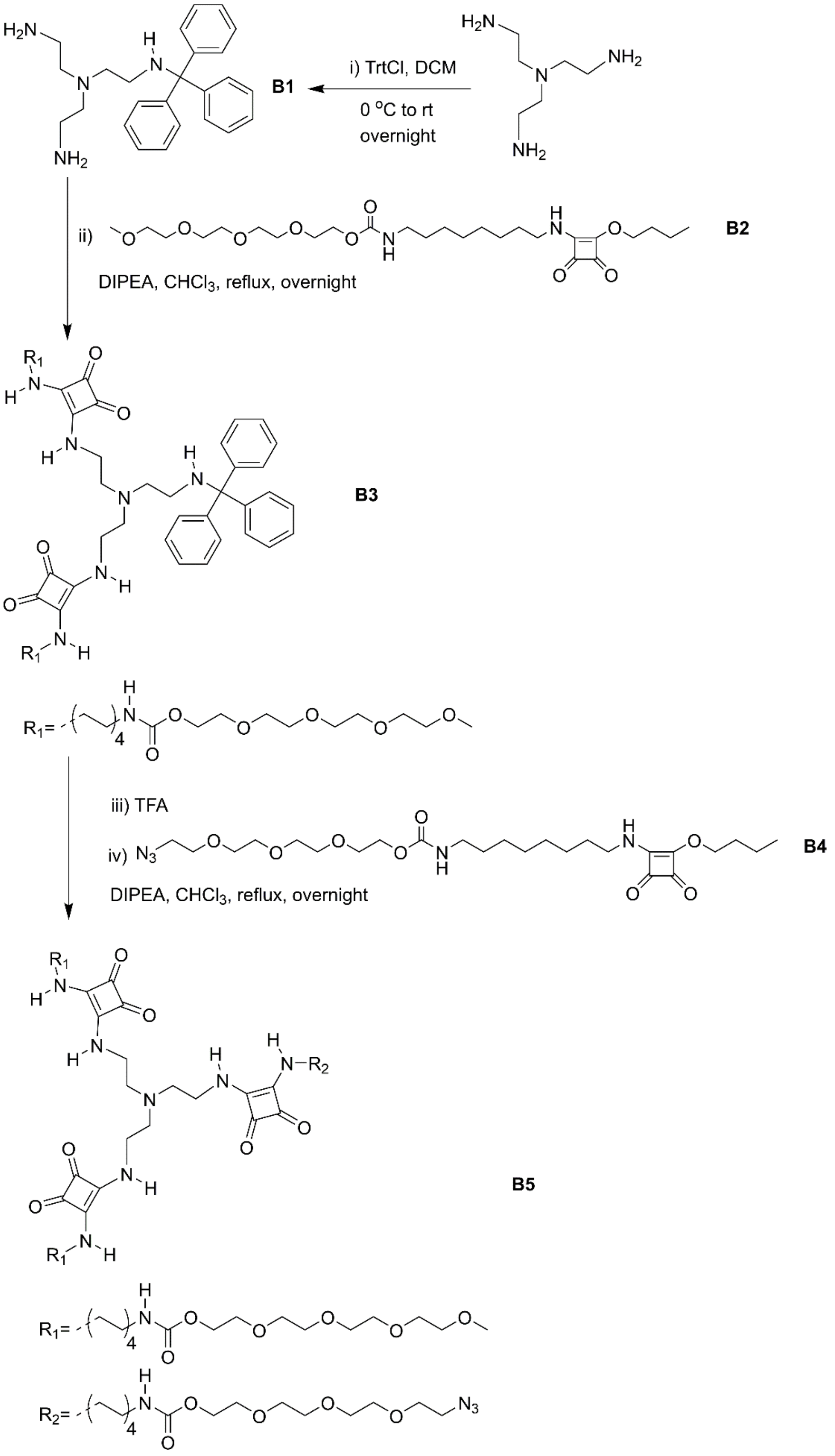

Scheme S2. Synthetic route of monomer **SQ-N_3** (**B5**).

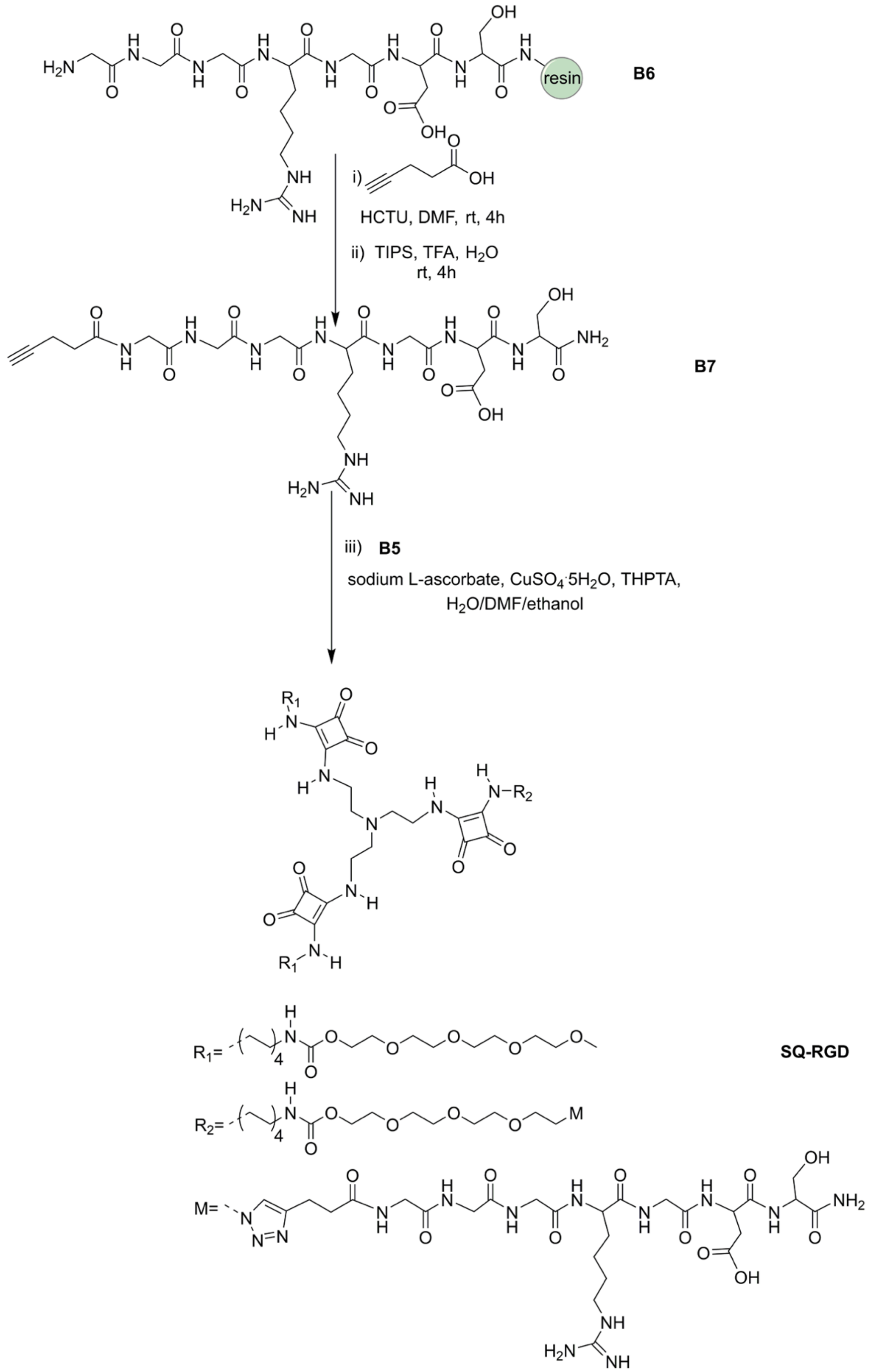

Scheme S3. Synthetic route of monomer **SQ-RGD**.

Synthesis of compound B1

Tris(2-aminoethyl)amine (5 mL, 33.37 mmol) was dissolved in DCM (200 mL) and stirred in an ice bath. Trityl chloride (0.93 g, 3.34 mmol) in DCM (100 mL) was added dropwise to the stirring solution over 2 hours and furthered stirred overnight at room temperature. After the reaction was complete (as determined by TLC), the solution was concentrated by rotary evaporation and EtOAc (100 mL) was added. The crude was washed with water (3 x 100 mL) and the aqueous layers were discarded. The combined organic layer was dried over Na_2SO_4 and the solvent was removed under vacuum to obtain the colorless oil-like product **B1** without further purification.

Yield: 1.23 g, 95 %. ^{1}H-NMR ($CDCl_3$, 400 MHz): 7.55-7.51 (m, 6H), 7.49-7.28 (m, 6H), 7.25-7.19 (m, 3H), 2.72-2.60 (m, 4H), 2.44-2.26 (m, 2H), 2.38-2.35 (m, 4H), 2.29-2.26 (m, 2H). ^{13}C-NMR ($CDCl_3$, 100 MHz): 146.57, 128.94, 128.16, 126.59, 70.98, 58.11, 55.36, 41.27, 40.37.

Synthesis of compound B3

Component **B2** (1.21 g, 2.28 mmol) was synthesized according as previously reported,[1] and dissolved in chloroform (20 mL), followed by the addition of **B1** (402 mg, 1.03 mmol) and DIPEA (397 μL, 2.28 mmol). The reaction mixture was refluxed overnight. After the reaction was complete (as determined by LC-MS), the mixture was transferred to a separatory funnel and diluted with DCM (100 mL) and water (100 mL). The organic layer was collected, and the aqueous layer was further extracted with DCM (2 x 100 mL). The combined organic layers were dried with Na_2SO_4. The Na_2SO_4 was filtered, and the solvent was removed by rotary evaporation *in vacuo*. The obtained crude was purified by silica column chromatography using a gradient from pure EtOAc to EtOAc/MeOH (4:1, v:v). The solvent was evaporated *in vacuo* to provide the oil-like product **B3**.

Yield: 0.65 g, 49%. ^{1}H-NMR ($CDCl_3$, 400 MHz): 7.44-7.40 (m, 6H), 7.34-7.27 (m, 6H), 7.24-7.11 (m, 3H), 5.05-5.01 (m, 2H), 4.23-4.20 (m, 4H), 3.71-3.55 (m, 36H), 3.39 (s, 6H), 3.21-3.10 (m, 4H), 2.60-2.42 (m, 6H), 2.23-2.20 (m, 2H), 1.68-1.28 (m, 24H). ^{13}C-NMR ($CDCl_3$, 100 MHz): 182.90, 172.52, 146.12, 128.47, 127.78, 126.15, 71.89, 70.39, 69.65, 69.29, 63.76, 59.01, 44.75, 40.97, 30.93, 29.89, 29.15, 26.69, 26.46.

Synthesis of compound B4

Compound **A4** (1.02 g, 1.64 mmol) was dissolved in TFA (10 mL) and left to stir for 3 h at room temperature. Completion of the reaction was confirmed by TLC and the TFA was then removed by a gentle stream of air. The crude was re-dissolved in chloroform (30 mL), followed by the addition of 3,4-dibutoxy-3-cyclobutene-1,2-dione (425 μL, 1.97 mmol) and DIPEA (571 μL, 3.28 mmol). The reaction mixture was left to stir overnight at room temperature. After the reaction was complete (as determined by TLC), the mixture was transferred to a separatory funnel and diluted with water (100 mL) and DCM (100 mL). The aqueous fraction was extracted with DCM (2 x 100 mL). The combined organic layers were dried with Na_2SO_4. The Na_2SO_4 was filtered off and the solvent was removed *in vacuo*. The crude product was further purified by silica column chromatography using a gradient from PE/EtOAc (2:1, v:v) to pure EtOAc. The oil-like product **B4** was obtained by evaporating the solvent *in vacuo*.

Yield: 0.54 g, 61%. ^{1}H-NMR ($CDCl_3$, 400 MHz): 7.17-7.13 (m, 1H), 4.93-4.90 (m, 1H), 4.68-4.61 (m, 2H), 4.20-4.15 (m, 2H), 3.65-3.62 (m, 12H), 3.40-3.33 (m, 2H), 3.13-3.08 (m, 2H), 1.76-1.26 (m, 18H), 0.94-0.91 (m, 3H). ^{13}C-NMR ($CDCl_3$, 100 MHz): 189.70, 182.69, 177.54, 172.93, 172.43, 156.50, 73.44, 70.67, 70.64, 70.61, 70.50, 70.04, 69.65, 64.44, 63.85, 50.68, 44.87, 41.64, 40.96, 32.01, 30.98, 30.57, 29.85, 29.06, 28.99, 26.58, 26.28, 18.65, 13.68.

Synthesis of compound SQ-N_3 (B5)

Compound **B3** (0.51 g, 0.39 mmol) was dissolved in TFA (10 mL) and left to stir for 2 h at room temperature. Completion of the reaction was confirmed by TLC and the remaining TFA was removed by a gentle stream of air. The crude was re-dissolved in chloroform (30 mL), followed by the addition of **B4** (0.25 g, 0.46 mmol) and DIPEA (135 µL, 0.78 mmol). The reaction mixture was left to reflux overnight. After the reaction was complete (as detected by LC-MS), the mixture was transferred to a separatory funnel and diluted with water (100 mL) and DCM (100 mL). The aqueous fraction was extracted with DCM (2 x 100 mL) and the combined organic layers were dried with Na_2SO_4 and filtered. The solvent was removed *in vacuo* and the crude product was purified by silica column chromatography using a solvent gradient from pure EtOAc to EtOAc/MeOH (4:1, v:v). The product was further purified by reverse phase silica column chromatography on a C18 silica gel column using a gradient of CH_3CN/H_2O (1:9 to 9:1, v:v) over 41 minutes. The product was lyophilized overnight to obtain the white solid **SQ-N_3** (**B5**).

Yield: 0.24 g, 40%. ^{1}H-NMR ($CDCl_3$, 400 MHz): 7.46 (br s, 2H), 7.28 (br s, 2H), 7.16 (br s, 2H), 4.04-4.00 (m, 6H), 3.60-3.37 (m, 52H), 3.22 (s, 6H), 2.95-2.90 (m, 6H), 2.70-2.68 (m, 6H), 1.50-1.21 (m, 38H). ^{13}C-NMR ($CDCl_3$, 100 MHz): 181.75, 181.54, 167.38, 166.83, 155.47, 70.61, 69.15, 69.11, 69.06, 69.03, 68.92, 68.60, 68.24, 62.32, 57.38, 54.07, 49.33, 42.64, 40.90, 39,52, 30.05, 28.72, 28.03, 27.96, 25.56, 25.19. LC-MS: t = 5.49 min, m/z: 1527.67 $[M+H]^+$. HR-MS (ESI) m/z calcd. $C_{71}H_{123}N_{13}O_{23}$: 1525.89, found: 763.9499 $[M+2H]^{2+}/2$.

Synthesis of peptide RGD and DGR

The peptides **RGD** (**GGGRGDS**) and **DGR** (**GGGDGRS**) were synthesized by standard Fmoc solid-phase peptide synthesis (SPPS) on a Rink amide resin at room temperature. Using the synthesis of the **RGD** peptide **GGGRGDS** (**B6**, 0.5 mmol) as an example: the resin (0.68 g, 0.74 mmol/g) was first swollen in N,N-dimethylformamide (DMF, 8 mL) for 30 min followed by deprotection of the Fmoc groups of the resin using piperidine in DMF (20%, v/v, 8 mL) for 1 h with shaking. The resin was washed with DMF (5 x 8 mL) and then functionalized with the first Fmoc-protected amino acid (2.0 eq.) by shaking for 4 h with HCTU (3.0 eq.), and DIPEA (5.0 eq.) in DMF (8 mL). Then, the resin was washed with DMF (5 x 8 mL), followed by deprotection of the Fmoc group of the amino acid with piperidine in DMF (20%, v/v, 8 mL) by shaking for another 30 min. Subsequent amino acid couplings were performed according to the same protocol. Once the coupling of the last Fmoc-amino acid was finished, the peptide was deprotected using piperidine in DMF (20%, v/v, 8 mL) with 30 min shaking and further washed with DMF (5 x 8 mL). The peptide was cleaved from the resin using a mixture of TFA:TIPS:water (95:2.5:2.5, v:v:v, 1 mL) with 4 h shaking at room temperature. Then, the crude peptide was precipitated from cold diethyl ether, collected by centrifugation, and re-dissolved in water. The peptide **B6** was detected and confirmed by LC-MS. The remaining **RGD** peptide on resin (**GGGRGDS**) was stored at -20 °C before use.

A control peptide sequence **DGR** (**GGGDGRS**) that is unable to bind to cell surface integrins was synthesized according to the same procedure as the **RGD** peptide described above.

Synthesis of alkyne-functionalized RGD peptide B7

The **GGGRGDS** (**B6**) peptide on resin (0.25 mmol) was further functionalized with 4-pentynoic acid (2.0 eq.) by shaking with HCTU (3.0 eq.) and DIPEA (5.0 eq.) in DMF (8 mL) for 6 h. After confirming the reaction was complete by LC-MS, the peptide was then cleaved from the resin using TFA:TIPS:water (95:2.5:2.5, v:v:v, 8 mL) with 6 h shaking. The peptide was precipitated using cold diethyl ether, collected by centrifugation and re-dissolved in water.

B7 was confirmed by LC-MS and without any other further purification was then lyophilized overnight. The obtained peptide was stored at -20 °C before use. LC-MS: t = 1.02 min, m/z calcd: 683.30, found: 684.28 $[M+H]^+$.

Synthesis of compound SQ-RGD

The alkyne-functionalized peptide **B7** was coupled to azide-based compound **B5** through copper(I)-catalyzed azide alkyne cycloaddition (CuAAc). Firstly, sodium L-ascorbate (52.69 mg, 265.98 μmol) and copper(II) sulfate pentahydrate (8.85 mg, 35.46 μmol) were dissolved separately in H_2O (139 μL) by vortexing. Then, a homogeneous bright yellow solution was obtained by mixing the above two solutions with 60 s vortexing. Tris(3-hydroxypropyltriazolylmethyl) amine (THPTA, 7.7 mg, 17.73 μmol) dissolved in ethanol (280 μL), and compound **B5** (67.64 mg, 44.33 μmol) dissolved in DMF (1.7 mL) were mixed in a 10 mL flask, followed by the addition of the yellow solution above. Finally, peptide **B7** (56.42 mg, 82.49 μmol) dissolved in DMF (320 μL) and H_2O (139 μL) was added into the reaction mixture and stirred at room temperature for 4h under N_2 gas. After the reaction was complete as determined by LC-MS, the crude was first dialyzed against water (cut off Mw 500-1000 Da) for two days at room temperature and further purified by HPLC. The white powder product **SQ-RGD** was obtained through overnight lyophilization and stored at -20 °C before use. HR-MS (ESI) m/z calcd. $C_{97}H_{164}N_{24}O_{34}$: 2209.18, found: 737.7365 $[M+3H]^{3+}/3$.

S2.3. Synthesis of 1,2-dithiolane outfitted peptides (DT-RGD and DT-DGR)

To obtain RGD-peptides end-functionalized with the 1,2-dithiolane group, e.g. **DT-RGD** (**(DT)GGGRGDS**) and **DT-DGR** (**(DT)GGGDGRS**), the peptides **RGD** and **DGR** on resin were separately reacted with 1,2-dithiolane **A6** (2.0 eq.) by shaking in combination with HCTU (3.0 eq.) and DIPEA (5.0 eq.) in DMF (8 mL) overnight. After reaction, the peptide-containing resin was washed with DMF (5 x 8 mL), and cleaved with TFA:TIPS:water (95:2.5:2.5, v:v:v, 8 mL) over 4 h with shaking. The crude peptide was precipitated using cold diethyl ether, collected by centrifugation and re-dissolved in water. The peptide was further purified by HPLC and a white powder was obtained by overnight lyophilization. The obtained peptides **DT-RGD** and **DT-DGR** were stored at -20 °C before use. HR-MS (ESI) m/z calcd. $C_{26}H_{43}N_{11}O_{11}S_2$.: 749.26; found: 750.2656 $[M+H]^+$.

S3. Multicomponent hydrogels preparation and gel inversion experiments

The stock solutions (10.0 mM) of **SQ** and **SQ-DT** were separately prepared by dissolving each monomer in DMSO with 2 min vortexing. To obtain the different multicomponent hydrogel systems, namely **SQ/xSQ-DT** (x: mole percent of **SQ-DT** of the total monomer concentration), the required volume of each monomer stock solution (10.0 mM) was pipetted into the glass vials (2.0 mL) with 30 s of gentle vortexing. DMSO was removed using a stream of N_2 overnight to obtain dried films with the mixed monomers and was further freeze-dried overnight. PBS (pH 7.4, 500-1000 µL) was then added and sonicated for 20-60 min (depending on the power of the sonication bath and the total monomer concentration) in an ice bath (~4 °C) to obtain optically clear solutions. These solutions were then incubated in an oven at 37 °C for 15 min to allow gelation to occur. The transparent hydrogels were equilibrated at room temperature overnight prior to performing further experiments.

The preparation of the multicomponent hydrogel **SQ/xSQ-DT/ySQ-RGD** (x and y: mole percent of **SQ-DT** and **SQ-RGD** of the total monomer concentration) followed the same procedure as above.

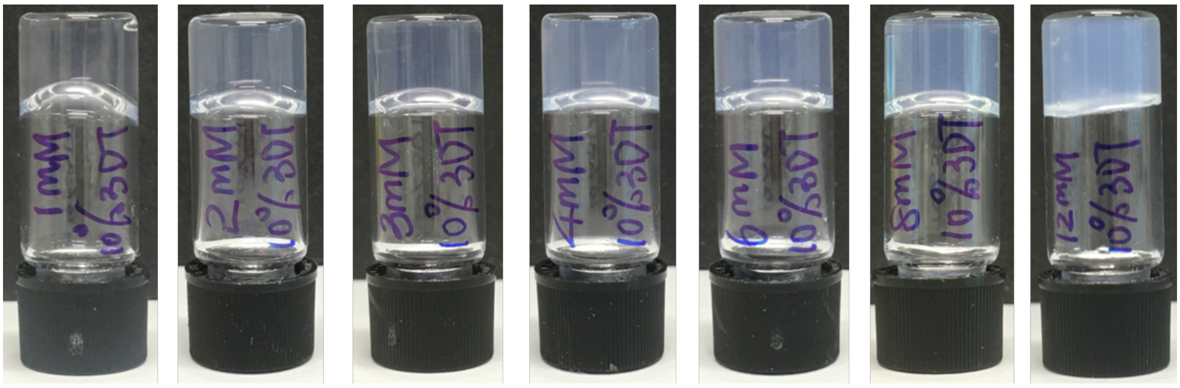

Figure S1. Gel inversion test of multicomponent squaramide-based supramolecular hydrogels (**SQ/10SQ-DT**, 10 mol% **SQ-DT**) with varied total monomer concentrations (1.0-12.0 mM) in PBS (pH 7.4) after overnight equilibration at room temperature.

S4. Oscillatory rheology

After overnight equilibration, the pre-made hydrogels were gently pipetted onto a 20 mm quartz plate and the gap distance between the parallel plates was set to 300 μm. Before data collection, the loaded samples were equilibrated for 5 min to allow the hydrogels to self-recover. After a time sweep experiment for 60 s, the hydrogels were stiffened by applying UV light through the Excelitas Omnicure S2000 system (λ = 320-500 nm, primary peak: 365 nm). All time sweep measurements were performed at a strain amplitude (γ) of 0.05% and at a frequency (f) of 1 Hz. Frequency sweeps were conducted in a frequency range of 0.01-10 Hz at a constant strain amplitude of 0.05%. Strain sweeps were performed in a range of strain amplitudes of 0.01-500% at a fixed frequency (1 Hz). To measure the self-recovery property of the gel before and after UV irradiation, a step-strain measurement was performed. First, a low strain amplitude of 0.05% (f = 1 Hz) was applied for 120 s. Once the storage modulus (*G'*) reached a plateau, a higher strain amplitude of 300% was applied for 120 s to break the hydrogels. Subsequently another time sweep at a low strain amplitude of 0.05% (f = 1 Hz) was performed to test the recovery of the sample. These strain amplitude was alternated in this manner for two cycles.

The nonlinear elastic regime was probed using a pre-stress method, where the hydrogel was subjected to a constant pre-stress upon which a small oscillatory stress was superimposed, $\delta\sigma(t) = \delta\sigma e^{iwt}$, up to a maximum of 10% of the constant pre-stress at a fixed frequency of 1 Hz. Stress relaxation measurements were performed at 10% strain.

Creep and recovery tests were performed after a time sweep of 1200 s with 0.01% strain amplitude (γ) at a frequency (f) of 1 Hz. In the creep test, the time-dependent strain deformation in response to a constant applied stress of 5 Pa was measured for 3600 s. Subsequently, in the recovery phase the stress was removed and the strain was measured for another 10,000 s. Water was added to the top of the cover surrounding the geometry to reduce drying of the hydrogels.

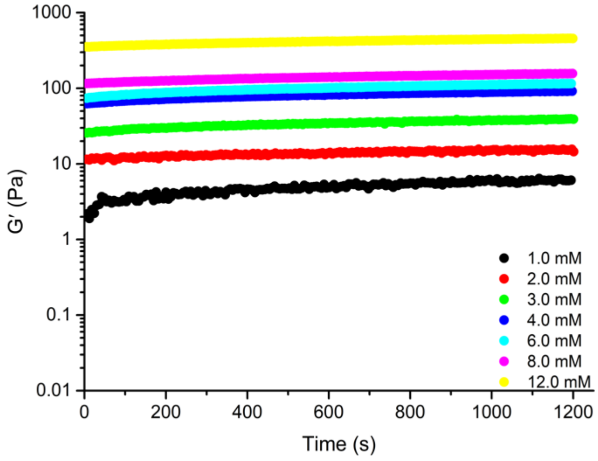

Figure S2. Averaged (N = 3) time sweep measurements of **SQ/10SQ-DT** with different total monomer concentrations without UV irradiation at fixed strain amplitude (γ = 0.05%) and frequency (f = 1 Hz) at room temperature.

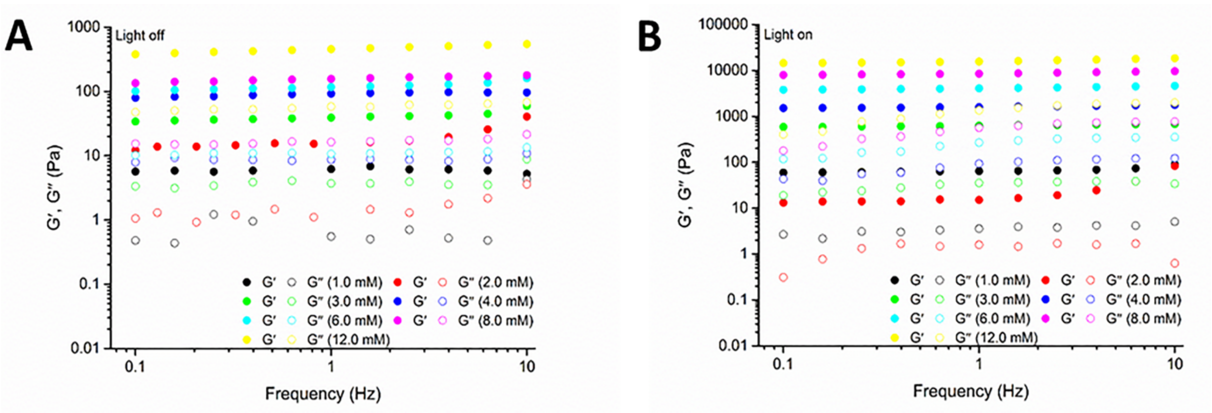

Figure S3. Averaged (N ≥ 3) frequency sweep (from 0.1 to 10 Hz) of **SQ/10SQ-DT** with different total monomer concentrations (A) without and (B) with 10 min UV irradiation (~10 mW/cm^2, 320-500 nm with a primary peak at 365 nm) at a fixed strain amplitude (γ = 0.05%) at room temperature.

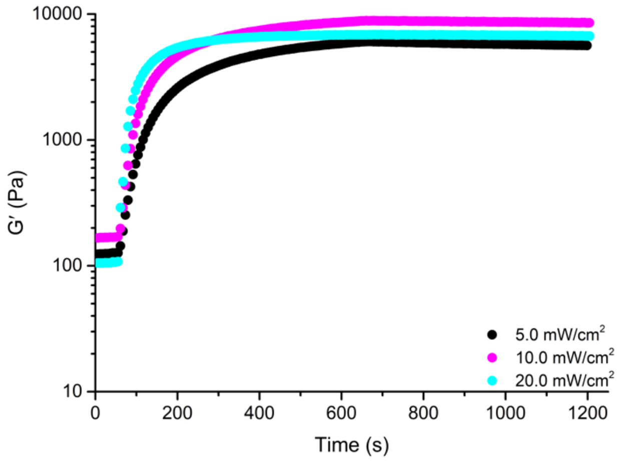

Figure S4. Averaged (N = 3) time sweep of **SQ/10SQ-DT** (8.0 mM) with 10 min UV irradiation (320-500 nm with maximum absorbance at 365 nm) using different light intensities (~5 mW/cm^2, ~10 mW/cm^2, and ~20 mW/cm^2) at a fixed strain amplitude (γ = 0.05%) and frequency (f = 1 Hz) at room temperature.

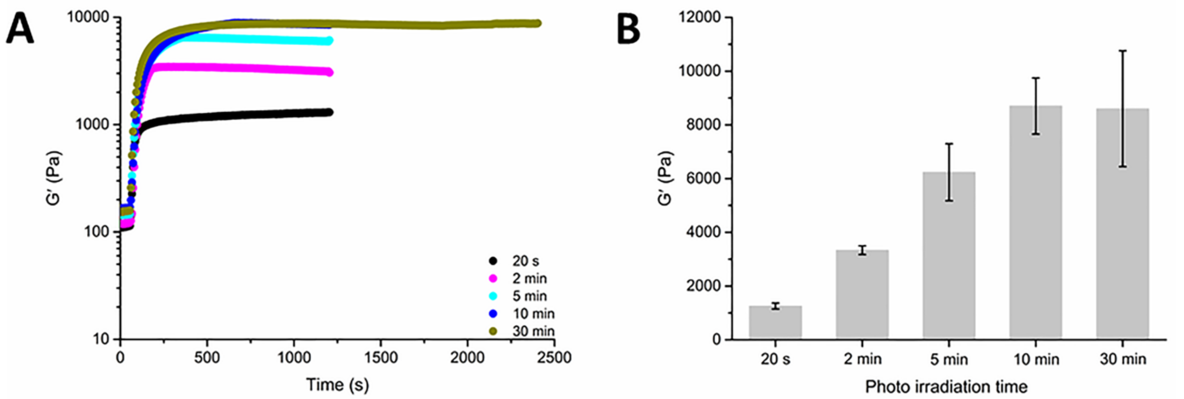

Figure S5. Averaged (N = 3) (A) time sweep and (B) storage modulus (*G'*) at plateau of **SQ/10SQ-DT** (8.0 mM) with different UV irradiation times (20 s, 2 min, 5 min, 10 min and 30 min) at room temperature (~10 mW/cm^2, 320-500 nm with maximum absorbance at 365 nm) using a fixed strain amplitude (γ = 0.05%) and frequency (f = 1 Hz). Error bars were calculated as the standard deviation of repeat measurements (N = 3).

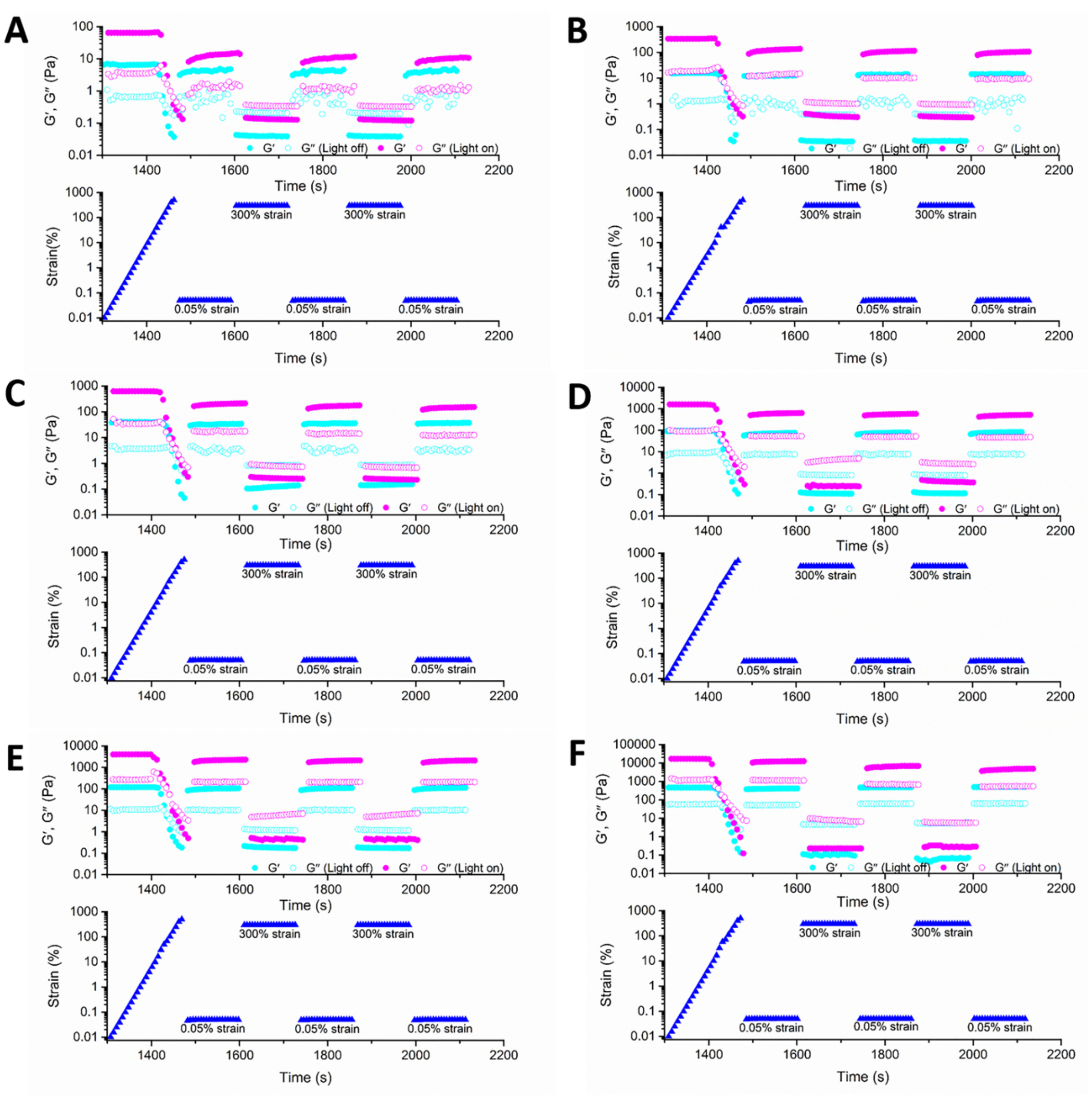

Figure S6. Averaged (N ≥ 3) strain sweep and step-strain experiments of the hydrogel **SQ/10SQ-DT** without and with 10 min light irradiation (~10 mW/cm^2, 320-500 nm with a primary peak at 365 nm), all at a fixed frequency (f = 1 Hz) at room temperature with different total monomer concentrations: (A) 1.0 mM, (B) 2.0 mM, (C) 3.0 mM, (D) 4.0 mM, (E) 6.0 mM and (F) 12.0 mM.

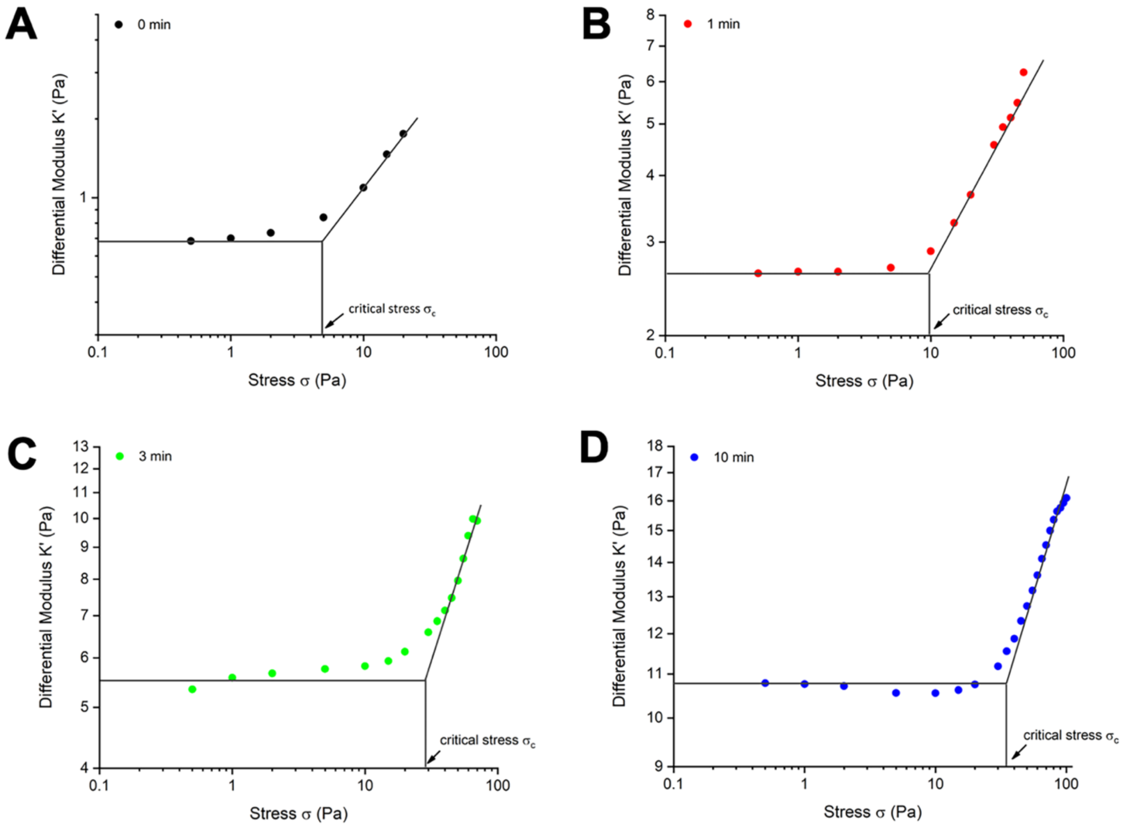

Figure S7. The differential modulus (*K'*) as a function of the applied pre-stress (σ) on **SQ/10SQ-DT** (4.0 mM) hydrogels with different UV irradiation times: (A) 0 min, (B) 1 min, (C) 3 min and (D) 10 min at room temperature (~10 mW/cm^2, 320-500 nm with maximum absorbance at 365 nm). The critical stress (σ_c) was graphically obtained from the intersection between linear and nonlinear regimes.

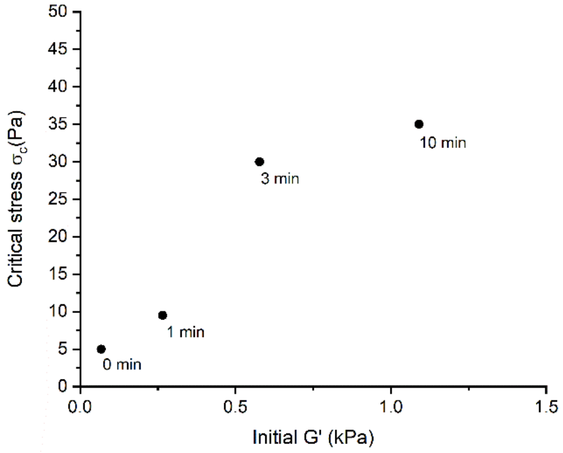

Figure S8. Critical stress, σ_c, as a function of initial storage modulus, *G'*, **SQ/10SQ-DT** (4.0 mM) hydrogels prior to various UV irradiation times (0 min, 1 min, 3 min and 10 min) at room temperature (~10 mW/cm^2, 320-500 nm with a maximum absorbance at 365 nm).

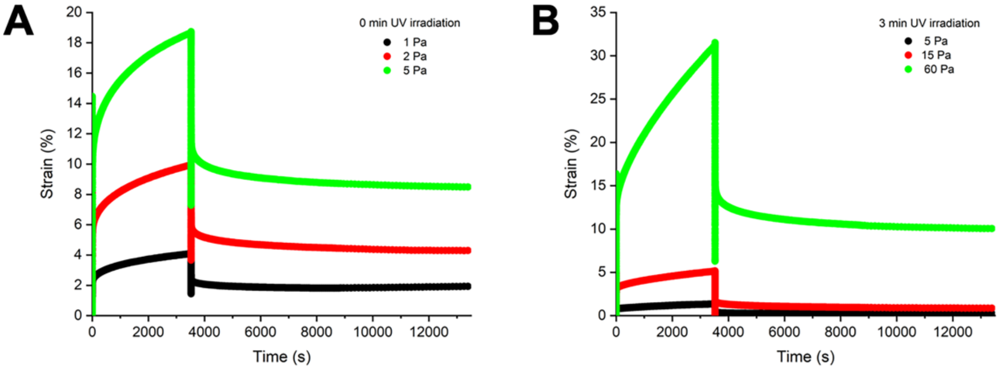

Figure S9. Creep and recovery test of **SQ/10SQ-DT** (4.0 mM) hydrogels with different UV irradiation times (A) 0 min and (B) 3 min. Different constant stresses were used during the creep test. Conditions for UV irradiation: ~10 mW/cm^2, 320-500 nm filter with maximum absorbance at 365 nm.

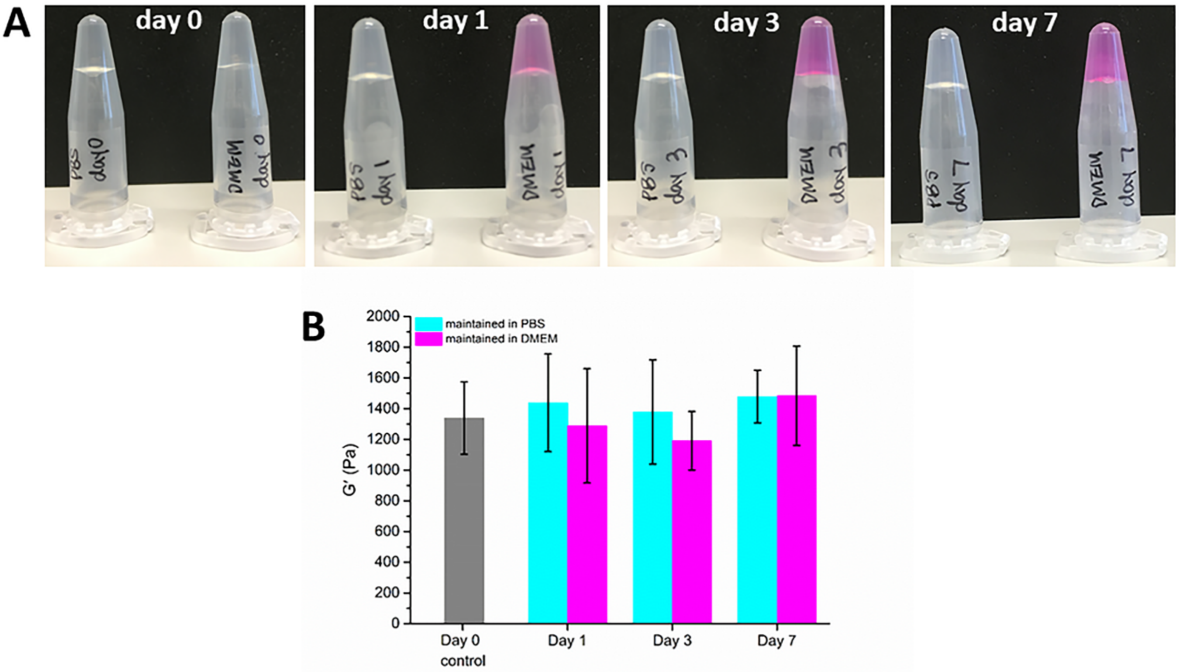

Figure S10. (A) Representative gel inversion tests and (B) averaged (N = 2) storage moduli (*G'*) at plateau of the stiffened **SQ/10SQ-DT** (4.0 mM) hydrogels using UV irradiation (10 min, ~10 mW/cm^2, 320-500 nm with maximum absorbance at 365 nm) before and after maintenance in PBS (pH 7.4) or cell culture medium DMEM containing 20% serum at 37°C at different time points (e.g. day 1, 3 and 7). The PBS and DMEM medium were changed every day during the maintenance. Error bars were calculated as the standard deviation of repeat measurements (N = 2).

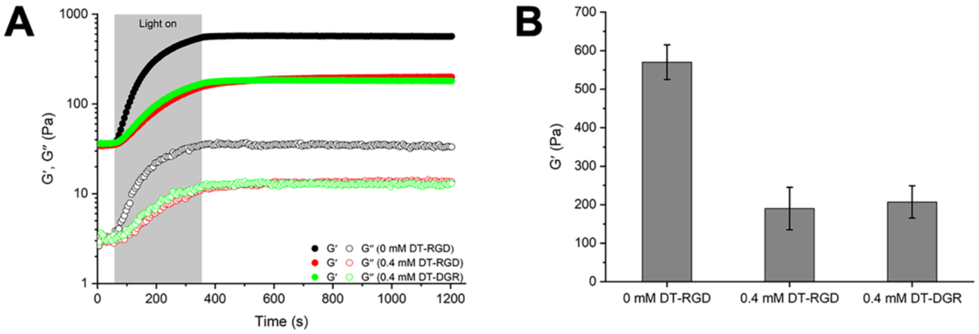

Figure S11. Averaged (N = 3) (A) time sweep and (B) storage moduli (*G'*) at plateau of the hydrogel **SQ/10SQ-DT** (3.0 mM) without adding peptide or with peptide **DT-RGD** (0.4 mM) or **DT-DGR** (0.4 mM) with 5 min UV irradiation at room temperature (~10 mW/cm^2, 320-500 nm with maximum absorbance at 365 nm) measured using a fixed strain (γ = 0.05%) and frequency (f = 1 Hz). Error bars were calculated as the standard deviation of repeat measurements (N = 3).

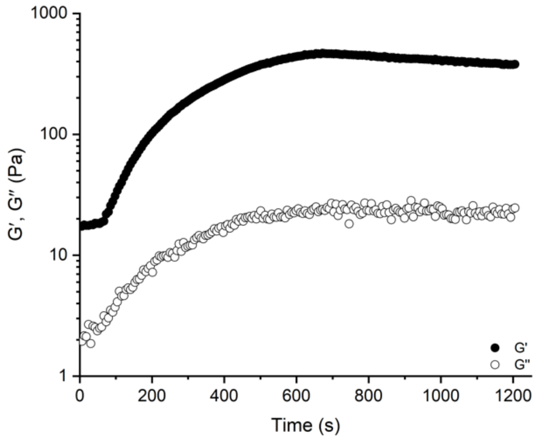

Figure S12. Average (N = 2) time sweep experiment of **SQ/10SQ-DT/10SQ-RGD** (4.0 mM) hydrogel with 5 min UV irradiation (~10 mW/cm^2, 320-500 nm with a maximum absorbance at 365 nm) at a fixed strain (γ = 0.05%) and frequency (f = 1 Hz) at room temperature.

S5. Solid-state NMR spectroscopy

Two samples were prepared for solid-state NMR spectroscopy: (1) multicomponent hydrogel **SQ/10SQ-DT** (8.0 mM) in MilliQ water without and with 10 min UV irradiation using a benchtop LED source (~10 mW/cm^2, 375 nm); (2) **PEGdiDT**[2] (6.0 mM) in MilliQ water without and with 30 min UV irradiation using a benchtop LED source (~10 mW/cm^2, 375 nm) (as a control sample). The MilliQ water was removed through freeze drying to obtain solids (~ 30 mg for each condition) prior to measurement.

NMR measurements were recorded on a Bruker Avance-I 750 MHz wide bore solid-state NMR spectrometer with 17.6 T magnetic field. In this field, ^{13}C and ^{1}H resonate at 188.66 and 750.23 MHz respectively. A standard 3.2 mm triple resonance E-free MAS probe was used. All the samples were packed in 3.2 mm thick-walled zirconium rotors with Vespel caps and were spun at magic angle (54.74°) at a spinning speed of 15 kHz. The temperature was kept constant at 298 K.

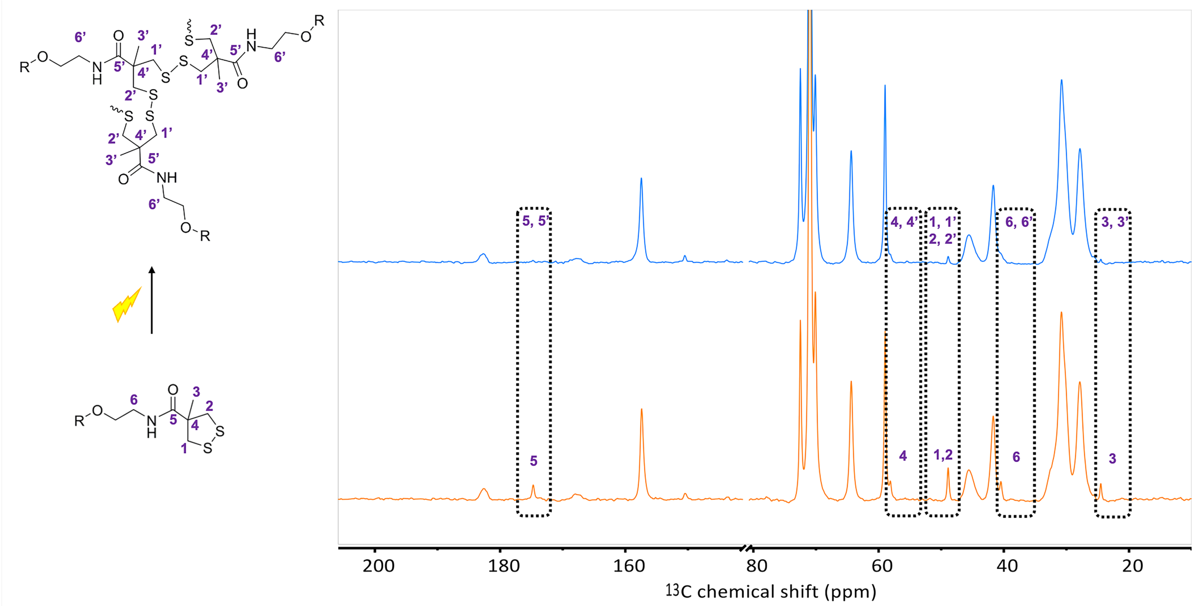

Figure S13. Proposed **SQ-DT** reaction product (*left*) and 1D ^{13}C CPMAS spectra of multicomponent materials **SQ/10SQ-DT** (8.0 mM) with 0 min (*orange line*) and 10 min (*blue line*) UV irradiation using a benchtop LED source (~10 mW/cm^2, 375 nm).

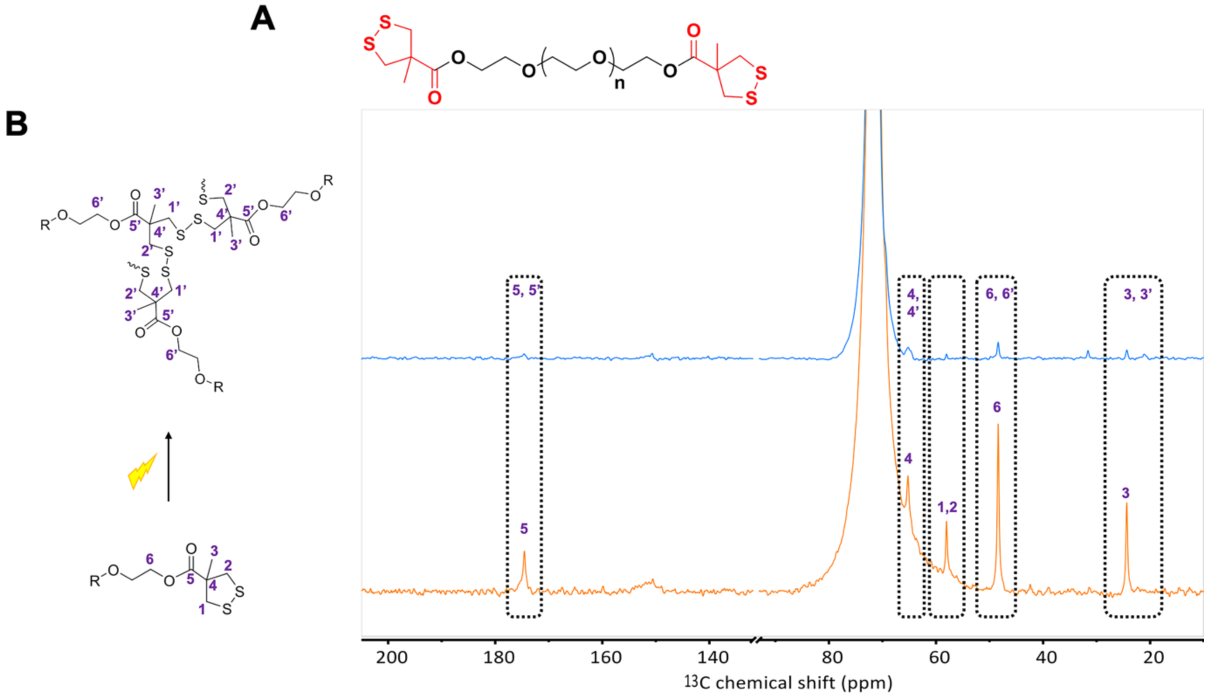

Figure S14. (A) Chemical structure of control monomer **PEGdiDT** (~6 kDa). (B) Proposed reaction product and 1D ^{13}C CPMAS spectra of **PEGdiDT** (6.0 mM) with 0 min (*orange line*) and 30 min (*blue line*) UV irradiation using a benchtop LED source (~10 mW/cm^2, 375 nm).

S6. Ellman's assay study

S6.1. Calibration curve for Ellman's assay

DL-Dithiothreitol (DTT) was used to prepare a calibration curve for the Ellman's test on the SQ-DT hydrogels. Stock solutions of DTT (5.0 mM) and the Ellman reagent (5,5′-dithiobis-(2-nitrobenzoic acid)) (DTNB) (1.8 mM) were prepared separately in phosphate buffer (10.0 mM, pH~8). The DTT stock solution (5.0 mM) was diluted using phosphate buffer to various concentrations (e.g. from 0.1-1.9 mM in 0.2 mM increments). An aliquot of the DTNB stock solution (25 µL) was then allowed to react with each of the pre-made DTT solutions (25 µL) for 10 min. Each solution was further diluted with phosphate buffer (950 µL) resulting in solutions containing the same amount of DTNB (45 µM) and a varied DTT (e.g. 0 µM, 2.5 µM, 7.5 µM, 12.5 µM, 17.5 µM, 22.5 µM, 27.5 µM, 32.5 µM, 37.5 µM, 42.5 µM and 47.5 µM) concentration. A UV-Vis spectrum of each solution was then collected at room temperature. A calibration curve was prepared using the absorbance of DTT at 412 nm. The obtained equation from the linear regression of the calibration curve was $Abs_{412\ nm} = 0.02686x\ (\mu M) + 0.00844$.

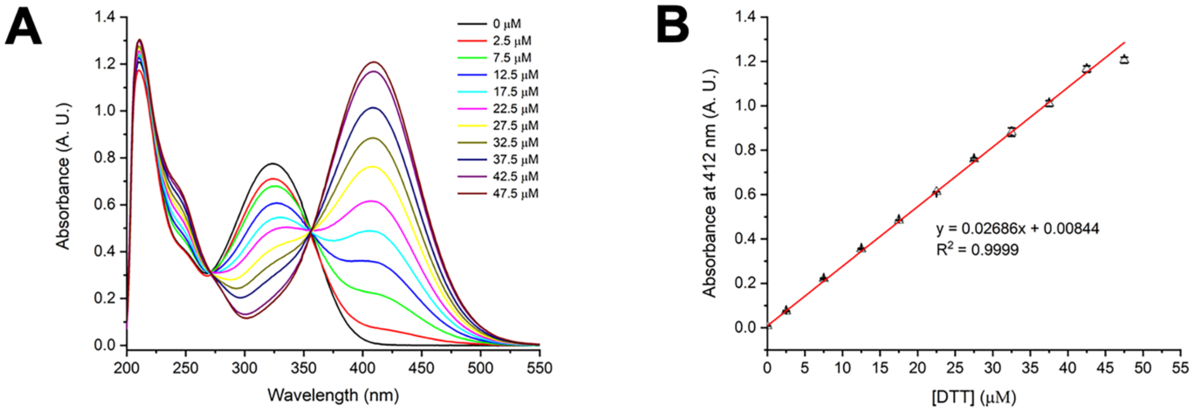

Figure S15. (A) UV-Vis spectra of solutions with increasing concentrations of DTT (0-47.5 µM) and DTNB (45 µM) in phosphate buffer (pH 8.0) at room temperature. (B) Calibration curve for Ellman's assay using DTT based on the absorbance intensity at 412 nm.

S6.2. Ellman's assay on supramolecular polymer solutions and hydrogels before and after UV irradiation

The pre-gel solutions of **SQ/10SQ-DT** (4.0 mM) in PBS (pH 7.4) after sonication in an ice bath were either directly (4.0 mM, 500 µL) pipetted or further diluted with PBS (pH 7.4) (0.4 mM, 500 µL) before pipetting into a new glass vial (2 mL). Both the gel and solution samples were independently equilibrated at 37°C for 15 min and kept overnight at room temperature. From these solutions, an aliquot (100 µL of each solution) was transferred to a glass vial to examine the effect of different UV irradiation times on the free thiol concentration.

Solutions of **SQ/10SQ-DT** (0.4 mM) were irradiated with UV light for three different durations (0 min, 5 min, and 10 min) using a benchtop LED source (~10 mW/cm^2, 375 nm). An aliquot (25 µL) was then pipetted out from each solution and allowed to react with an equal amount of the DTNB stock solution (25 µL) at room temperature for 10 min. The samples were further diluted with phosphate buffer (950 µL) (**SQ/10SQ-DT** (0.1 mM) and DTNB (45 µM)) prior to UV-Vis measurement.

Hydrogels of **SQ/10SQ-DT** (4.0 mM) were irradiated with UV light for different durations (0 min, 5 min, and 10 min) using a benchtop LED source (~10 mW/cm^2, 375 nm). An aliquot of the DTNB stock solution (600 µL) was then applied on top of the hydrogels at room temperature for 10 min. After the reaction, the supernatant (25 µL) was removed and further diluted with phosphate buffer (975 µL) before taking a UV-Vis spectrum at room temperature.

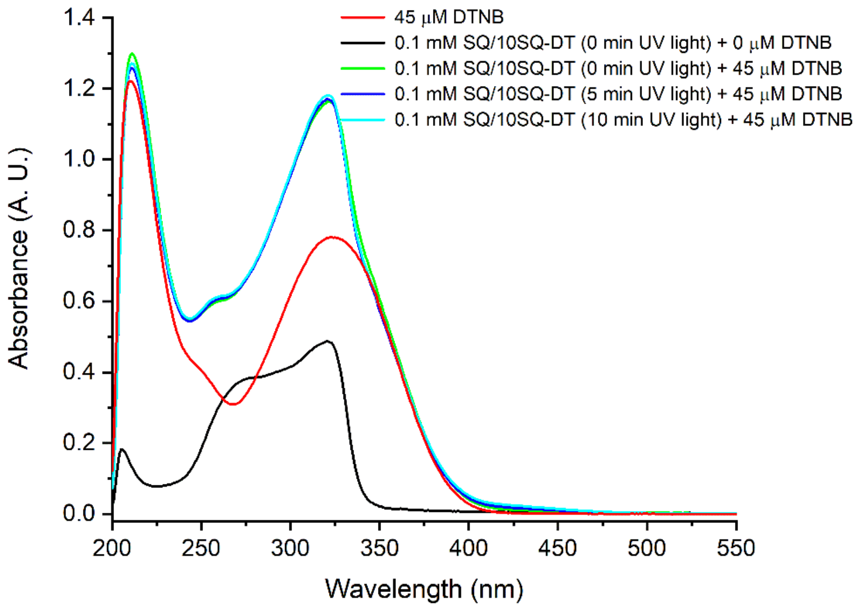

Figure S16. Ellman's test on **SQ/10SQ-DT** solutions at room temperature. **SQ/10SQ-DT** (0.1 mM) and DTNB (45 μM) with different UV irradiation times (0 min, 5 min, and 10 min) using a benchtop LED source (~10 mW/cm^2, 375 nm). **SQ/10SQ-DT** (0.1 mM) and DTNB (45 μM) were selected as independent control samples.

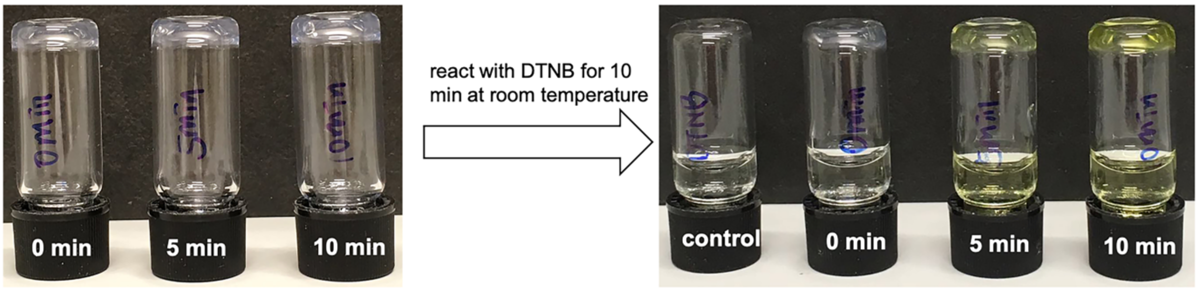

Figure S17. Photographs of **SQ/10SQ-DT** (4.0 mM) hydrogels prepared with different UV irradiation times (0 min, 5 min, and 10 min) before and after treatment with DTNB (45 μM) for 10 min at room temperature. A benchtop LED source (~10 mW/cm^2, 375 nm) was used to apply UV light to the hydrogels.

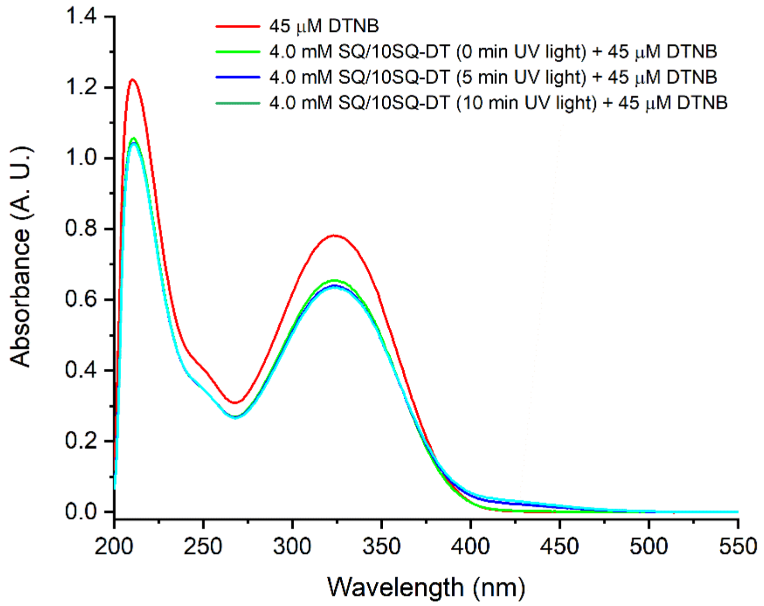

Figure S18. Ellman's test on **SQ/10SQ-DT** (4.0 mM) gels at room temperature. **SQ/10SQ-DT** (4.0 mM) and DNTB (45 μM) with different UV irradiation times (0 min, 5 min, and 10 min) using a benchtop LED source (~10 mW/cm^2, 375 nm). DTNB solution (45 μM) was selected as a control sample.

Table S1. Average (N = 2) absorbance at 412 nm for the various Ellman's test samples.

Samples	Control 1	Control 2	Solution 1	Solution 2	Solution 3	Gel 1	Gel 2	Gel 3
UV irradiation (min)	0	0	0	5	10	0	5	10
Averaged absorbance at 412 nm	0.0084 ± 0.00035	0.0047 ± 0.0026	0.0016 ± 0.0058	0.023 ± 0.0036	0.026 ± 0.0053	0.0084 ± 0.00086	0.021 ± 0.0069	0.032 ± 0.0087

Control 1: 0.1 mM **SQ/10SQ-DT** with 0 μM DTNB; Control 2: 45 μM DTNB; Solution 1-3: 0.1 mM **SQ/10SQ-DT** with 45 μM DTNB; Gel 1-3: 4.0 mM **SQ/10SQ-DT** with 45 μM DTNB. UV irradiation was applied using a benchtop LED source (~10 mW/cm^2, 375 nm).

S7. Diffusion study using the fluorescence recovery after photobleaching (FRAP) technique

The multicomponent supramolecular hydrogel **SQ/10SQ-DT** (4.44 mM) was mixed with solutions of fluoresceinamine (Mw = 0.347 kDa, 1.0 mM), FITC-dextran (Mw~10 kDa, 0.5 mM) or FITC-dextran (Mw~70 kDa, 0.15 mM) in PBS in a volume ratio of 9:1 (gel: fluorophore/fluorophore-labelled dextran). Thus, samples of **SQ/10SQ-DT** (4.0 mM) contained fluoresceinamine (100 μM), FITC-dextran (10 kDa) (50 μM), or FITC-dextran (70 kDa) (15 μM). The hydrogel-fluorophore mixtures (12 μL) were gently pipetted into a μ-Slide 8-well plate and irradiated with UV light (0 min or 5 min) using a benchtop LED source (~10 mW/cm^2, 375 nm) prior to placement on the confocal microscope.

FRAP experiments were performed with a 40x objective (Plan Fluor, Nikon) on a spinning disk confocal microscope (Nikon Eclipse Ti, Yokogawa confocal spinning disk unit operated at 10,000 rpm). Samples were recorded with an Andor iXon Ultra 897 high speed EM-

CCD camera to obtain 512 x 512 pixel images with a resolution of 0.33 μm/pix. Prior to bleaching, the hydrogel-fluorophore mixtures were imaged for 2 s with a 40 ms framerate. Then, a circular bleaching ROI ($r = 12$ μm), 40 μm into the hydrogel, was excited for 5 s (2 ms/pix dwell time) using a 488 nm Argon laser through an Andor FRAPPA unit. After bleaching, recovery images were recorded with a frame rate of 40 ms. All imaging was done at low laser excitation (1-10%) to prevent further bleaching and reference ROIs were used to normalize the recorded intensities at the bleaching spot.

Figure S19A-B shows example recovery signals recorded for the hydrogel mixtures with fluoresceinamine (*light-grey*), 10 kDa FITC-dextran (*grey*) and 70 kDa FITC-dextran (*dark-grey*). The intensities were normalized as: $f_r(t) = \frac{f_{ROI}(t)/f_{ref}(t) - f_{ROI}(0)/f_{ref}(0)}{f_{ROI}(\infty)/f_{ref}(\infty) - f_{ROI}(0)/f_{ref}(0)}$, here $f_r(t)$ is the recovery signal, $f_{ROI}(t)$ the intensity of the bleached region and $f_{ref}(t)$ the intensity of the ROI, following Liu *et al.*[4] The recovery curves are well fit by an equation with a single exponential: $f_r(t) = A(1 - e^{-\frac{t}{t_0}})$, where t_0 can be used to determine the half-time of recovery by $t_{1/2} = t_0 \ln 2$. The averaged ($n > 5$) half times show that cell-culture associated biomolecules, like nutrients and growth factors, can adequately diffuse through the hydrogel (**Figure S19C**).

The diffusion constant was estimated by fitting the normalized recovery curves to: $f(t) = \sum_{n=0}^{\infty} \frac{-K^n}{n!} \frac{1}{1+n\left[1+\left(\frac{2t}{\tau_D}\right)\right]}$, where τ_D is the 2-D characteristic diffusion time and K is the bleaching constant that depends on the experimental system.[5] For a Gaussian laser beam the diffusion constant is related to τ_D by $D = \omega^2/4\tau_D$, where ω is half the width of Gaussian laser profile determined at e^{-2} of the profile height. The radius was determined by fitting a Gaussian to the bleach spot (identical settings and depth to supramolecular hydrogels) of dried-out agar (2%)-fluoresceinamine (100 μM) samples and found to be $\omega = 17.1 \pm 1.6$. The diffusion constants are shown in **Table S2**. All calculations were performed in Matlab 2019a, using the curve-fitting and image-processing toolboxes.

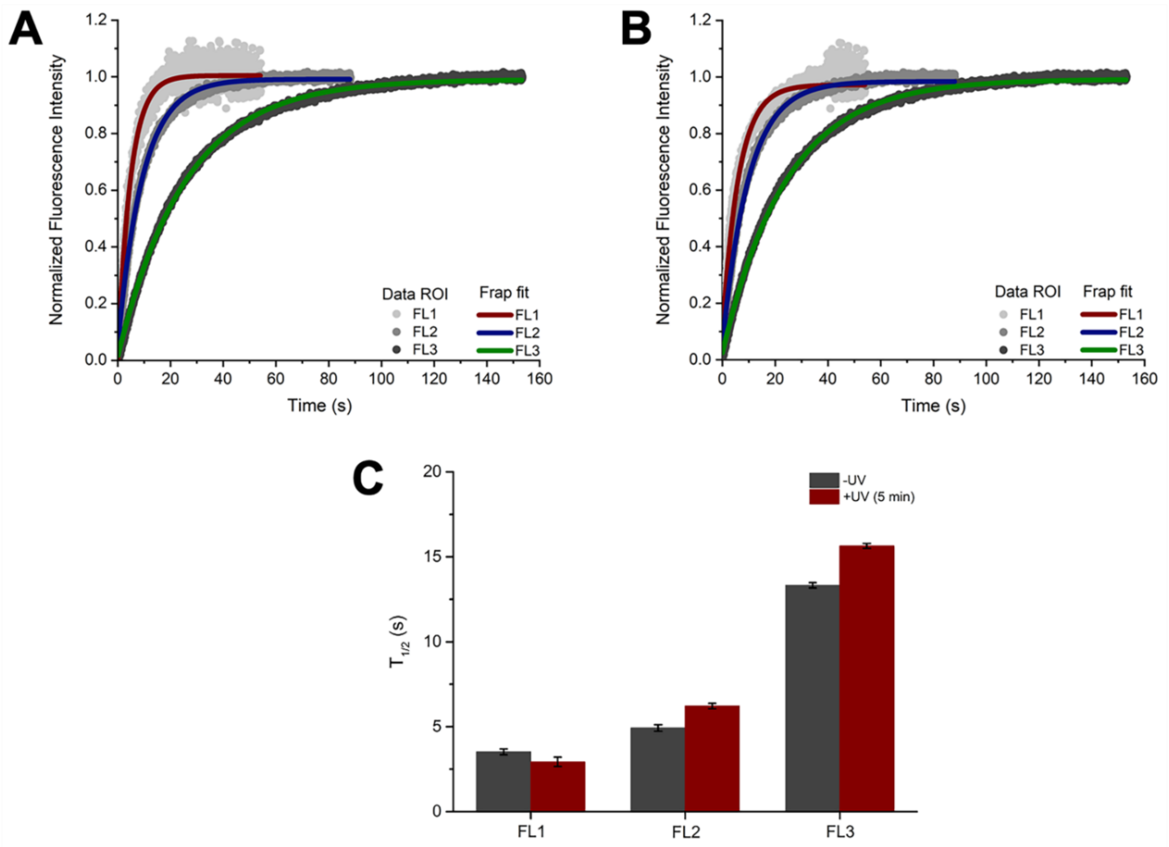

Figure S19. Characterization of permeability of the multicomponent supramolecular hydrogels **SQ/10SQ-DT** (4.0 mM) without (A) and with 5 min UV irradiation (B) by studying the diffusion of differently sized fluorescent molecules. Dots: FRAP curves; Lines: related curve fits. The normalized intensities FL1-3 (and fits) are representative curves selected from a series of repeated experiments (C) Calculated half recovery times of hydrogels without and with 5 min UV irradiation. UV irradiation condition: ~10 mW/cm^2, 375 nm. Fluorophores: FL1 (Fluoresceinamine (Mw = 0.347 kDa)); FL2 (FITC-dextran (Mw~10 kDa)); FL3 (FITC-dextran (Mw~70 kDa)). Error bars are the standard deviation of the fit parameters found for all measurements per condition.

Table S2. Effect of hydrogel stiffness and molecular size on the diffusion coefficient (D) (μm^2/s).

Hydrogel **SQ/10SQ-DT** (mM)	UV irradiation time (min)	Molecule size (kDa) 0.347	10	70
4.0	0	20.76 ± 3.69	9.78 ± 1.74	2.99 ± 0.53
4.0	5	18.69 ± 3.32	9.64.76 ± 1.71	3.17 ± 0.56

S8. Cryogenic Transmission Electron Microscopy (cryo-TEM)

Multicomponent hydrogels **SQ/10SQ-DT** (2.0 mM) were first prepared and equilibrated overnight before use. The hydrogels (100 μL) were pipetted into a new glass vial (2 mL) and UV irradiation was applied for 10 min using a bench top LED source (~10 mW/cm^2, 375 nm) at room temperature. After that, hydrogel samples (3 μL) were separately pipetted without further dilution onto freshly glow-discharged copper grids for each UV irradiation time (0 min and 10 min), and the excess liquid was blotted away for 2 s using Whatman No. 4 filter paper (98% humidity) and plunge-frozen in a mixture liquid ethane and propane at -196 °C using a Leica EM GP (Leica Microsystems). The cryo-TEM samples were stored in liquid nitrogen before imaging on a Tecnai F12 microscope (FEI).

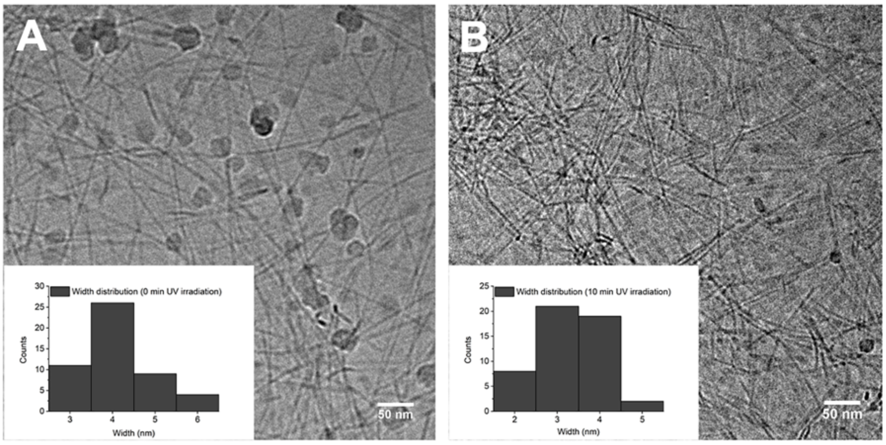

Figure S20. Cryo-TEM images of multicomponent materials **SQ/10SQ-DT** (2.0 mM) with (A) 0 min and (B) 10 min UV irradiation using a benchtop LED source (~10 mW/cm^2, 375 nm). Scale bar: *50 nm*. The insert figures indicate the histograms of the width distribution of the counted fibers (N = 50) from cryo-TEM images. Ice crystal contamination is observed as black spots in the images.

S9. Small angle X-ray scattering (SAXS)

The **SQ/10SQ-DT** supramolecular hydrogels (0.8 mM and 2.0 mM) were prepared and left to stand overnight before use. The hydrogel samples prior to UV irradiation were separately pipetted into quartz capillaries (2.0 mm) and SAXS measurements were performed. After that, the samples in the quartz capillaries were directly irradiated with UV light (~10 mW/cm^2, 365 nm) for 5 min and measured.

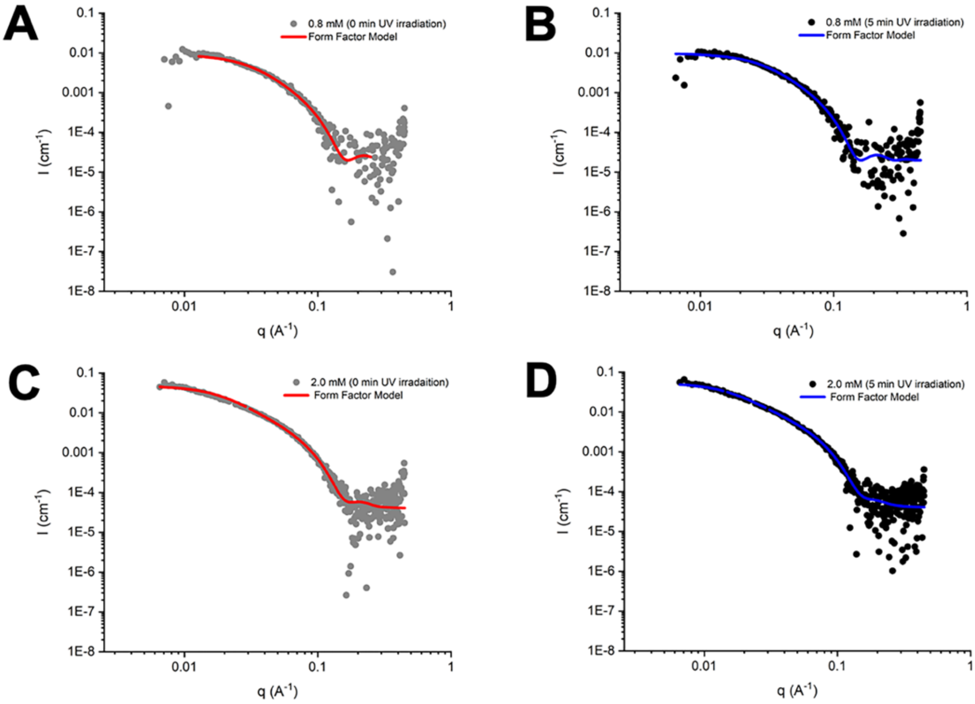

Figure S21. Small-angle X-ray scatting profiles of supramolecular hydrogels **SQ/10SQ-DT**: (A) hydrogel concentration of 0.8 mM, without UV irradiation; (B) hydrogel concentration of 0.8 mM, with 5 min UV irradiation; (C) hydrogel concentration of 2.0 mM, without UV irradiation; and (D) hydrogel concentration of 2.0 mM, with 5 min UV irradiation. Dots: experimental data; lines: fit a form factor model of flexible cylinder.

S10. Cell culture and lentiviral transduction

S10.1. Cell culture

NIH 3T3 and C2C12 cell lines were cultured in DMEM medium-high glucose (D6546) with 10% fetal bovine serum (FBS), 1% Glutamax, 0.2% penicillin and streptomycin. The Hs578T[6] cell line was cultured in RPMI 1640 medium with 10% FBS, 0.2% penicillin and streptomycin. All cells were cultured in an incubator at 37 °C with a 5% CO_2 atmosphere and kept below 70% confluency.

S10.2. Generation of stable mCherry-LifeAct reporter cell lines

For visualization of the actin cytoskeleton, C2C12 and Hs578T WT cells were transduced using a lentiviral mCherry-LifeAct cDNA expression vector (provided by Dr. Olivier Pertz, University of Basel, Basel, Switzerland), and were further cultured in selection medium containing 2 μg/mL puromycin in an incubator at 37 °C with a 5% CO_2 atmosphere.

S11. 3D cell encapsulation and cell viability assay

Cells were first dissociated from T-25 culture flasks (< 12 passages) by treatment with 0.25% trypsin for NIH 3T3 and Hs578T cells, and 0.25% trypsin-EDTA solution for C2C12 cells. The resulting cell suspensions (3.0×10^6 - 8.0×10^6 cells/mL) were centrifuged, and then re-suspended in fresh medium and mixed (15 μL) with the pre-prepared hydrogel **SQ/10SQ-DT** (4.44 mM, 135 μL) in PBS by gently pipetting up and down (~10 times) to obtain a homogeneous cell-laden hydrogel (4.0 mM **SQ/10SQ-DT**, 3.0×10^5 - 8.0×10^5 cells/mL). These hydrogels (12 μL) were then pipetted into a μ-slide 15-well angiogenesis plate and equilibrated for 5 min in an incubator at 37 °C. The various samples were then left as-is, or further stiffened using UV light from a benchtop LED source (5 min, ~10 mW/cm^2, 375 nm). The cell culture media (48 μL) was carefully layered on top of the hydrogels and placed in an incubator at 37 °C.

Cell viability was measured after 24 h using LIVE/DEAD (calcein AM/propidium iodide (PI)) staining. Prior to staining, the medium was carefully removed from the top of the hydrogel and washed with PBS (2 x 48 μL). The staining solution (48 μL) containing calcein AM (2.0 μM) and PI (1.5 μM) was pipetted on top of the hydrogel and incubated at 37 °C for 30 min. Then, the remaining staining solution was removed, and further washed with PBS (2 x 48 μL). Additional PBS (48 μL) was added on top of the hydrogel to avoid the hydrogel drying during imaging.

The stained cell-laden hydrogels were imaged on a Zeiss LSM 710 confocal laser scanning microscope equipped with a Zeiss 5× objective. Fluorescent Z-stack images (37-44 images/per sample) through the gel were acquired at a resolution of 512 x 512 pixels (0.69 μm/pixel) using an excitation wavelength of 488 nm and an emission filter of 519-582 nm for calcein AM, and an excitation wavelength of 532 nm and an emission filter of 615-695 nm for PI. The percentage of cell viability was determined by counting the viable (*green*) cells with calcein AM staining and dead (*red*) cells with PI-staining using Image J.

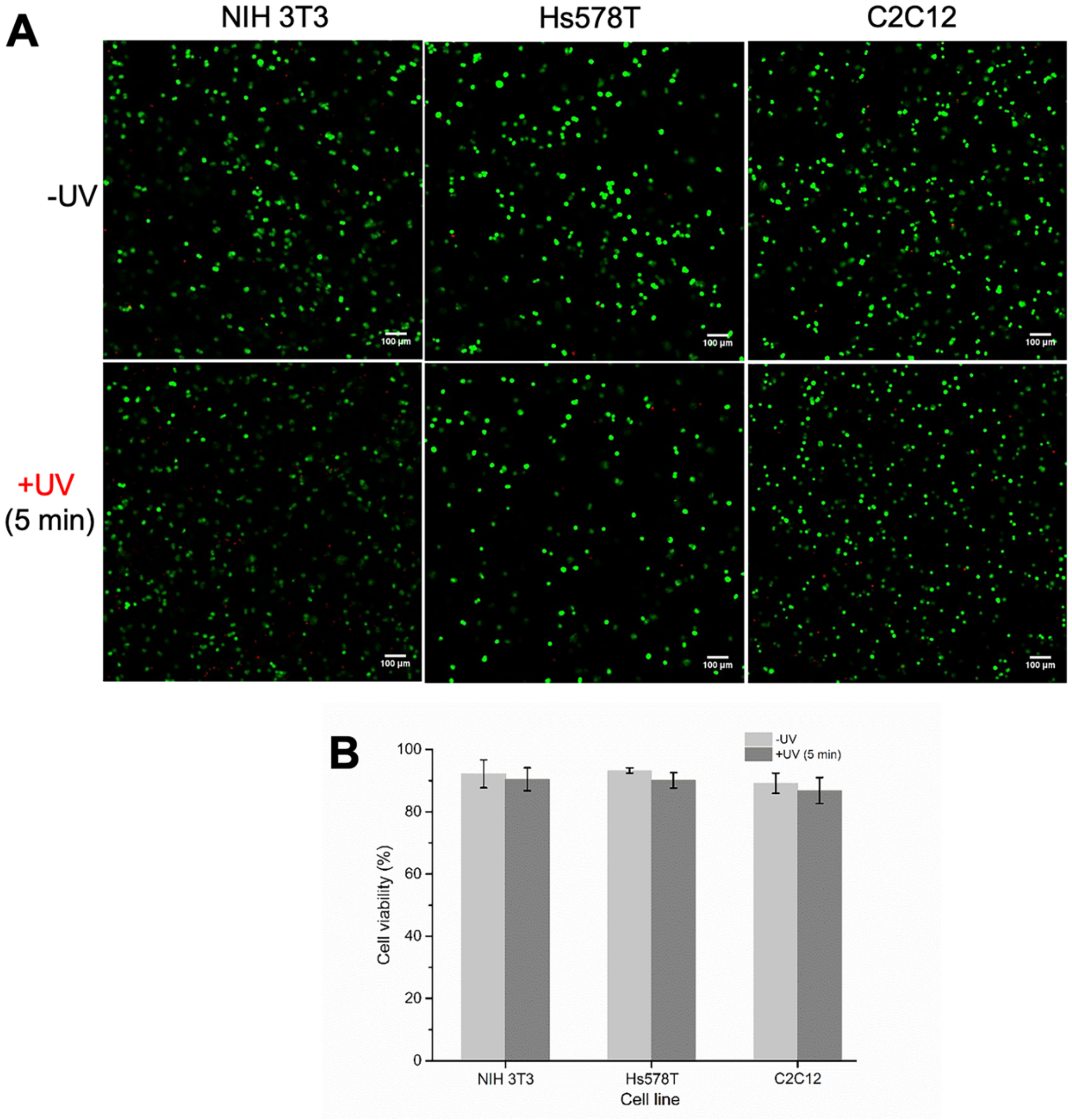

Figure S22. (A) Confocal microscopy images of three cell lines (NIH 3T3, Hs578T, and C2C12) stained by Calcein AM and PI and (B) quantification of averaged (N = 3) cell viability of the tested cell lines after 24 h encapsulation within hydrogel **SQ/10SQ-DT** (4.0 mM) without and with 5 min UV irradiation using a bench top LED (~10 mW/cm^2, 375 nm). Scale bar: *100 µm*. Viable cells are labeled in green and dead cells in red. Error bars are based on the standard deviation of repeat measurements (N = 3).

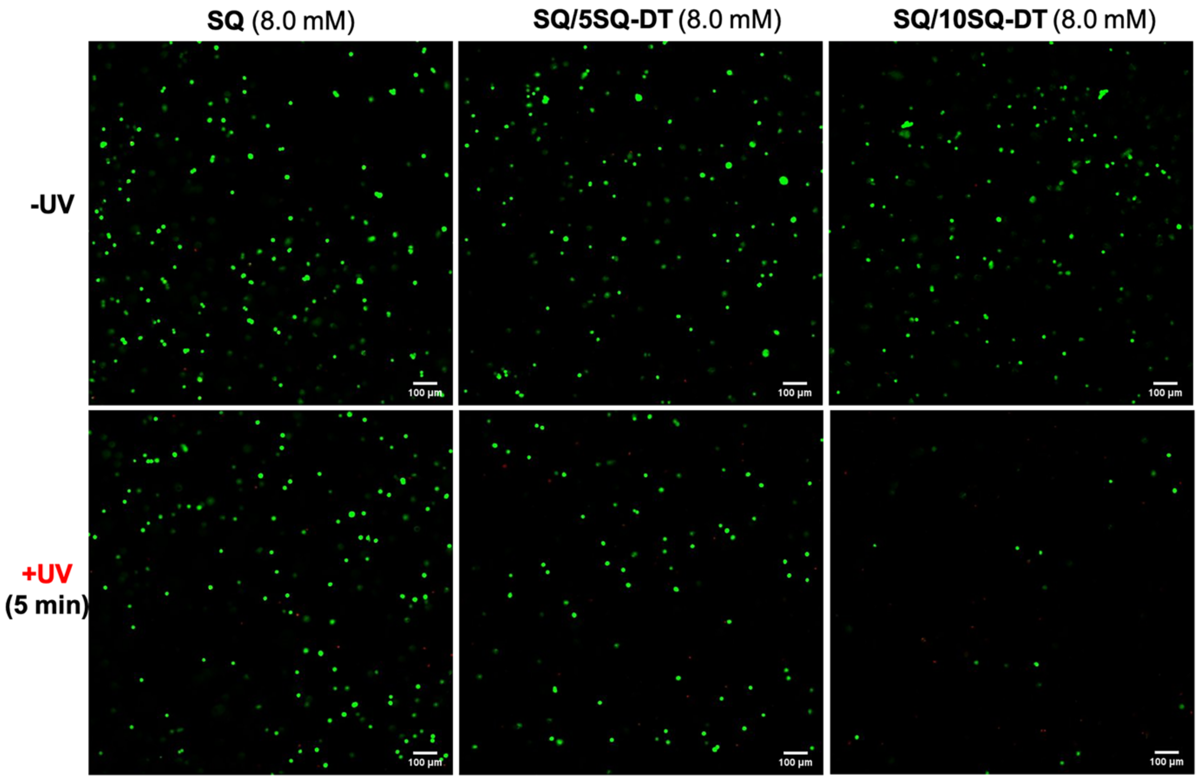

Figure S23. Representative confocal microscopy images of C2C12 cell line stained by Calcein AM and PI after 3 days culture in different supramolecular hydrogel systems (e.g. **SQ**, **SQ/5DT**, and **SQ/10SQ-DT**) containing the same total monomer concentration (8.0 mM) but with varied **SQ-DT** concentrations and UV irradiation times (0 min and 5 min). UV irradiation: ~10 mW/cm^2, 375 nm. Scale bar: *100 µm*. Viable cells are labeled in green and dead cells in red.

S12. mCherry-LifeAct Hs578T cell seeding on top of hydrogel (2D cell assay)

Pre-made **SQ/SQ-DT/SQ-RGD** (4.0 mM) hydrogels were pipetted into a µ-slide 4 well plate with custom made PDMS well insets (produced as described in section **15.1**) and equilibrated for 5 min in an incubator at 37 °C. The hydrogels were UV irradiated for different durations (0 and 5 min) using a benchtop LED source (~10 mW/cm^2, 375 nm). The mCherry-LifeAct Hs578T cell suspension (0.9 x 10^5 cells/mL) in cell culture medium was then gently pipetted on top the hydrogels. The cell and hydrogel samples were carefully placed in an incubator at 37 °C.

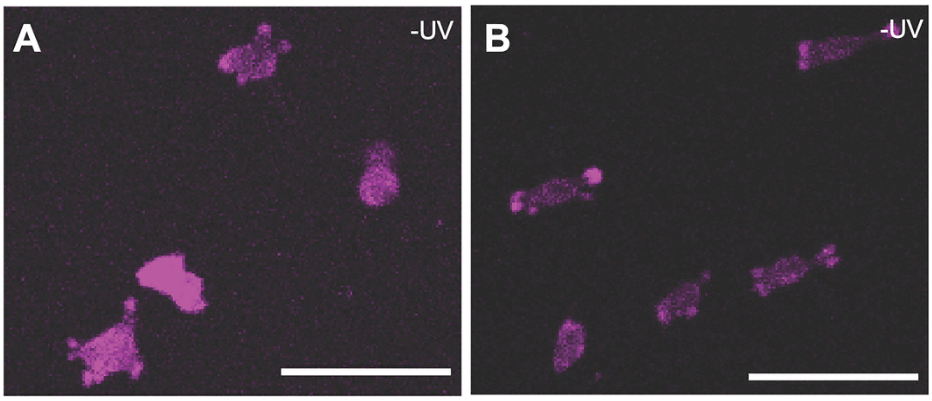

Figure S24. Representative images of mCherry-LifeAct Hs578T cells cultured for 6 h on top of a **SQ/10SQ-DT/10SQ-RGD** (4.0 mM) hydrogel with different UV irradiation times: (A) 0 min and (B) 5 min. Scale bar: 100 µm. UV irradiation was applied through a benchtop LED (~10 mW/cm^2, 375 nm).

S13. Quantification of cell spreading in 2D and 3D imaging data

An in-house MATLAB (MathWorks, 2019a) code that allowed automated adaption of edge-detection settings for every individual cell recognized was used to quantify cell spreading. This individual optimization of edge-detection parameters was necessary, especially for 3D data sets, as fluorescence intensities and signal-to-noise ratios varied notably per cell. Such variations were primarily due to differences in imaging height of each cell in the gel, but are also attributable to the inherent heterogeneity in LifeAct expression.

Before any edge detection, all images in every z-stack were preprocessed. First, illumination inhomogeneities in the microscope field of view were flattened using FIJI plugin BaSiC.[7] The images were then imported into Matlab 2019a. The appropriate contrast was set, the background was subtracted and images were despeckled by median filtering using an in-house code. In case of 2D spreading data, only the top of the hydrogel was imaged and the thin z-stacks were immediately maximally projected for edge detection analysis. In case of 3D spreading data, there were larger variations in fluorescence intensity and a custom written image analysis Matlab application was used to analyze data, which is described below. For both 2D and 3D data, cell-edges were determined using the Sobel method (thresholded 0.01-0.1), and resulting binary edge images were dilated, filled and eroded to find a binary mask for each cell area. The cell-edge was then defined as the perimeter of pixels at the boundary of the cell area in the binary mask.

In case of 3D cell spreading, large volumes of hydrogel were imaged and this required customized image analysis and edge detection per individual cell. For every spot in each z-slice where initially (part of) a cell was recognized, the pixel intensities of a larger area around the cell was extracted from the original image and saved in a database. Thereby a library of small and single cell images was created for every z-slice. By comparing the centroids (in pixel number) of all recognized cells across all the different z-slices, edges belonging to the same cell were identified as a single cell and their pixels linked to create single cell z-stacks. These single cell (mini) z-stack images were then, one-by-one, automatically presented to the user via a graphical-user-interface with the option to adjust detection parameters to best determine cell edges. Every cell analyzed was manually checked for correct edge-recognition, thereby eliminating any chance of erroneous detection due to highly varied fluorescence intensities. In the final step, each individual cell z-stack was maximally projected to obtain a 2D representation of the cell edge. This cycle can be repeated multiple times with different starting parameters, allowing all detectable cells to be added to the dataset.

Using the maximally projected binary mask of each cell, the centroid (center of mass), area (A), perimeter (P), total skeleton length (S) and ferret-diameters (F_{min} and F_{max}) were calculated using the appropriate scale (0.68 μm/pix). The circularity (C) and aspect ratio (AR) are calculated by $C = 4\pi A/P^2$ and $AR = F_{min}/F_{max}$. The error estimates in **Tables S3-S6** are the 95% confidence intervals of the mean ($\bar{x} \pm t_{n-1}\, s/\sqrt{n}$) using a t-distribution with $n-1$ degrees of freedom, where n is the sample size and s is the standard deviation. All cell edges of touching cells or cells in close proximity ($\pm$ 50 μm) to each other, were discarded.

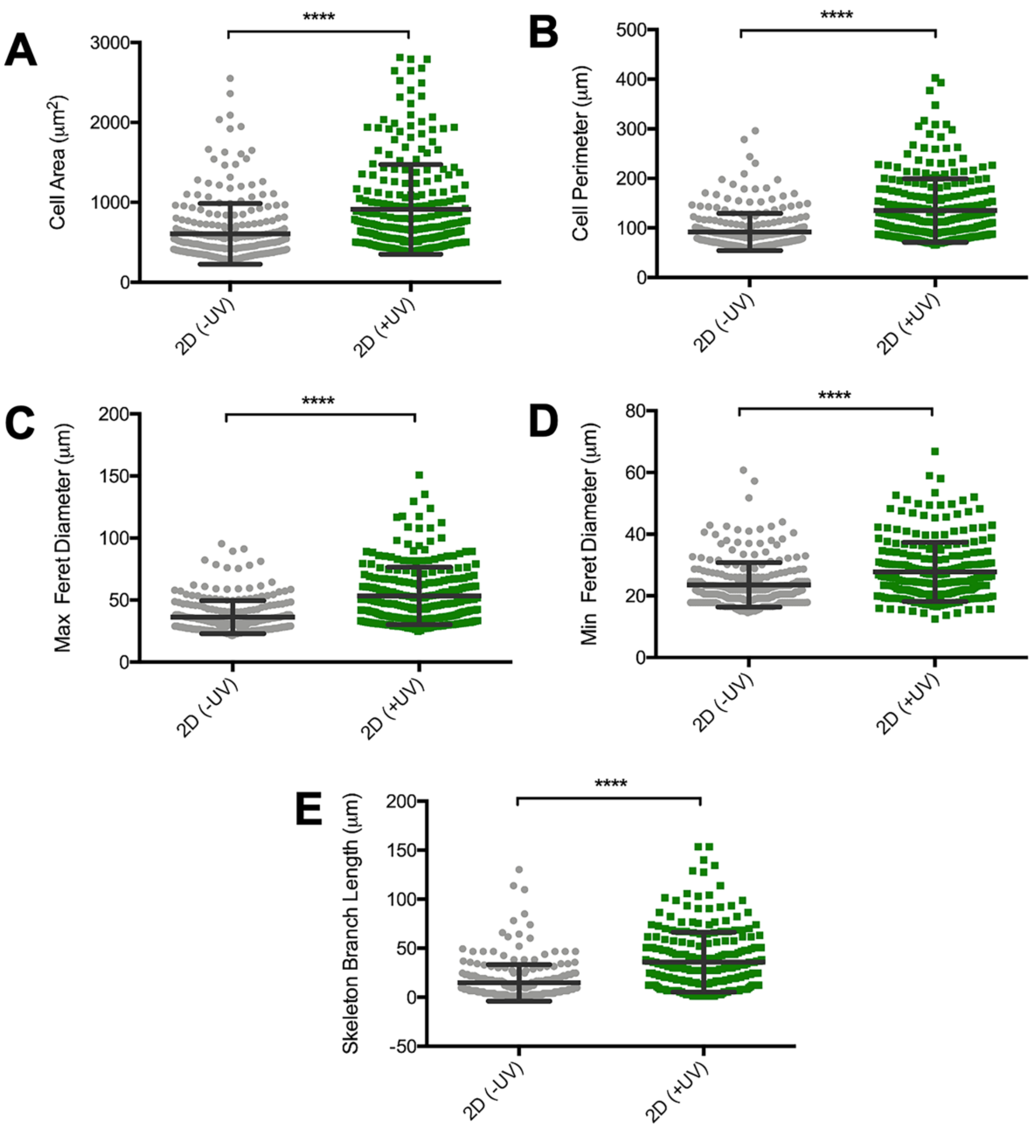

Figure S25. Quantitative analysis of cell area (A), cell perimeter (B), max Feret diameter (C), min Feret diameter (D), and skeleton branch length (E), of mCherry-LifeAct Hs578T cells (N = 255) after 6 h of culture on top of **SQ/10SQ-DT/10SQ-RGD** (4.0 mM) hydrogel with different UV irradiation times (0 min and 5 min). The mean and standard deviation are marked within the figures (****P < 0.0001 one-way Anova). UV irradiation was applied through a benchtop LED (~10 mW/cm^2, 375 nm).

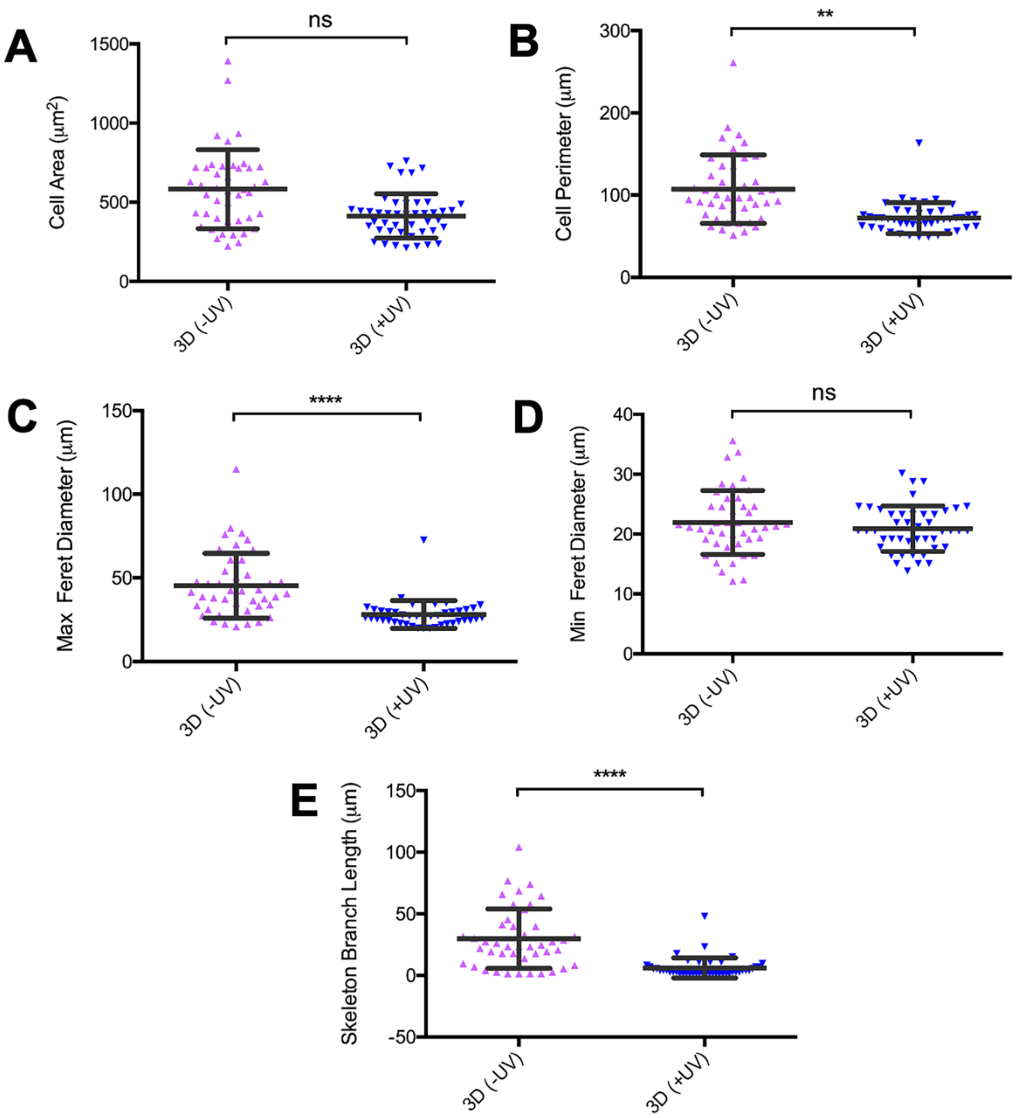

Figure S26. Quantitative analysis of the cell area (A), cell perimeter (B), max Feret diameter (C), min Feret diameter (D), and skeleton branch length (E), of mCherry-LifeAct Hs578T cells (N = 44) after 3 days of 3D culture in **SQ/10SQ-DT/10SQ-RGD** (4.0 mM) hydrogel with different UV irradiation time (0 min and 5 min). The mean and standard deviation are marked within the figures (**P < 0.01, ****P < 0.0001 one-way Anova). UV irradiation was applied through a benchtop LED (~10 mW/cm^2, 375 nm).

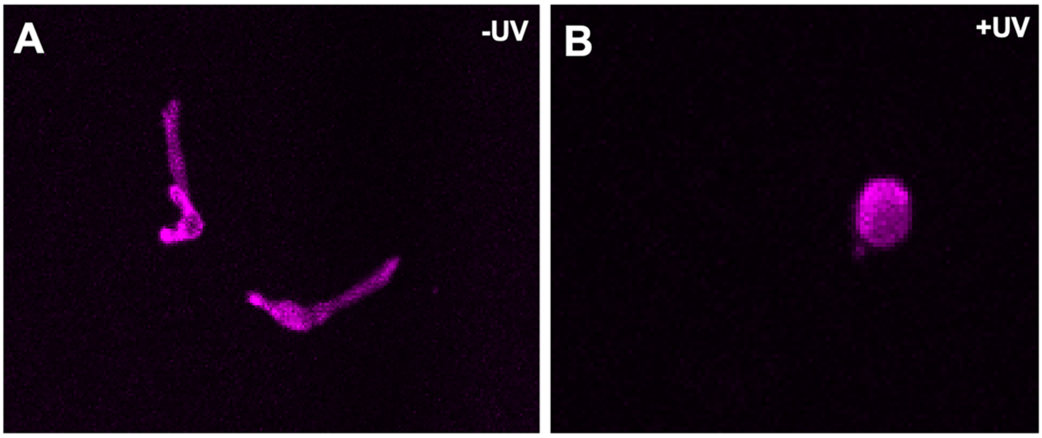

Figure S27. Representative confocal images of encapsulated mCherry-LifeAct Hs578T breast cancer cells within supramolecular hydrogel **SQ/10SQ-DT/10SQ-RGD** (4.0 mM) with and without UV irradiation: (A) 0 min, and (B) 5 min using a benchtop LED source (~10 mW/cm^2, 375 nm). Cells were cultured for 3 days after UV exposure and then imaged.

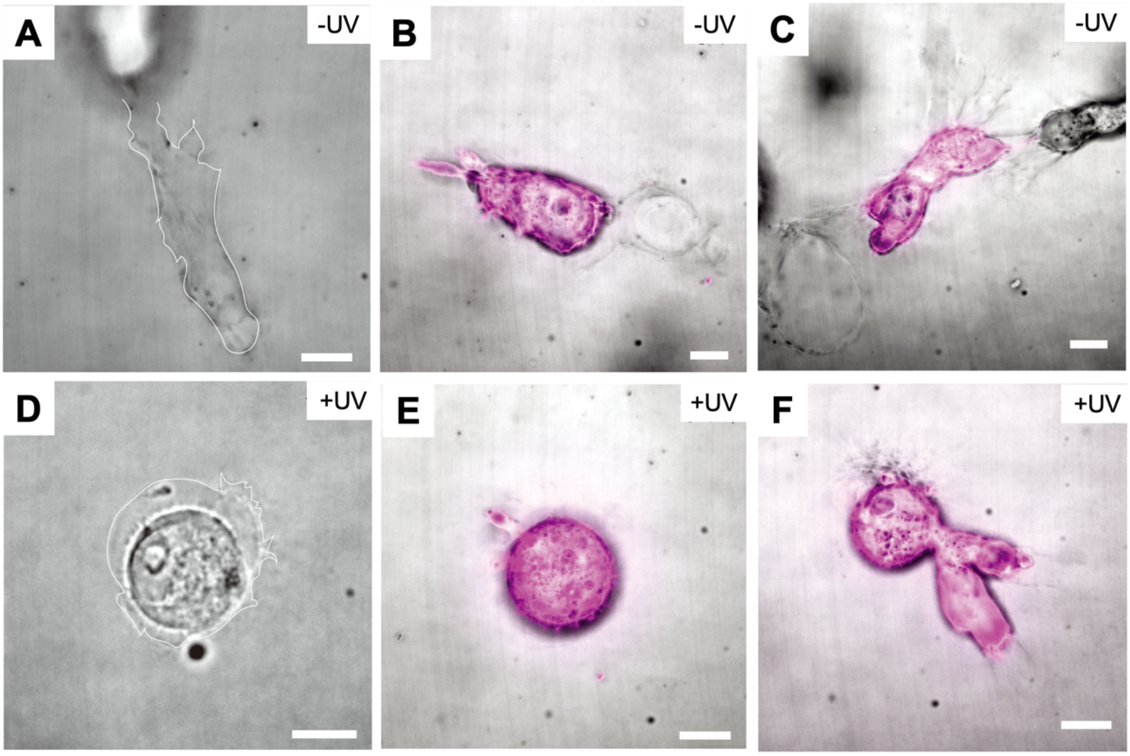

Figure S28. Hs578T invasiveness in 3D: The plasticity of the **SQ/SQ-DT/SQ-RGD** (4.0 mM) hydrogel influences cell spreading and migration. (A) Prior to UV irradiation the low stiffness and high plasticity allows Hs578T cells to remodel the hydrogel, spread and migrate. The cavity left behind by the cell is outlined in white. (B) and (C) representative examples of Hs578T spreading and motility inside non-UV irradiated hydrogel. The remodeled hydrogel left behind is clearly visible in the minimum intensity Z-projected bright field images. Actin (LifeAct RFP) polymerization is shown in magenta. (D) After UV irradiation, stiffness increases and plasticity decreases preventing Hs578T spreading and migration. Cells attempt to remodel the surrounding material, but are only moderately successful and unable to migrate. The plastically deformed gel is outlined in white. (E) and (F) Examples of Hs578T attempting to invade the UV stiffened and low plasticity gel by forming protrusions. Scale bar: *10 μm*.

Table S3. Summary of quantitative analyses (mean ± 95% confidence intervals) from mCherry-LifeAct Hs578T cells after 2D for 6 h cultured on **SQ/10SQ-DT/10SQ**-RGD (4.0 mM) hydrogel using different UV irradiation times (0 min and 5 min) from a benchtop LED (~10 mW/cm, 375 nm).

Parameters	0 min UV irradiation	5 min UV irradiation
Area (μm^2)	605.62 ± 46.93	912.66 ± 69.12
Perimeter (μm)	91.80 ± 4.62	135.15 ± 7.93
Circularity	0.89 ± 0.022	0.68 ± 0.031
Max Feret Diameter (μm)	36.19 ± 1.65	53.44 ± 2.86
Min Feret Diameter (μm)	23.56 ± 0.89	27.77 ± 1.18
Aspect Ratio	0.68 ± 0.016	0.56 ± 0.020
Skeleton Branch Length (μm)	14.81 ± 2.31	35.88 ± 3.76

Table S4. Summary of quantitative analyses (mean ± 95% confidence intervals) from mCherry-LifeAct Hs578T cells after 3D for 3 days cultured on **SQ/10SQ-DT/10SQ-RGD** (4.0 mM) hydrogel using different UV irradiation times (0 min and 5 min) from a benchtop LED (~10 mW/cm^2, 375 nm).

Parameters	0 min UV irradiation	5 min UV irradiation
Area (μm^2)	582.82 ± 75.74	413.47 ± 42.34
Perimeter (μm)	107.24 ± 12.64	72.09 ± 5.74
Circularity	0.70 ± 0.079	0.98 ± 0.044
Max Feret Diameter (μm)	45.30 ± 5.89	28.12 ± 2.51
Min Feret Diameter (μm)	21.95 ± 1.62	28.12 ± 2.51
Aspect Ratio	0.55 ± 0.059	0.76 ± 0.033
Skeleton Branch Length (μm)	29.80 ± 7.32	6.07 ± 2.47

S14. mCherry-LifeAct Hs578T cell migration in supramolecular hydrogels

S14.1. 3D cell encapsulation for migration studies

To obtain the 3D cell-laden supramolecular hydrogels for the migration study, mCherry-LifeAct Hs578T cells were encapsulated into multicomponent hydrogels without and with RGD peptide, namely, **SQ/10SQ-DT** (4.0 mM) and **SQ/10SQ-DT/10SQ-RGD** (4.0 mM), following the same procedure as mentioned above (see section **S11**). Various stiffnesses of the hydrogel were obtained with different light irradiation times (e.g. 0 min or 5 min) using a benchtop LED (~10 mW/cm^2, 375 nm). The supramolecular hydrogels were then stiffened at user-defined time points (day 0 or day 2) after seeding.

S14.2. Imaging and analysis of cell migration

Cell-laden supramolecular hydrogel samples were imaged using a 10x NA 0.3 objective on a confocal spinning-disk microscope. The mCherry-LifeAct Hs578T cells were imaged every 5 min over 16 hours using an exposure time of 200 ms. The fluorophore was excited using a 561 nm laser at 2.6 mW. For each sample, 1-8 positions with the volume slices (701 x 701 x 160 μm) were chosen and imaged using z-stacks at a scan speed of 10 μm/step. To investigate the

influence of temporal stiffening of the hydrogel on cell migration, the time-lapse images of the samples in the same position before and after UV irradiation were also collected.

In order to create a two-dimensional time-series of cell motion, the image stacks were maximally z-projected at every position using ImageJ (http://imagej.nih.gov/ij/). The images were then binarized by intensity thresholding and contrast adjustment. To determine the x, y-coordinates of the center-of-mass of each cell in each frame the cell evaluator plugin[8] in ImageJ was used. By linking all these x, y-positions together the cell trajectories were obtained and plotted in **Figure 5** and **Figure S29**.

The trajectories were analyzed using an in-house Matlab (version 2019b, The Mathworks) code. The 2D-projected instantaneous velocity was used to character the migratory activity of the cells and the MSD was a measure of the total space explored by the cell over time. The cell trajectories and frame rate between images were used to calculate the above parameters. The displacement (r) of the cell at time (t) between frames (Δt) was given by $r(t) = R(t + \Delta t) - R(t)$, where $R(t)$ are the vectors described by the x, y-coordinates of the cell center in each image. Then, the instantaneous velocity ($v(t)$) was defined by $v(t) = r(t)/\Delta t$ and the MSD ($\langle r(\tau)^2 \rangle$) by $\langle r(\tau)^2 \rangle = 1/N - k \sum_{i=1}^{N-k} (R(t_i + \tau) - R(t_i))^2$. Here, $\tau = k\Delta t$ is the lag-time, N the number of points in a trajectory, and k the frame number (maximally $k = 1,2,..N - 1$). The calculated instantaneous velocities and MSD of cell trajectories, before and after hydrogel stiffening, were shown in **Figure 5D**. Finally, the maximal displacement (with respect to the origin) of the cells within the various hydrogels (e.g. without/with RGD peptide and without/with UV irradiation) during the time-lapse was calculated, which was a measure of the distance explored by the moving cell (**Figure 5E**). The inclusion of RGD was found to be beneficial for cell viability during the stress of 48 hr time lapse (561 nm, 16 x 10 μm z-stacks, Δt = 5 min), as it increased from 65% for **SQ/10SQ-DT** to 100% in case of **SQ/10SQ-DT/10SQ-RGD** (**Figure S29**).

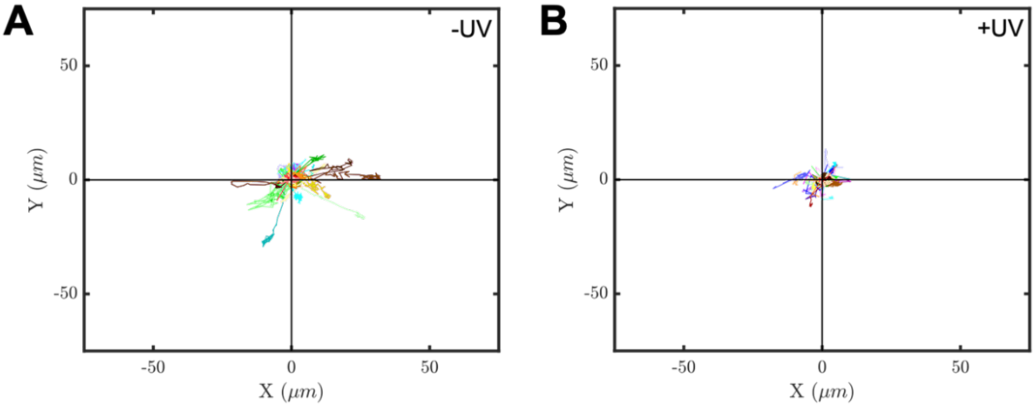

Figure S29. Representations of mCherry-LifeAct Hs578T cells trajectories within multicomponent hydrogels without RGD peptide **SQ/10SQ-DT** (4.0 mM): (A) cells (N = 58) traced for 24-48 h within hydrogel without UV irradiation, and (B) cells (N = 37) traced for 48-72 h after with UV irradiation (5 min) at a pre-determined time point (48 h). UV irradiation was applied through a benchtop LED (~10 mW/cm^2, 375 nm).

S15. Spatiotemporal photopatterning of bioactive cues

S15.1. Preparation of custom well inserts

All photopatterning and cell encapsulation experiments were performed in small custom-made well inserts placed inside larger imaging dishes (uncoated 35 mm dishes or uncoated μ-slide 4 well plates) or on top of microscopy slides (#1.5, 30 mm). This strategy facilitated using smaller

quantities of hydrogel (~ 30 μL) inside a relatively large (500-2000 μL) liquid reservoir, which enabled easy medium refreshment during hydrogel encapsulated cell culture, or PBS washing in the case of photopatterning experiments.

Briefly, the well inserts were cut from polydimethylsiloxane (PDMS) sheets produced on silicon wafers. A silicon wafer was first silanized by chemical vapor deposition of 1H, 1H, 2H, 2H-perfluorooctyltrichlorosilane under vacuum for one hour to ensure a proper PDMS detachment later. The pre-crosslinked PDMS (mixed with the curing agent under 1:10) was spin-coated on top of a silicon wafer to ensure a constant height. The PDMS on the wafer was put under vacuum (50 mbar) to degas. After baking at 100 °C for 6 hours, the custom wells were cut, washed with 70% ethanol, dried, and placed inside the designed plate. To increase the surface hydrophilicity of both the cell culture chamber and PDMS inset, the imaging chamber was exposed to UV-ozone surface treatment for 30 min before use.

S15.2. Preparation of supramolecular hydrogel and fluorescein RGD peptide mixtures

To study and visualize the patterning of bioactive cues in the supramolecular hydrogel through their chemical crosslinking using light, a fluorescent RGD peptide (**(Fluorescein)GK(DT)GGGRGDS**) was added. After sonication in an ice bath, a pre-gel precursor solution of **SQ/10SQ-DT** (6.66 mM, 135 μL) was mixed with different concentrations of the fluorescent RGD peptide (0.01 mM, 0.05 mM and 0.1 mM, 15 μL) by gentle pipetting (10 times). The obtained hydrogel and peptide mixtures were incubated at 37 °C for 15 min to trigger gel formation and left to equilibrate overnight at room temperature prior to use for photopatterning experiments. All photoactive samples were shielded from light during preparation prior to photopatterning. Finally, the equilibrated hydrogel mixtures, containing **SQ/10SQ-DT** (6.0 mM) and **(Fluorescein)GK(DT)GGGRGDS** (0.01 mM) were then photopatterned by application of light with either a photomask or direct laser writing.

To further calibrate the degree of crosslinking in photopatterned gels, supramolecular hydrogel mixtures containing known concentrations of **(Fluorescein)GK(DT)GGGRGDS** (e.g. 1.0 μM, 5.0 μM and 10.0 μM) were prepared and reacted with UV light.

S15.3. Supramolecular hydrogel photopatterning through a photomask

The pre-prepared hydrogel containing the fluorescent RGD peptide mixture (6.0 mM **SQ/10SQ-DT** with 0.01 mM **(Fluorescein)GK(DT)GGGRGDS**, 30 μL) was pipetted into a well insert inside the μ-slide 4 well plate and irradiated with UV light through a photomask (250 μm stripes) with a benchtop LED source (5 min, ~10 mW/cm^2, 375 nm). To wash out the unbound fluorescent RGD peptide, the hydrogels were submerged in PBS (800 μL) and incubated at 37 °C. In the first 4 h, the PBS was changed every hour. The samples were then incubated overnight at 37 °C before imaging.

S15.4. Two-photon crosslinking of supramolecular hydrogels using direct laser writing (DLW)

Identical to the photopatterning experiments using a photomask, the fluorescent RGD peptide and hydrogel mixture (6.0 mM **SQ/10SQ-DT** with 0.01 mM **(Fluorescein)GK(DT)GGGRGDS**) was pipetted (30 μL) into a custom cut PDMS well insert placed on a glass microscope cover slips (#1.5, 30 mm). To keep the hydrogel submerged in PBS during the writing process, a second PDMS well insert was placed on top of the first insert (with the gel inside). Finally, the two-stage PDMS well insert (30 μL reservoir 1 with hydrogel, 100 μL reservoir 2 with PBS inside), was sealed with a second glass cover slip to prevent evaporation.

Two-photon crosslinking by direct laser writing (DLW) was performed using the Photonics Professional GT. 3D structures were designed in Autodesk Inventor and converted to the stereolithography file format (STL). Then, STL files were imported into DeScribe to

obtain a suitable mesh for DLW. The hydrogel and crosslinker composite were exposed to a 780 nm laser (Ti-Sapphire, 20 mW maximum at sample surface) using a 20x Air objective. In the x, y-directions the DLW mesh was scanned by the laser through galvanic mirrors, and then stitched, slice-by-slice, in the z-direction with a piezo stage. Scanning speed was set at 250 μm/s with a power scaling of 1.00. All DLW procedures were performed under yellow light (λ = 577-597 nm) to prevent spontaneous crosslinking during the writing process. The samples were washed with PBS (5 times) and kept in PBS at room temperature overnight before imaging.

S15.5. Confocal imaging of photopatterned hydrogels

The photopatterned hydrogels were imaged using a 10x and 20x objective on a Nikon Eclipse Ti microscope equipped with a Yokogawa confocal spinning disk unit operated at 10,000 rpm. The covalently bound fluorescein was excited with a 0.2 mW, 488 nm laser light from solid state diode laser supported in an Agilent MLC4 unit. Images were captured using an exposure time of 200-300 ms by an Andor iXon Ultra 897 high speed EM-CCD camera. The acquired fluorescent images were background-corrected to visualize the fluorescent RGD crosslinking.

S15.6. Quantification of photopatterning efficiency of the RGD peptide within the hydrogel

The fraction of **DT-RGD** peptide bound to the hydrogel can be quantified using the fluorescent RGD peptide (**(fluorescein)GK(DT)GGGRGDS**). The emission intensity of hydrogel mixtures upon laser excitation is directly proportional to the concentration of fluorescein present. Therefore, samples with different known concentrations and homogenous UV illumination were used to calibrate and estimate the fraction of **DT-RGD** bound in photopatterning experiments.

The fluorescence emission of the patterned hydrogel was acquired by spinning-disk confocal microscopy and was measured for two objectives (10x NA 0.3, and 20x NA 0.75) across a wide range of 488 nm excitation intensities (laser power) for two different samples with the fluorescent RGD peptide (10 μM) (**Figure S30A**). To assist in later calibrations, a quadratic fit (with degree fixed at 0) was done to inter- and extrapolate the intensity of fluorescent emission as a function of laser power. Next to different laser powers, the effect of varying fluorescein concentration on emission intensity was also measured (**Figure S30B**) for each objective and laser power. The fluorescence emission was found to be linearly proportional to the concentration. All intensities shown are averaged over a block of 100 x 100 pixels in the center of the field-of-view (512 x 512) of the camera.

After calibration measurements, the supramolecular hydrogels containing the fluorescent RGD peptide were uniformly irradiated with UV light for 5 min using a benchtop LED (~10 mW/cm^2, 375 nm). Subsequently, the samples were covered with PBS to wash out the unbound peptide. The samples were imaged after washing for 4 h and after further washing for 24 h. During the first 4 h, the samples were exchanged with fresh PBS hourly. The emission intensities of the samples before (*black line*) and after UV irradiation (*red line*) are shown in **Figure S31**. All normalized intensities were separately scaled (**Figure S31**) using the intensity measured before UV irradiation for each concentration respectively. The fraction of fluorescent RGD peptide bound was 0.2 for all measured concentrations. The yield of light-induced crosslinking is directly proportional to the RGD concentration used when homogenous UV irradiation is applied. Furthermore, extensive washing for a longer time (24 h) did not influence the retention of the fluorescent RGD peptide (**Figure S31**, *green line*).

Using the calibration experiments shown above, the retention of fluorescent RGD peptide within the patterned hydrogels was estimated. A 10x objective was used to collect the calibration data for the samples where a photomask was applied and a 20x objective (data not shown) for the DLW-patterned samples. In both cases, a supramolecular hydrogel concentration of 6.0 mM **SQ/10SQ-DT** with 1.0 μM fluorescent RGD peptide was examined. The calibration

experiment was used to relate the imaged fluorescence intensity to the concentration of RGD peptide present. The hydrogel samples were imaged at a laser power of 1.44 mW and the concentration of fluorescent RGD peptide bound in the samples patterned through a photomask (**Figure S32**) or using DLW (**Figure S33**) was estimated from the images.

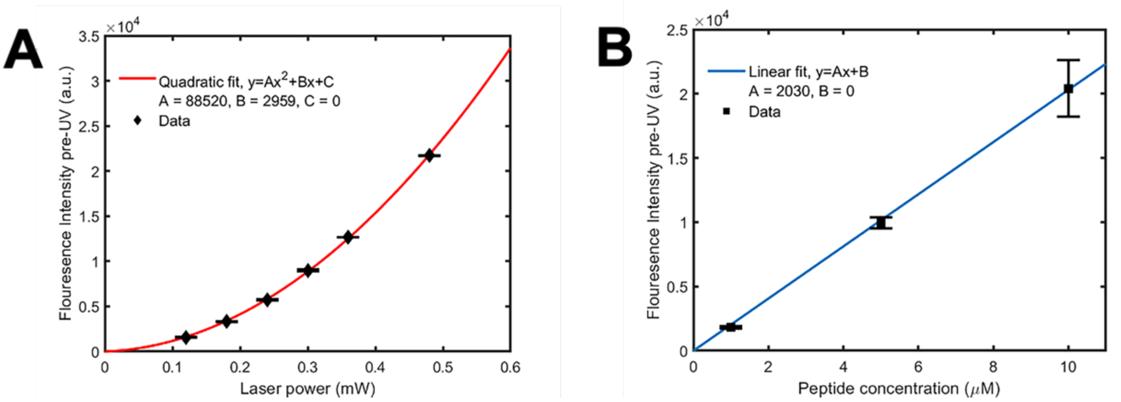

Figure S30. Calibration results of the mixtures of hydrogel **SQ/10SQ-DT** (6.0 mM) with different concentrations of fluorescent RGD peptide (**(fluorescein)GK(DT)GGGRGDS**) (1 μM, 5 μM and 10 μM) in the unbound state. (A) Fluorescence emission intensity of hydrogel containing fluorescent RGD peptide (10 μM) before UV exposure as a function of the laser power (excitation intensity) using a 10x objective. A quadratic fit (C = 0) was used to inter- and extrapolate intensities of other laser powers. (B) Fluorescence emission intensity of fluorescent RGD peptide before UV exposure as a function of its concentration (1 μM, 5 μM and 10 μM). A linear fit (B = 0) was used to inter- and extrapolate intensities of different concentrations. Error bars are standard deviations over measurements performed.

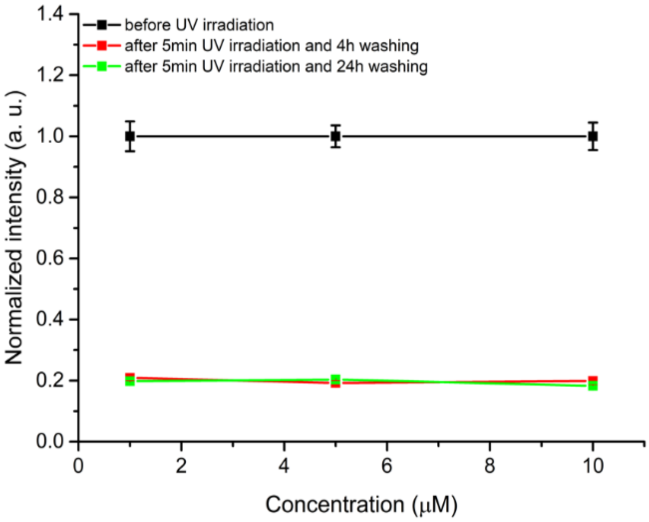

Figure S31. Normalized fluorescence intensity of multicomponent hydrogel **SQ/10SQ-DT** (6.0 mM) containing different concentrations of fluorescent RGD peptide (**(fluorescein)GK(DT)GGGRGDS**) (1 μM, 5 μM and 10 μM) before and after 5 min UV irradiation using a benchtop LED source (~10 mW/cm^2, 375 nm), and further washing with PBS for 4 h and 24 h. The intensity of each sample after UV irradiation was separately normalized to its value of fluorescence intensity before UV irradiation. Error bars are standard deviations over measurements performed.

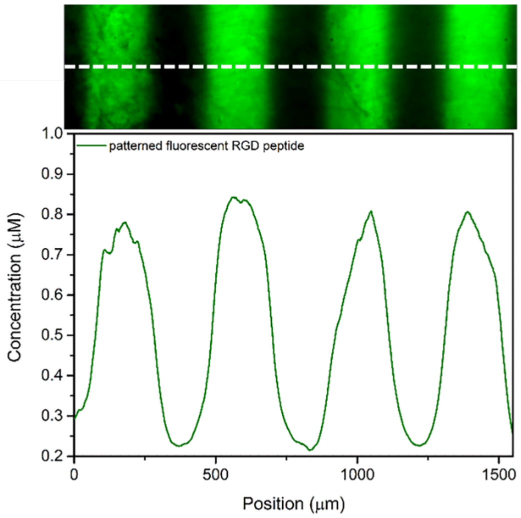

Figure S32. 2D confocal fluorescence images of a fluorescent RGD peptide (**(Fluorescein)GK(DT)GGGRGDS**) patterned within hydrogel **SQ/10SQ-DT** (6.0 mM) under photomask for 5 min UV irradiation through a benchtop LED (~10 mW/cm^2, 375 nm) and the presented patterned RGD concentration in (un)patterned areas.

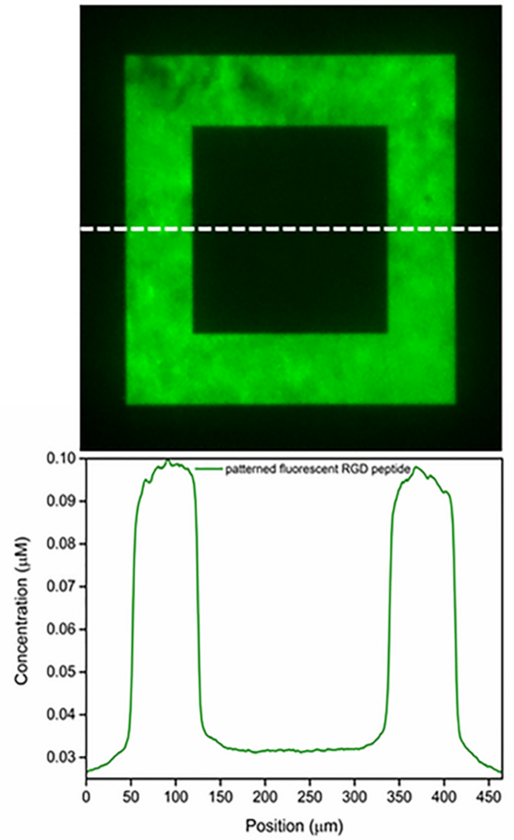

Figure S33. 2D confocal fluorescence images of fluorescent RGD peptide (**(Fluorescein)GK(DT)GGGRGDS**) patterned within hydrogel **SQ/10SQ-DT** (6.0 mM) via direct-laser writing (DLW) and the coupled RGD concentration varied with the position of (un)patterned areas.

S15.7. Examination of cell shape in 3D after in situ post-functionalization of the supramolecular network with the RGD peptide using light

To obtain uniform peptide-functionalized supramolecular hydrogels, the pre-gel precursor solution (after sonication in ice water) **SQ/10SQ-DT** (3.7 mM, 135 μL) was mixed with the cell-adhesion **DT-RGD** (4.44 mM, 15 μL) or the scrambled **DT-DGR** (4.44 mM, 15 μL) peptides by gentle pipetting (~10 times) to yield a solution of **SQ/10SQ-DT** (3.33 mM) and **DT-RGD** (0.44 mM) or **DT-DGR** (0.44 mM). Hydrogels were formed after 15 min incubation at 37 °C and left to equilibrate overnight at room temperature prior to further use.

To seed the cells in supramolecular hydrogels, the pre-made gels (135 μL) were mixed with a C2C12 cell suspension (5.0 x 10^6 cells/mL, 15 μL) by gentle pipetting (~10 times), and then incubated at 37 °C for 15 min. The final cell-laden hydrogels of **SQ/10SQ-DT** (3.0 mM) with **DT-RGD** or **DT-DGR** (0.4 mM) contained a cell density of 5.0 x 10^5 cells/mL. The obtained hydrogels (12 μL) were then pipetted into a μ-slide 15-well plate and left to stand in the incubator at 37 °C for 5 min. The cell-laden hydrogels were then irradiated with UV light for different durations (0 min and 5 min) using a benchtop LED source (~10 mW/cm^2, 375 nm). Cell culture media (48 μL) was layered on top of the hydrogels, and then they were cultured in an incubator at 37 °C until further use. Cell culture media was refreshed every two hours during the first 6 h, to wash out the uncoupled **DT-RGD** or **DT-DGR** after UV irradiation. During the following culture days, media was replaced daily. On day 3, cell viability and morphology were measured by confocal laser scanning microscopy after staining with calcein AM/propidium iodide (PI) as described in section **11**.

S15.8. Photopatterning of RGD in the presence of cells using a photomask

Photopatterned supramolecular hydrogel samples were prepared similarly to the uniformly stiffened RGD peptide gels as described above with the addition of the fluorescent RGD peptide **(Fluorescein)GK(DT)GGGRGDS** (1.0 μM) in the supramolecular hydrogel/**DT-RGD** mixture. The same total RGD concentration (0.4 mM) is maintained in this experiment. The fluorescent RGD peptide was added here to visualize the RGD photopatterned areas. Pre-made cell-hydrogels were pipetted into a μ-slide 4-well plate with custom made PDMS well insets (produced as described in section **S1.15**) and then irradiated with UV light for 5 min in the presence of a photomask (250 μm stripes) using a benchtop LED source (~10 mW/cm^2, 375 nm). The cell-laden hydrogels were submersed in culture media (800 μL) and refreshed hourly during the first 4 hours of incubation to wash out unbound **DT-RGD** and **(Fluorescein)GK(DT)GGGRGDS** peptide. Finally, the samples were left to incubate at 37 °C and 5% CO_2 prior to imaging. During culture, media was replaced daily.

On day 3, immunostaining was performed on the C2C12 cells in the photopatterned supramolecular hydrogels. First, the samples were fixed with a 4 wt% paraformaldehyde solution in PBS (800 μL) at room temperature for 30 min. The samples were then washed with PBS (4 x 800 μL), followed by cell membrane permeabilization using a 0.1% TritonX-100 solution in PBS (800 μL) at room temperature for 2 h. Samples were washed with PBS (4 x 800 μL) and non-specific protein interactions were blocked using 2% bovine serum albumin (BSA) (800 μL) at room temperature for 2 h. Samples were washed with PBS (4 x 800 μL) and then F-actin stained using AlexaFluor488-phalloidin antibody (1:300) in 0.1% BSA overnight at 4 °C. Finally, the samples were further washed with PBS (4 x 800 μL). The stained samples were covered with fresh PBS and stored at 4 °C before imaging.

Table S5. Summary of quantitative analyses of the C2C12 cells after 3 days culture within the supramolecular hydrogel (3.0 mM **SQ/10SQ-DT**) in 3D containing 0.4 mM **DT-RGD** and the fluorescent RGD peptide photopatterned using a photomask (200 µm stripes) under UV light using a benchtop LED source (~10 mW/cm^2, 375 nm) for 5 min.

Parameters	RGD patterned areas (*green*)	Non-RGD patterned areas (*black*)
Area (µm^2)	657 ± 27	568 ± 38
Perimeter (µm)	108 ± 4	92 ± 5
Circularity	0.805 ± 0.019	0.906 ± 0.023
Max Feret Diameter (µm)	44 ± 2	37 ± 2
Min Feret Diameter (µm)	22 ± 1	23 ± 1
Skeleton Branch Length (µm)	23 ± 2	22 ± 3

S15.9. Morphology of mCherry-LifeAct Hs578T breast cancer cells in photopatterned RGD-supramolecular hydrogels

Photopatterned RGD supramolecular hydrogels containing **SQ/10SQ-DT** (3.0 mM) and RGD peptide (**DT-RGD** (0.399 mM) and **(fluorescein)GK(DT)GGGRGDS** (0.001 mM)) were prepared to encapsulate mCherry-LifeAct Hs578T breast cancer cells as described earlier. On day 3, the cell morphology of mCherry-LifeAct Hs578T cells in the photopatterned supramolecular hydrogels was imaged.

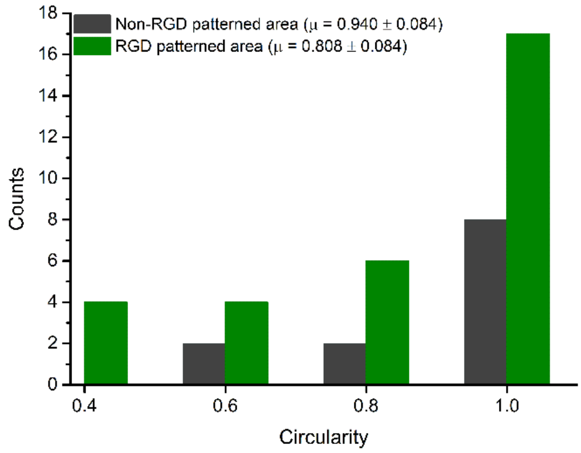

Figure S34. Cell circularity of mCherry-LifeAct Hs578T breast cancer cells in the RGD patterned (*green*) and unpatterned areas (*black*) after 3 days encapsulation in **SQ/10SQ-DT** (3.0 mM) hydrogels containing RGD peptide (**DT-RGD** (0.399 mM) and fluorescent RGD peptide (**(fluorescein)GK(DT)GGGRGDS**) (0.001 mM)). UV irradiation was applied through a benchtop LED (~10 mW/cm^2, 375 nm) using a photomask (200 µm stripes).

Table S6. Summary of quantitative analyses of mCherry-LifeAct Hs578T breast cancer cells after 3 days culture within the supramolecular hydrogel (3.0 mM **SQ/10SQ-DT**) in 3D containing 0.4 mM **DT-RGD** and the fluorescent RGD peptide photopatterned using a photomask (200 μm stripes) under UV light using a benchtop LED source (~10 mW/cm^2, 375 nm) for 5 min.

Parameters	RGD patterned areas (*green*)	Non-RGD patterned areas (*black*)
Area (μm^2)	699 ± 153	752 ± 109
Perimeter (μm)	96 ± 12	117 ± 18
Circularity	0.940 ± 0.084	0.808 ± 0.084
Max Feret Diameter (μm)	36 ± 5	48 ± 8
Min Feret Diameter (μm)	26 ± 3	26 ± 2
Skeleton Branch Length (μm)	9 ± 5	28 ± 10

S16. References

(1) Tong, C.; Liu, T.; Saez Talens, V.; Noteborn, W. E. M.; Sharp, T. H.; Hendrix, M.; Voets, I. K.; Mummery, C. L.; Orlova, V. V.; Kieltyka, R. E. Squaramide-Based Supramolecular Materials for Three-Dimensional Cell Culture of Human Induced Pluripotent Stem Cells and Their Derivatives. *Biomacromolecules* **2018**, *19*, 1091-1099.

(2) Tong, C.; Wondergem, J. A. J.; Heinrich, D.; Kieltyka, R. E. Photopatternable, Branched Polymer Hydrogels Based on Linear Macromonomers for 3D Cell Culture Applications. *ACS Macro Lett.* **2020**, *9*, 882-888.

(3) Liu, J.; Hilderink, J.; Groothuis, T. A.; Otto, C.; van Blitterswijk, C. A.; de Boer, J. Monitoring nutrient transport in tissue-engineered grafts. *J. Tissue Eng. Regen. Med.* **2015**, *9*, 952-960.

(4) Blonk, J. C. G.; Don, A.; Van Aalst, H.; Birmingham, J. J. Fluorescence photobleaching recovery in the confocal scanning light microscope. *J. Microsc.* **1993**, *169*, 363-374.

(5) 5. Koedoot, E.; Fokkelman, M.; Rogkoti, V. M.; Smid, M.; van de Sandt, I.; de Bont, H.; Pont, C.; Klip, J. E.; Wink, S.; Timmermans, M. A.; Wiemer, E. A. C.; Stoilov, P.; Foekens, J. A.; Le Devedec, S. E.; Martens, J. W. M.; van de Water, B. Uncovering the signaling landscape controlling breast cancer cell migration identifies novel metastasis driver genes. *Nat. Commun.* **2019**, *10*, 2983.

(6) Peng, T.; Thorn, K.; Schroeder, T.; Wang, L.; Theis, F. J.; Marr, C.; Navab, N. A BaSiC tool for background and shading correction of optical microscopy images. *Nat. Commun.* **2017**, *8*, 14836.

(7) Youssef, S.; Gude, S.; Radler, J. O. Automated tracking in live-cell time-lapse movies. *Integr. Biol.* **2011**, *3*, 1095-1101.

(8) Yu, H.; Wang, Y.; Yang, H.; Peng, K.; Zhang, X. Injectable self-healing hydrogels formed via thiol/disulfide exchange of thiol functionalized F127 and dithiolane modified PEG. *J. Mater. Chem. B* **2017**, *5*, 4121-4127.
